# Cardiac-specific deletion of voltage dependent anion channel 2 leads to dilated cardiomyopathy by altering calcium homeostasis

**DOI:** 10.1038/s41467-021-24869-0

**Published:** 2021-07-28

**Authors:** Thirupura S. Shankar, Dinesh K. A. Ramadurai, Kira Steinhorst, Salah Sommakia, Rachit Badolia, Aspasia Thodou Krokidi, Dallen Calder, Sutip Navankasattusas, Paulina Sander, Oh Sung Kwon, Aishwarya Aravamudhan, Jing Ling, Andreas Dendorfer, Changmin Xie, Ohyun Kwon, Emily H. Y. Cheng, Kevin J. Whitehead, Thomas Gudermann, Russel S. Richardson, Frank B. Sachse, Johann Schredelseker, Kenneth W. Spitzer, Dipayan Chaudhuri, Stavros G. Drakos

**Affiliations:** 1 Nora Eccles Harrison Cardiovascular Research and Training Institute, University of Utah, Salt Lake City, UT USA; 2 Department of Biomedical Engineering, University of Utah, Salt Lake City, UT USA; 3 Walther Straub Institute of Pharmacology and Toxicology, Faculty of Medicine, LMU Munich, Munich, Germany; 4 Department of Kinesiology, University of Connecticut, Storrs, CT USA; 5 Geriatric Research, Education, and Clinical Center, Salt Lake City VA Medical Center, Salt Lake City, UT USA; 6 Walter-Brendel-Center of Experimental Medicine, Ludwig-Maximilians Universität Munich, Munich, Germany; 7 German Centre for Cardiovascular Research (DZHK), Partner Site Munich Heart Alliance, Munich, Germany; 8 Department of Chemistry and Biochemistry, University of California, Los Angeles, CA USA; 9 Memorial Sloan Kettering Cancer Center, New York, NY USA; 10 Division of Cardiovascular Medicine, University of Utah School of Medicine, Salt Lake City, UT USA

**Keywords:** Molecular biology, Cardiology, Cardiomyopathies

## Abstract

Voltage dependent anion channel 2 (VDAC2) is an outer mitochondrial membrane porin known to play a significant role in apoptosis and calcium signaling. Abnormalities in calcium homeostasis often leads to electrical and contractile dysfunction and can cause dilated cardiomyopathy and heart failure. However, the specific role of VDAC2 in intracellular calcium dynamics and cardiac function is not well understood. To elucidate the role of VDAC2 in calcium homeostasis, we generated a cardiac ventricular myocyte-specific developmental deletion of *Vdac2* in mice. Our results indicate that loss of VDAC2 in the myocardium causes severe impairment in excitation-contraction coupling by altering both intracellular and mitochondrial calcium signaling. We also observed adverse cardiac remodeling which progressed to severe cardiomyopathy and death. Reintroduction of VDAC2 in 6-week-old knock-out mice partially rescued the cardiomyopathy phenotype. Activation of VDAC2 by efsevin increased cardiac contractile force in a mouse model of pressure-overload induced heart failure. In conclusion, our findings demonstrate that VDAC2 plays a crucial role in cardiac function by influencing cellular calcium signaling. Through this unique role in cellular calcium dynamics and excitation-contraction coupling VDAC2 emerges as a plausible therapeutic target for heart failure.

## Abstract (web summary)

The authors found that VDAC2 plays a crucial role in influencing mitochondrial calcium dynamics and cellular calcium signalling. A VDAC2 agonist, efsevin, rescued the heart failure phenotype, identifying a new potential therapeutic target for heart failure.

## Introduction

Non-ischemic dilated cardiomyopathy (DCM) is one of the most common causes that lead to the syndrome of chronic heart failure (HF) which is currently a growing global epidemic^1,2^. DCM is a condition in which the cardiac ventricular chambers enlarge and have impaired systolic and diastolic function. The impaired myocardial function has been partly attributed to alterations in the function of contractile proteins and excitation–contraction coupling (ECC)^3^. Calcium plays a crucial role in ECC and influences cardiac rhythmicity and cellular contraction. During systole, electrical excitation of the membrane causes L-type calcium channels (LTCC) to open and a small amount of calcium enters the cell which binds to ryanodine receptor 2 (RyR2) and triggers the sarcoplasmic reticulum (SR) to release some of its calcium reserve. This release results in an overall increase in the cytosolic calcium which binds to troponin thereby facilitating cellular contraction. The excess cytosolic calcium is pumped out of the cytoplasm through three main processes. Part of the calcium is pumped back into the SR through sarcoplasmic endoplasmic reticulum calcium ATPase 2a (SERCA2a) and the rest of the calcium is extruded out of the cell via the sodium–calcium exchanger (NCX1) and the sarcolemma calcium pump. The mitochondria also appear to play a role in calcium uptake during this process and its importance is under investigation^4,5^.

The voltage-dependent anion channel 2 (VDAC2) is a 32 kDa porin present on the mitochondrial outer membrane (MOM) and contributes to apoptosis, steroidogenesis, metabolite flux, and calcium homeostasis^6^. VDAC2 is known to interact with pro-apoptotic proteins such as Bcl2 family proteins. While VDAC2–BAK (Bcl2-antagonist/killer protein) interaction is reported to be highly important to control cellular apoptosis, it is still controversial if this interaction promotes or inhibits apoptosis. Studies show that VDAC2–BAK complex was absent during death stimulus and deletion of VDAC2 promotes apoptosis^7^. In contrast, other studies have shown that truncated-Bid (BH3-interacting domain death agonist) induced MOM permeabilization and cell death in wild-type (WT) but not in VDAC2 knock-out (KO) mouse embryonic fibroblasts^8^.

Calcium flux into the mitochondrial matrix takes place primarily through the mitochondrial calcium uniporter (MCU), present in the mitochondrial inner membrane (MIM). However, the initial calcium import into the inter-membrane space is through VDACs. The interaction between MOM and SR is crucial for this mitochondrial calcium signaling^9^. Evidence suggests that coupling of VDAC2 with sub-sarcolemmal RyR2 is essential for calcium transfer from SR to the mitochondria^10^. Mitochondrial calcium dynamics have mostly been associated with the MIM and primarily, the MCU. However, it has been reported that MCU-KO mice do not show any adverse cardiac phenotypes^11,12^. This suggests an important role of VDACs and MOM in mitochondrial calcium signaling.

Increasing evidence suggests the importance of VDAC2 in physiologic cardiac function and in this manuscript, we hypothesize that mitochondrial calcium dynamics have a significant influence in this process^13,14^. Increasing the calcium uptake through VDAC2 has been shown to reverse the arrhythmia phenotype observed in the tremblor zebrafish model (NCX1h mutant) and the tachycardia phenotype in mice with catecholaminergic polymorphic ventricular tachycardia (RYR2 mutant)^13,14^. Both these arrhythmic models have irregular cellular calcium signaling. Enhancing mitochondrial calcium uptake through VDAC2 using a small molecule called efsevin, pharmacologically helped restore the rhythmic phenotype in these models. VDAC2 knock-down in HL-1 cell line has shown to have specific effects on calcium homeostasis such as increased diastolic calcium and restricted calcium spark expansion^15^. However, after a global knockdown of VDAC2 was shown to be embryonic lethal^7^, no further studies have investigated the effects of cardiac VDAC2-KO in animal models. Advancing our understanding of VDAC2’s role in calcium signaling and ECC may have significant implications. In this study, in an effort to address this gap in knowledge we performed cardiac-specific *Vdac2* deletion and studied its effects on calcium cycling. Our results indicate a strong dependency on VDAC2 and cellular calcium dynamics to the ECC. VDAC2-KO mice showed significantly slower mitochondrial calcium uptake and altered cellular calcium signaling with smaller calcium transient, a slower rate of decay, and a slower rate of rising in cytosolic calcium. Adverse cardiac remodeling and severe cardiomyopathy led to the death of these KO mice. Reintroduction of VDAC2 in 6-week-old KO mice using an adeno-associated virus 9 (AAV9) vector seemed to partially rescue the cardiomyopathy phenotype suggesting a plausible role of VDAC2 as a therapeutic target in clinical HF. To evaluate this hypothesis, we took a gain-of-function approach and measured the effects of VDAC2 agonist efsevin in murine tissue from failing hearts: in line with the HF phenotype induced by VDAC2-KO, efsevin enhanced contractile force in failing myocardium from a murine pressure-overload model establishing VDAC2 as a promising target for HF.

## Results

### Cardiac-specific developmental deletion of VDAC2 causes DCM and leads to increased mortality

**Fig. 1:**
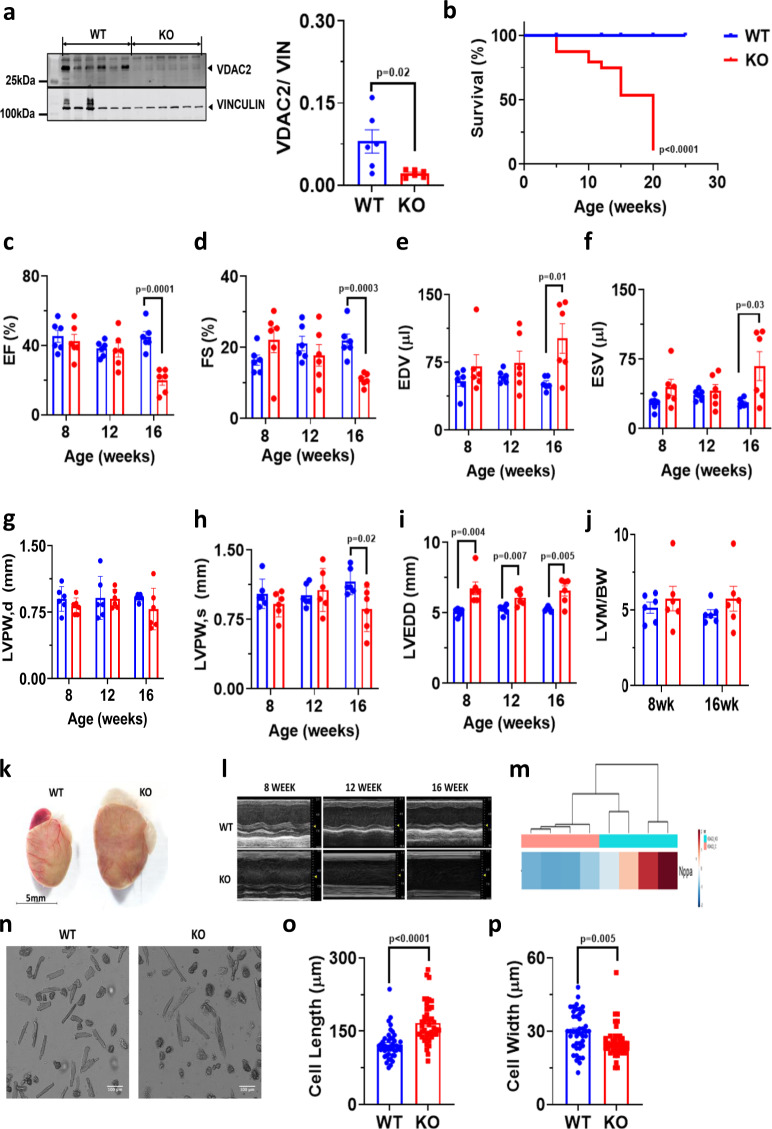
Cardiac-specific deletion of VDAC2 causes dilated cardiomyopathy and leads to increased mortality. **a** Western blot image of VDAC2 and lane-loading control VINCULIN and quantification in WT (Wild-type) and KO (Knock-out) using Image Studio Lite (version5.2.5) (*p* = 0.02) (*n* = 6); **b** Kaplan–Meier survival curve (*n* = 10) (*p* < 0.0001); **c**–**j** Echocardiography analysis of left ventricular ejection fraction (EF) (*p* = 0.000143), fractional shortening (FS) (0.000376), left ventricular end-diastolic and end-systolic volumes (EDV and ESV) (*p* = 0.0132 and *p* = 0.0311), left-ventricular posterior wall thickness at diastole and systole (LVPW,d and LVPW,s) (*p* = ns and *p* = 0.0244), left ventricular end-diastolic diameter (LVEDD) (8-week *p* = 0.004824; 12-week *p* = 0.007670 and 16-week *p* = 0.005986) (*n* = 6) and **j** left ventricular mass to body weight ratio (LVM/BW), respectively (*n* = 6); **k** Representative heart image of 16-week-old mice (scale bar, 5 mm); **l** Representative m-mode image from echocardiography (*n* = 1); **m** Differential expression of NPPA gene (*n* = 4); **n** Representative ventricular cardiomyocyte image using confocal microscopy (scale bar, 100 μm ); **o**, **p** Cardiomyocyte cell length (*p* < 0.0001) and width (*p* = 0.0058) measured using Zen5 imaging software, respectively (*n* = 42, *N* = 3). *p*-value: unpaired two-tailed *t*-test performed in panels **a**, **o**, and **p**, Mantel–Cox test performed in panel **b** and two-stage set-up multiple *t*-test (Benjamini, Krieger, and Yekutieli) performed for longitudinal echocardiographic data. Data are represented as differences between mean ± SEM.

Cardiac ventricle-specific developmental *Vdac2* KO and littermate WT were used for this study. Mice with either flox or Cre were used as WT and echocardiography data on flox or Cre only mice shows no significant differences (Supplementary Fig. 1a–g). Deletion of VDAC2 was confirmed using western blot (Fig. 1a). We observed no difference in VDAC1 and VDAC3 protein levels in VDAC2-KO mice (Supplementary Fig. 2a–c), suggesting a pure VDAC2-dependent phenotype. Increased mortality was observed in the KO between 5 and 20-weeks postpartum. Most of these events occurred during 20- weeks postpartum (Fig. 1b). Serial echocardiographic analysis, showed progressive deterioration of cardiac function in VDAC2-KO mice as reflected by significant reductions around 16 weeks in left ventricular (LV) ejection fraction (EF), fractional shortening (FS), and posterior wall thickness at systole (LVPW,s) and a significant increase in LV end-diastolic diameter (LVEDD), LV end-diastolic and end-systolic volumes (EDV and ESV, respectively). No difference in LV posterior wall thickness at diastole (LVPW,d) and LV mass (normalized to body weight) was observed (Fig. 1c–j). We are also providing Supplementary Table 1 which describes the cardiac phenotype during the entire development, including intra-utero echocardiograms performed in pregnant mice^16^. We also found increased gene expression of NPPA in KO mice (Fig. 1m), all of which are hallmarks of DCM. Increased dilation was also evident from echocardiography and the KO hearts were significantly larger than WT hearts (Fig. 1k, l). VDAC2-KO mice also had significantly longer and thinner cardiomyocytes compared to WT mice at 16-weeks of age (Fig. 1n–p). Taken together, our results suggest that deletion of VDAC2 in the myocardium during development causes DCM and eventually leads to increased mortality.

### Altered gene and protein expression profiles indicate altered calcium signaling and regulation in VDAC2-KO

**Fig. 2:**
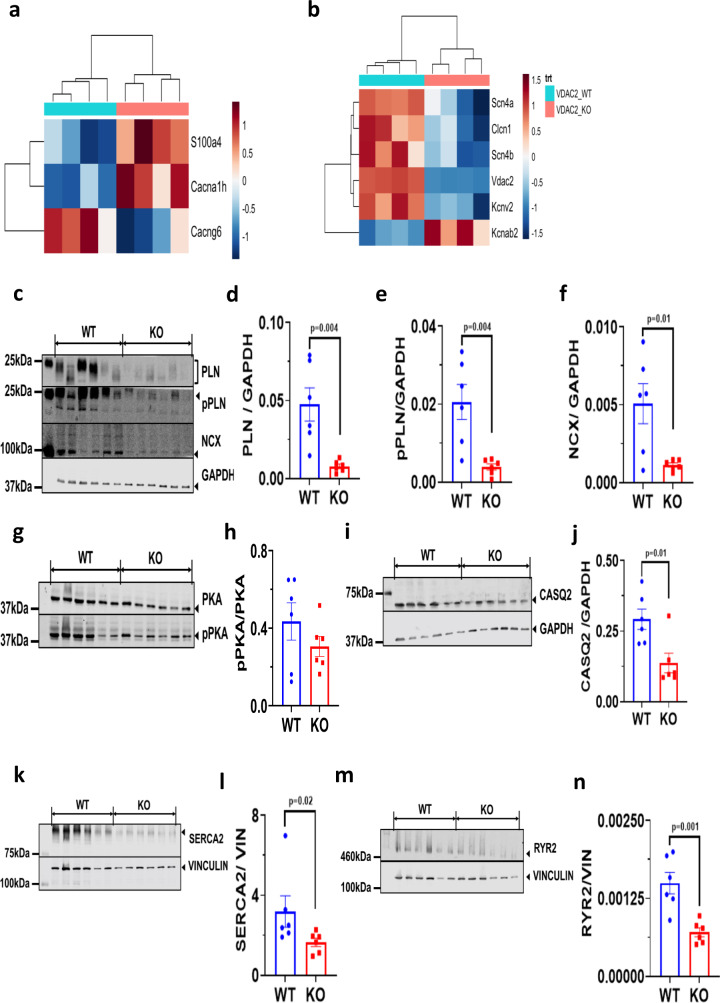
Altered gene and protein expression profiles indicate altered calcium signaling and regulation in VDAC2-KO. **a**, **b** Differential gene expression data derived from 16-week old cardiac ventricular tissue (*n* = 4); **c**–**f** Western blot image of PLN (*p* = 0.0042), pPLN (*p* = 0.0045), and NCX (*p* = 0.0121) along with lane-loading control GAPDH and respective quantification using Image Studio Lite (version 5.2.5) (*n* = 6); **g**, **h** Western blot image of PKA and pPKA and quantification using Image Studio Lite (version 5.2.5) (*p* = ns) (*n* = 6); **i**, **j** Western blot image of CASQ2 and lane-loading control GAPDH and respective quantification using Image Studio Lite (version 5.2.5) (*p* = 0.0111) (*n* = 6); **k**, **l** Western blot image of SERCA2 and lane-loading control VINCULIN and quantification using Image Studio Lite (version 5.2.5) (Mann–Whitney: *p* = 0.0260) (*n* = 6); **m**, **n** Western blot image of RYR2 and lane-loading control VINCULIN and quantification using Image Studio Lite (version 5.2.5) (*p* = 0.0018) (*n* = 6). *p*-value: unpaired two-tailed *t*-test and two-tailed Mann–Whitney test performed. Data are represented as mean ± SEM.

Gene expression profile from ventricular RNA of 16-week-old KO mice showed a number of differentially expressed genes involved in calcium signaling and regulation. Calcium channel genes such as *Cacna1h*, a T-type calcium channel (Cav 3.2) was upregulated whereas *Cacng6*, a subunit of the LTCC was significantly downregulated (Fig. 2a). Other ion channels were also differentially expressed between the two groups. Specifically, a significant downregulation of sodium and potassium ion channels was observed in VDAC2-KO mice, including *Scn4a*, *Scn4b*, and *Kcnv2*, all of which have been reported to play significant roles in normal cardiac function (Fig. 2b). Proteins involved in ECC such as RYR2, SERCA2, NCX1, total and phosphorylated phospholamban (PLN), and calsequestrin (CASQ2, the SR calcium-binding protein) were significantly decreased in the KO (Fig. 2c–n). Collectively, these results suggest that loss of VDAC2 leads to abnormalities in cellular calcium signaling pathways.

### Impaired calcium cycling in VDAC2-KO cardiac myocytes

**Fig. 3:**
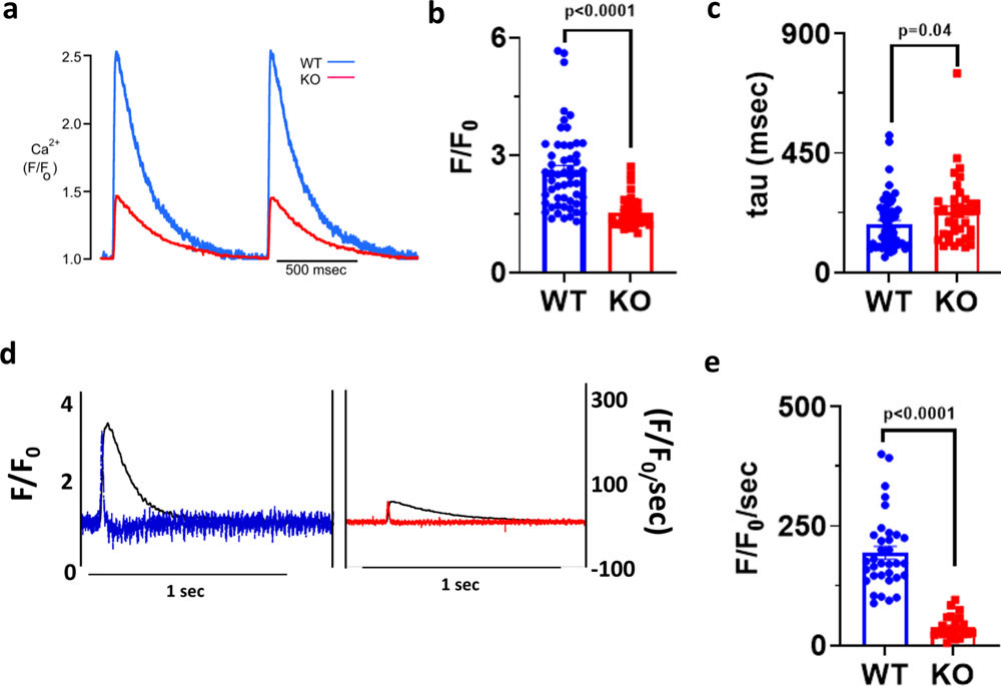
Impaired calcium cycling in VDAC2-KO cardiomyocytes. **a** Representative calcium transient image of 16-week-old mice (Time frame, 500 ms); **b** Amplitude (*F*/*F*_0_) data (*p* < 0.0001) (WT *n* = 54, KO *n* = 38, *N* = 3); **c** Rate of calcium uptake (tau) (ms) (*p* = 0.0492) (WT *n* = 54, KO *n* = 38, *N* = 3); **d** Representative image of rate of calcium release of 16-week-old mice (Time frame, 1 s); **e** Rate of calcium release (*F*/*F*_0_/s) (*p* < 0.0001) (WT *n* = 34, KO *n* = 23, *N* = 3). *p*-value: unpaired two-tailed *t*-test performed in all comparisons. Data are represented as mean ± SEM.

**Fig. 4:**
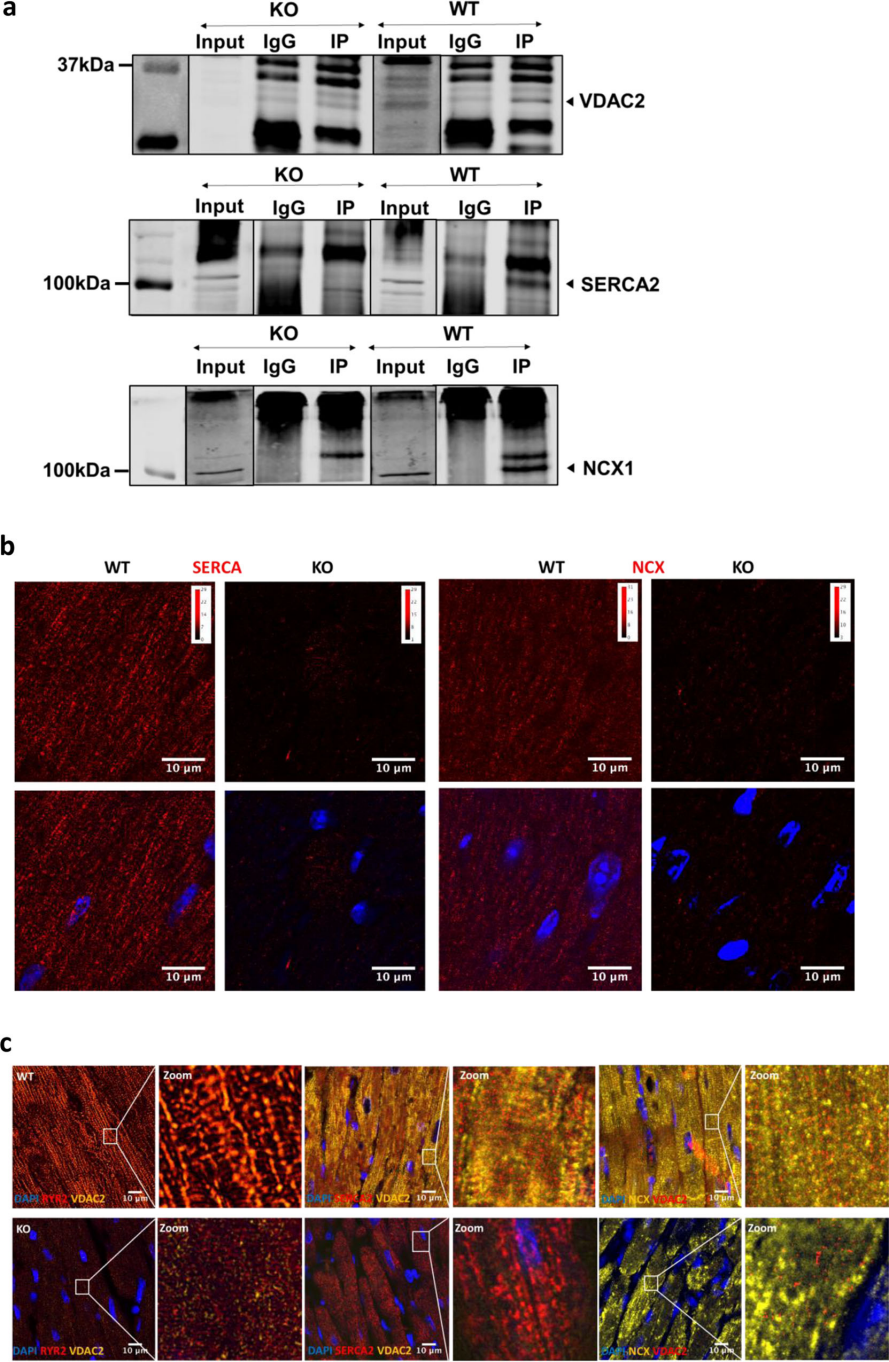
Cross-talk between mitochondria and ER/SR facilitates mitochondrial calcium cycling. **a** Representative co-immunoprecipitation image from a cardiac ventricular sample of 8-week-old mice (*n* = 3) in WT and KO (Input—non-IP sample; IgG—control antibody; IP—VDAC2 protein pull-down); **b** Representative proximity ligation assay image for VDAC2–SERCA2 and VDAC2–NCX1 interaction in 8-week-old mice (scale bar, 10 μm) (*n* = 3); **c** Representative immunofluorescence image from 16-week-old mice heart (scale bar, 10 μm) (*n* = 3).

**Fig. 5:**
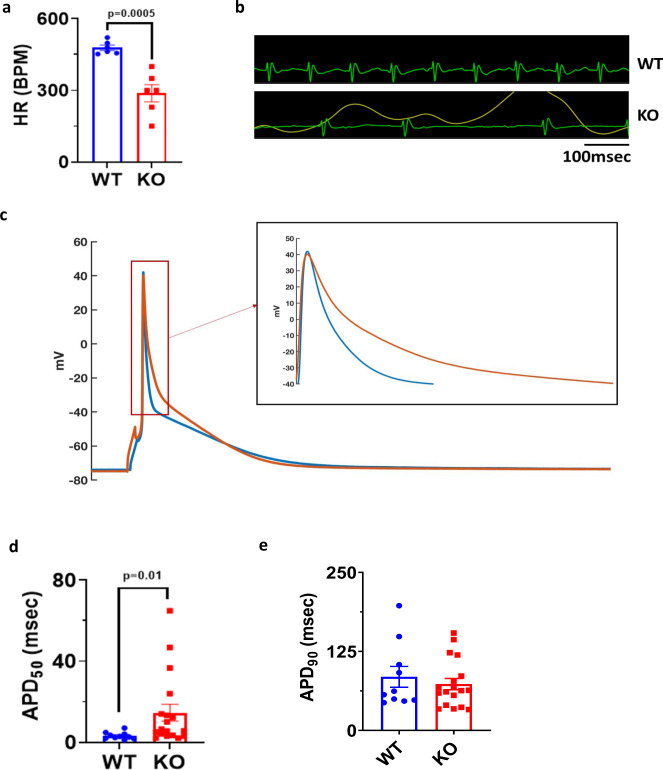
Altered action potential in VDAC2-KO cardiomyocytes. **a** Heart rate (HR), beats per minute (BPM) (*p* = 0.0005) (*n* = 6); **b** Representative ECG of 16-week-old mice (time frame, 100 ms); **c** Representative action potential image of 16-week-old mice cardiomyocyte (time frame, 100 ms); **d**, **e** Action potential repolarization duration at 50% and 90%, respectively (two-tailed Welch test: APD90 *p* = ns, APD50 *p* = 0.0148) (WT *n* = 10, KO *n* = 18, *N* = 3). *p*-value: unpaired two-tailed *t*-test and two-tailed Welch test performed. Data are represented as mean ± SEM.

To further assess calcium handling in these mice, we recorded calcium transients in 16-week-old cardiac myocytes. Compared to WT, VDAC2-KO myocytes had smaller calcium transients (*F*/*F*_0_) with a slower rate of decay (tau) and slower maximum rate of rising (*F*/*F*_0_/s) (Fig. 3a–e). To investigate if the decreased calcium transients are directly attributable to the lack of VDAC2 we tested the effects of VDAC2 agonist efsevin^14^ on calcium transients in WT cardiomyocytes and found the opposite effect, namely an increase in calcium transient amplitude and an acceleration of the decay phase (Supplementary Fig. 3a–c). SERCA2a and NCX1 are the two main channels involved in cytosolic calcium clearance during diastole and we showed that these protein levels are significantly reduced in VDAC2-KO (Fig. 2f and l). Co-immunoprecipitation (Co-IP) studies using whole-heart lysate and pull-down using VDAC2 antibody revealed VDAC2–SERCA2 interaction and VDAC2–NCX1 interaction, respectively. VDAC2-KO heart was used as a negative control for co-IP (Fig. 4a). This interaction was also confirmed by immunofluorescence and proximity ligation assays (PLA) (Fig. 4b, c). We hypothesize that these interactions, similar to the previously discovered VDAC2–RyR2 interaction^10^, are crucial for calcium uptake by the mitochondria and indirectly by the SR. As a result, a disruption of this interaction caused drastic changes in the calcium signaling in the cardiac myocytes. This altered calcium signaling may explain bradyarrhythmia observed in VDAC2-KO (Fig. 5a, b)^17^. Additionally, in agreement with the altered gene expression of the above-mentioned ion channels, the action potential (AP) of the KO was different from that of WT with a significantly longer AP duration at 50% repolarization (APD_50_) and a normal 90% repolarization (APD_90_) (Fig. 5c–e). Collectively, we found significant differences in most aspects of calcium cycling in the VDAC2-KO, which presumably contributes to the progressive decline in myocardial function.

### Altered mitochondrial structure and function in VDAC2-KO

**Fig. 6:**
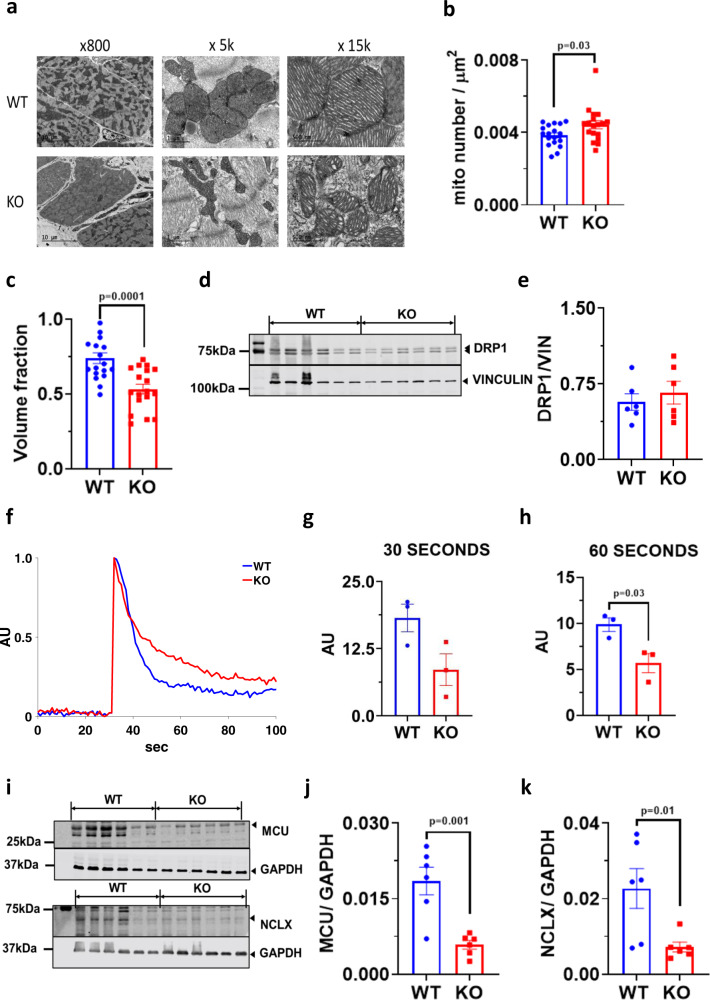
Altered mitochondrial structure and function in VDAC2-KO. **a** Representative transmission electron microscopy image at ×800, ×5000 and ×15,000 magnifications (scale bar, 10 μm, 1 μm, and 500 nm, respectively) (*n* = 3); **b** Number of mitochondria per unit area measured using Adobe Photoshop CC 2019 (*p* = 0.0365) (*n* = 18 images, *N* = 3); **c** Mitochondrial volume fraction measured using Adobe Photoshop CC 2019 using 22 × 14 grid (p = 0.0001) (*n* = 18 images, *N* = 3); **d**, **e** Western blot image of DRP1 and lane-loading control VINCULIN and quantification using Image Studio Lite (version 5.2.5) (*p* = ns) (*n* = 6); **f** Representative mitochondrial calcium uptake image produced using MATLAB (*n* = 3); **g**, **h** Mitochondrial calcium uptake in the first 30 s (*p* = ns) and 60 s of calcium pulse (*p* = 0.0300) (*n* = 3), respectively; **i**–**k** Western blot image of MCU (*p* = 0.0014) and NCLX (*p* = 0.0169) along with respective lane-loading control GAPDH and quantification using Image Studio Lite (version 5.2.5) (*n* = 6). *p*-value: unpaired two-tailed *t*-test performed in all comparisons. Data are represented as ±SEM.

Transmission electron microscopy (TEM) performed on 16-week-old KO and WT hearts showed substantial alterations in mitochondrial distribution and structure (Fig. 6a–c). KO mitochondria were significantly smaller, disoriented, and less dense compared to WT mitochondria. We did not observe any significant difference in dynamin-related protein 1 (DRP1) levels (a marker for mitochondrial fission) although it showed a trend towards an increase in KO mice (Fig. 6d, e). We also observed a significant reduction in the rate of calcium uptake in KO mitochondria suggesting VDAC2 plays an important role in mitochondrial calcium transport (Fig. 6f–h). A significant decline in MCU and NCLX protein levels was also observed in the KO (Fig. 6i–k). These proteins are known to play an important role in mitochondrial calcium transport and signaling and the decreased protein level in the KO is consistent with the slower rate of calcium uptake.

### Increased cardiac fibrosis observed in VDAC2-KO

**Fig. 7:**
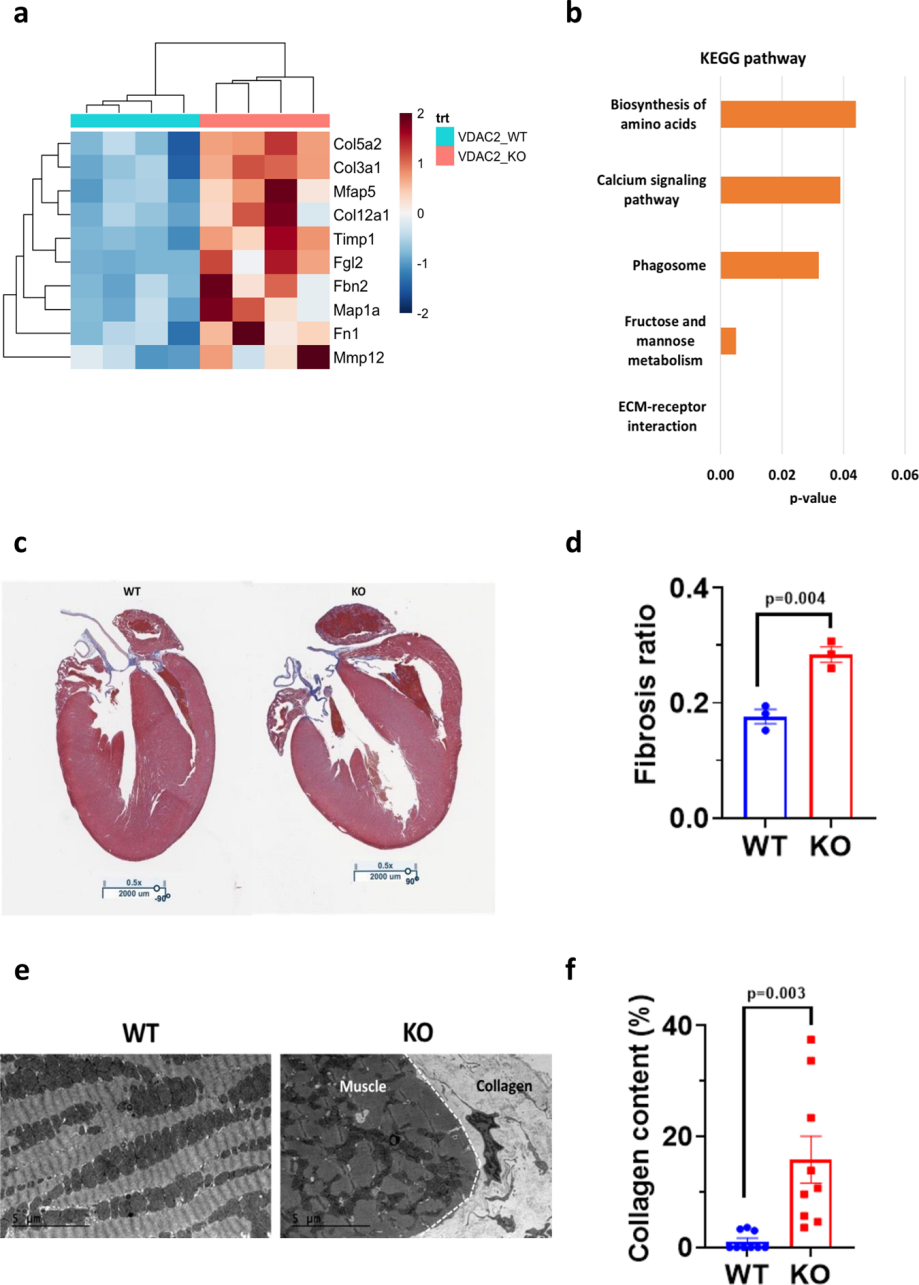
Increased cardiac fibrosis observed in VDAC2-KO. **a** Differential gene expression data from 16-week-old mice ventricular sample (*n* = 4); **b** KEGG pathway analysis of differentially expressed genes in KO indicates ECM production pathway as highest statistical significance (*n* = 4); **c** Representative Masson’s trichrome stain image (×0.5 magnification; scale bar, 2000 μm) (*n* = 3); **d** Fibrosis quantification using Aperio software and colocalization v9 algorithm (*p* = 0.0043) (*n* = 3); **e** Representative transmission electron microscopy image (scale bar, 5 μm) (*n* = 3); **f** Collagen quantification using ImageJ software (*p* = 0.0033) (*n* = 9 images, *N* = 3). *p*-value: unpaired two-tailed *t*-test performed in all comparisons. One-tail Fisher Exact test performed for KEGG pathway analysis. Data are represented as ±SEM.

Gene expression profile showed a significant upregulation of extracellular matrix (ECM) proteins and collagen encoding genes including *Col3a1, Col5a2*, *and Mmp12* in the KO that suggests increased collagen deposition and fibrosis (Fig. 7a). Kyoto Encyclopedia of Genes and Genomes (KEGG) pathway analysis in KO mice indicated that the ECM production pathway changes reached the highest statistical significance (Fig. 7b). Masson’s trichrome stain and TEM on 16-week-old mice hearts showed significantly higher collagen content in the KO (Fig. 7c–f). The observed increased myocardial fibrosis is consistent with adverse structural and functional myocardial remodeling and cardiomyopathy in the KO mice.

### Metabolic alterations observed in VDAC2-KO

Since VDAC2 is known to interact with a number of key enzymes like phosphofructokinase (PFK) and hexokinase (HK) that are involved in glycolysis and is known to transport a number of metabolites and ATP, we also measured the RNA and protein levels of specific enzymes in key metabolic pathways. The volcano plot from RNA sequencing data showed significant differences in enzymes involved in metabolism and we observed an overall downregulation of enzyme levels involved in glycolysis, tricarboxylic acid (TCA) cycle, and glycolytic accessory pathways. Specifically, enzymes including transketolase (TKT), succinyl-CoA ligase (SUCLA2), pyruvate dehydrogenase (PDH), and pyruvate carboxylase (PC). However, no significant difference was observed in fatty acid oxidation (FAO) pathways (Supplementary Fig. 4a–o). VINCULIN and GAPDH were used as lane-loading controls and we observed no difference in these protein levels between WT and KO (Supplementary Fig. 4p).

### No significant difference in mitochondrial ROS production

VDAC2-KO did not show a significant difference (compared to WT) in reactive oxygen species (ROS) production upon inhibition of complex I and complex III using rotenone and antimycin, respectively (Supplementary Fig. 5a–d). However, we found a trend (*p* = 0.08) towards a decrease in complex II in the KO (Supplementary Fig. 5e–i).

### Unaltered mitochondrial respiration

Oxidative phosphorylation (OXPHOS) measurements with LV myocardial tissue showed no difference in mitochondrial oxygen consumption between the WT and KO under normal conditions (Supplementary Fig. 6a–i). This suggests that VDAC2 is not essential for normal mitochondrial respiration despite its role in calcium signaling. These results are similar to those observed in previous publications with MCU-KO^11,18^.

### Partial rescue of HF phenotype upon VDAC2 re-introduction

**Fig. 8:**
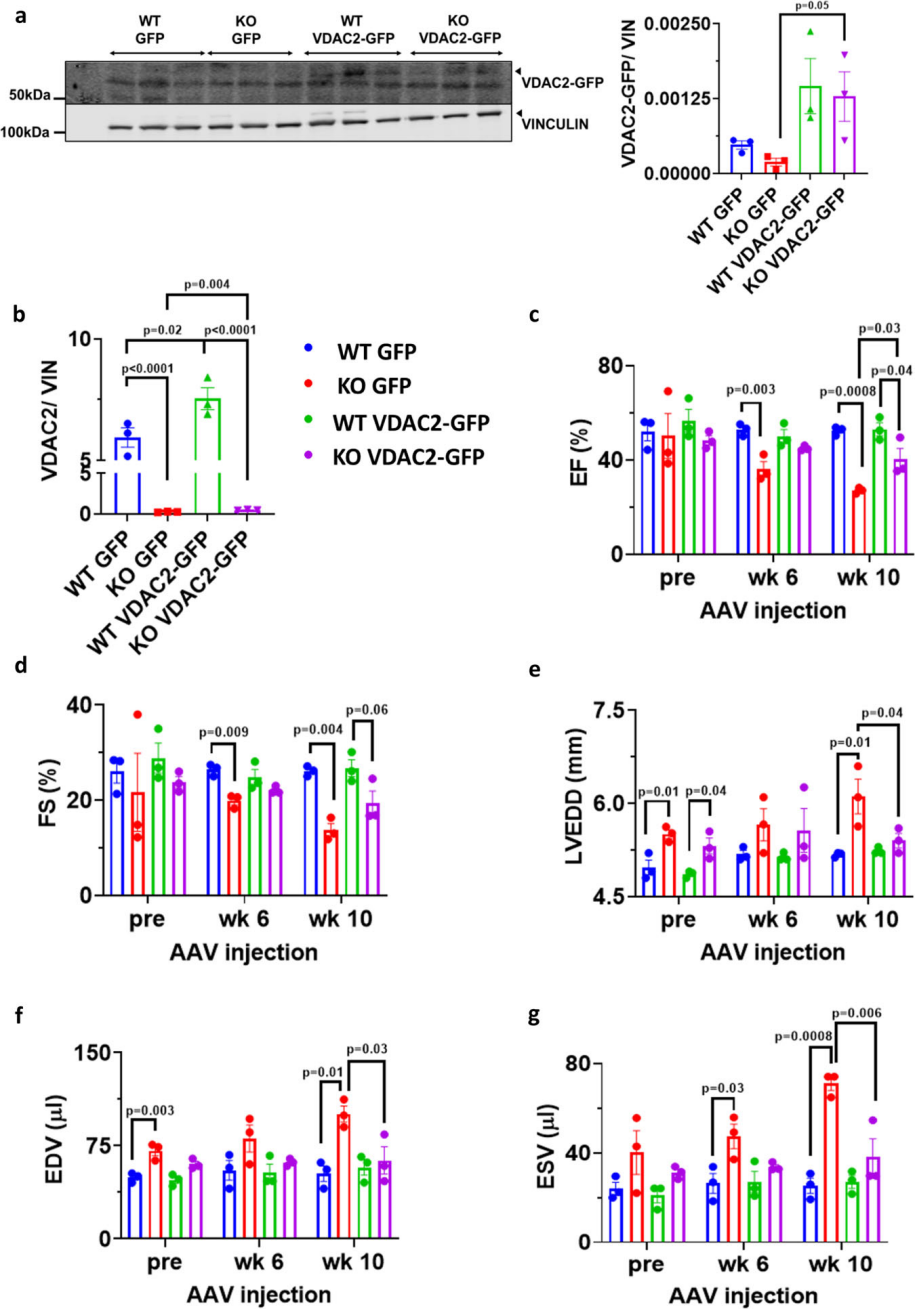
Partial rescue of HF phenotype upon VDAC2 reintroduction. **a** Western blot image of VDAC2-GFP and lane-loading control VINCULIN and quantification using Image Studio Lite (version 5.2.5) (KO GFP vs. KO VDAC2-GFP *p* = 0.0568) (*n* = 3); **b** qRT-PCR of *Vdac2* normalized to *Vcl* (Vinculin) (KO GFP vs. KO VDAC2-GFP *p* = 0.0044) (*n* = 3); **c**–**g** Serial echocardiography parameter including ejection fraction (EF) (week 6: WT GFP vs. KO GFP *p* = 0.0036, week 10: WT GFP vs. KO GFP *p* = 0.0008, WT VDAC2-GFP vs. KO VDAC2-GFP *p* = 0.0456, KO GFP vs. KO VDAC2-GFP *p* = 0.0359), fractional shortening (FS) (week 6: WT GFP vs. KO GFP *p* = 0.0091; week 10: WT GFP vs. KO GFP *p* = 0.0043, WT VDAC2-GFP vs. KO VDAC2-GFP *p* = 0.0652), left ventricular end diastolic diameter (LVEDD) (pre: WT GFP vs. KO GFP *p* = 0.0169, WT VDAC2-GFP vs. KO VDAC2-GFP *p* = 0.0407; week 10: WT GFP vs. KO GFP *p* = 0.0107, KO GFP vs. KO VDAC2-GFP *p* = 0.0444), end diastolic and end systolic volumes (EDV (pre: WT GFP vs. KO GFP *p* = 0.0032; week 10: WT GFP vs. KO GFP *p* = 0.0104, KO GFP vs. KO VDAC2-GFP *p* = 0.0378) and ESV (week6: WT GFP vs. KO GFP *p* = 0.03, week 10: WT GFP vs. KO GFP *p* = 0.0008, KO GFP vs. KO VDAC2-GFP *p* = 0.0066)) respectively (*n* = 3). *p*-value: unpaired *t*-test performed in all comparisons between KO GFP and KO VDAC2-GFP in panels **a** and **b**. Ordinary one-way ANOVA was performed for echocardiographic data and panel **b**. Data are represented as ±SEM.

Cardiac ventricular myocyte-specific reintroduction of VDAC2 in KO and WT mice was assessed using western blot and qRT-PCR (Fig. 8a, b). Serial echocardiography was performed on these mice up to 10 weeks post-injection (16-week-old mice) to get comparable results. Cardiac structural and functional improvement was observed in KO mice injected with AAV9-αMHC-VDAC2-GFP vector evident from relative improvement in EF, FS, and LV volumes and from a decrease in LV diameter compared to KO mice injected with control AAV9-αMHC-GFP vector (Fig. 8c–g). Complete rescue of the phenotype was not observed, however, the re-introduction of VDAC2 aided in better cardiac functioning.

### VDAC2 as a pharmacological target for HF

**Fig. 9:**
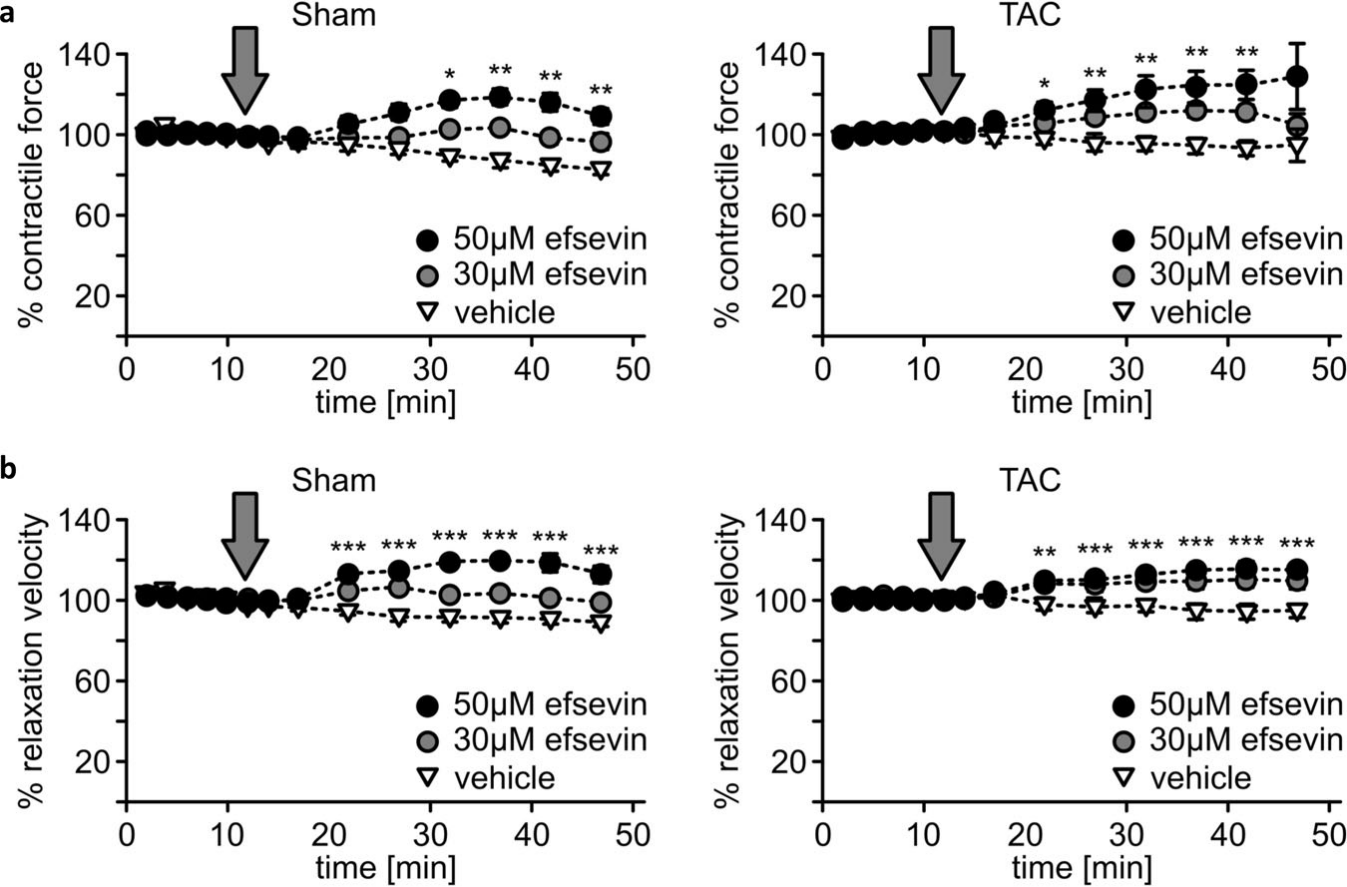
Enhanced contractile force and accelerated relaxation speed in myocardial tissue upon VDAC2 activation. **a** Measurement of relative contractile force in organotypic myocardial slices from both Sham- (left panel) and TAC-operated mice (right panel) after superfusion with either 30 or 50 µM of the VDAC2 agonist efsevin. Each dot represents the quantification of twitch amplitudes over 20 s at indicated time points. The gray arrow indicates the start of efsevin superfusion (left panel: DMSO vs. 30 μM Efsevin: 10 min *p* = 0.016, 32 min *p* = 0.010, 37 min *p* = 0.0004, 42 min *p* = 0.0012 and 47 min *p* = 0.0062; DMSO vs. 50 μM Efsevin: 22 min *p* = 0.0007, 27–47 min *p* < 0.0001, respectively) (right panel: DMSO vs. 30 μM Efsevin: 17 min *p* = 0.0038, 22 min *p* = 0.0009, 27 min *p* = 0.00031, 32 min *p* = 0.00029, 37 min *p* = 0.00015, 42 min *p* = 0.00073; DMSO vs. 50 μM Efsevin: 22 min *p* = 0.0218, 27 min *p* = 0.0044, 32 min *p* = 0.0037, 37 min *p* = 0.0031, 42 min *p* = 0.0034). **b** Relaxation speed of twitches of myocardial slices shown in a (*N* = 8, *n* = 22 slices for TAC, *N* = 6, *n* = 14 slices for Sham) (left panel: DMSO vs. 30 μM Efsevin: 22 min *p* = 0.0072, 27 min *p* = 0.00019, 32 min *p* = 0.044, 37 min *p* = 0.0125, 42 min *p* = 0.0145, 47 min *p* = 0.0308; DMSO vs. 50 μM Efsevin: 22–47 min *p* < 0.0001 respectively) (right panel: DMSO vs. 30 μM Efsevin: 22 min *p* < 0.0001, 27 min *p* = 0.0001, 32 min *p* < 0.0001, 37 min *p* < 0.0001, 42 min *p* = 0.0001, 47 min *p* = 0.0002; DMSO vs. 50 μM Efsevin: 22 min *p* = 0.002, 27 min *p* = 0.0001, 32–42 min *p* < 0.0001, respectively, 47 min *p* = 0.0001). *p*-value: unpaired *t*-test performed in all comparisons. *<0.05, **<0.01 and ***<0.001. Data are represented as ±SEM.

We show that VDAC2 regulates cardiac calcium signaling without having major effects on bioenergetics and VDAC2 deletion causes DCM, making it a candidate structure for HF therapy. We therefore, addressed the question of increasing VDAC2s calcium uptake activity could enhance cardiac contractile force in HF. Therefore, we used the established model of pressure overload-induced HF by transverse aortic constriction (TAC). Since the administration of efsevin to mice is difficult for more than a few days due to its short plasma half-life time and efsevin is not orally bioavailable, we took advantage of organotypic heart slices and investigated the effect of efsevin on myocardial contractile strength. Strikingly, efsevin enhanced contractile strength and accelerated relaxation in both control and failing hearts (Fig. 9a, b).

## Discussion

In this study, we investigated the role of VDAC2 in the heart by creating a cardiac ventricular myocyte-specific deletion of VDAC2 using α-Myosin heavy chain (αMHC) promoter. We observed increased postnatal mortality along with severe impairment in cardiac structure and function consistent with DCM. Our gene and protein studies suggested altered calcium signaling in the KO mice. In agreement with these data, the KO showed smaller calcium transients, a slower decay time, and a slower rate of calcium release. Substantial adverse cardiac remodeling and mitochondrial disorganization along with slower mitochondrial calcium uptake were also observed in the KO indicating that VDAC2 plays a crucial role in cardiac functioning by maintaining mitochondrial and cellular calcium homeostasis. Additionally, reintroduction of VDAC2 in young-KO mice showed a partial rescue of the cardiomyopathy phenotype suggesting the importance of VDAC2 in normal cardiac functioning.

Mitochondria–ER/SR interactions have long been studied and it is commonly believed that this interaction is crucial for mitochondrial calcium dynamics^9,19,20^. Mitochondria are known to recognize calcium-rich niches near SERCA2, RYR2, and NCX1 where major calcium transport happens^21^. We show in this study that the crosstalk of VDAC2 with both calcium release through—RYR2 and diastolic calcium removal by SERCA2 and NCX1 plays an important role in determining cellular calcium homeostasis and when this crosstalk is lost, there is a significant impairment in both mitochondrial and cytosolic calcium signaling. We also observed a significant reduction in most calcium handling proteins like RYR2, SERCA2, NCX1, and CASQ2 in VDAC2-KO mice. While these results are unexpected, they might be explained by different hypotheses: It is feasible that the lack of mitochondrial calcium uptake specifically through the loss of RYR2–VDAC2 connection results in a lack of energy demand which was shown to depend on mitochondrial calcium uptake^22^. In this scenario the basic metabolism of VDAC2-KO cells would be comparable to WT (as demonstrated in our results), while they are unable to adapt to higher workload under stress situations and might consecutively down-regulate calcium handling proteins. Alternatively, the down-regulation might be a result of compensatory mechanism to the slower rate of calcium uptake observed in the KO. Additionally, a down-regulation due to loss of structural integrity of the myocardium as observed in our experiment is feasible. Also, our data stands in contrast to previous observations that knock-down of VDAC2 in HL-1cells did not alter expression levels of calcium handling proteins^10^. However, major differences between the model systems might explain these discrepancies: Partial inhibition of VDAC2 (~75%) was achieved using viral transduction and cells were cultured in vitro for limited time. It is thus feasible that the down-regulation of these proteins is masked either by the remaining VDAC2 expression, the lack of triggers present in vivo-like pressure or adrenergic stimulation, or that these translational changes only manifest after a longer time.

The significant impairment in SR-mediated calcium release and uptake in VDAC2-KO mice can be associated with reduced RYR2 and SERCA2 activities, respectively. We also observed a significant downregulation of total and phosphorylated PLN in VDAC2-KO which may account for the reduced SERCA2 activity and slower rate of decline in calcium transient. Additionally, the varying molecular weights seen in total PLN protein is likely due to post-translational modification of PLN by phosphorylation at different sites or different combination of sites including Ser10, Ser16, and/or Thr17. Therefore, an average intensity of the bands was taken and normalized with GAPDH^23–25^. Our co-IP studies suggest an interaction of VDAC2 with SERCA2. We propose that this interaction might be important for facilitating mitochondrial calcium uptake and also indirectly influencing cytosolic calcium signaling. Another pathway for calcium extrusion from the cytoplasm is through NCX1 and we observed that VDAC2 interacts with NCX1. This interaction, similar to that with SERCA2 and RyR2, is also likely to influence cellular calcium signaling. Sub-cellular regions near RyR2, SERCA2, and NCX1 act as calcium compartments with markedly different calcium concentration than the bulk cytosol and both the interaction of VDAC2 with calcium handling proteins and the localization of mitochondria near these regions indicates a substantial role of mitochondrial calcium uptake for cellular calcium handling. We propose that upon disruption of this interaction, there is less overall mitochondrial calcium uptake and reduced cytosolic calcium clearance which leads to impaired intracellular calcium signaling (Supplementary Fig. 7).

In agreement with other studies, we showed that VDAC2 is crucial for cellular calcium cycling and normal cardiac functioning, thereby making it a promising therapeutic target for DCM and chronic HF^6,15^. To test this hypothesis, we investigated the potency of efsevin as a therapeutic agent for the treatment of HF. Indeed, efsevin enhanced contractile force in organotypic tissue slices of failing murine myocardium, establishing VDAC2 as a promising target for HF.

HF is associated with an increased risk for arrhythmia and efsevin was previously suggested to suppress arrhythmogenesis in cardiomyocytes^13,14^ suggesting a role for VDAC2 also as a protective pathway against arrhythmia. This data is backed up by our findings, that VDAC2-KO mice also experienced arrhythmias. We hypothesize that the altered calcium signaling (reduced CASQ2, RYR2, and SERCA2 activities) has the potential to cause these arrhythmias^26,27^. The reduced CASQ2 activity suggests reduced RyR2 sensitivity to cytosolic calcium which in turn explains the reduced gene expression of L-type calcium channel^28^. Additionally, the expression of T-type calcium channels in the adult KO myocardium is indicative of disease states such as cardiomyopathies and HF where increased expression of T-type channels in adult ventricular myocytes leads to increased susceptibility to arrhythmias^29,30^. The cellular patch-clamp experiments in the VDAC2-KO showed a tendency towards increased early after depolarizations perhaps due to reactivation of these calcium channels^31^. We also observed a significantly longer APD_50_ in the KO which can be explained by the genetic downregulation of the transient outward channel thereby making VDAC2 influence on the cardiomyocyte even more prominent.

VDAC2–tubulin interaction and VDAC2–PFKP interaction have been studied in cancer metabolism research were specifically targeting VDAC2 has shown to down-regulate glycolysis thereby preventing cancer cell growth and proliferation^32,33^. In agreement with this work, we also observed significant alterations in a number of enzymes involved in glucose metabolism, its accessory pathways, and the TCA cycle but no difference was observed in FAO or electron transport chain (ETC). This unaltered FAO and ETC may help explain the relatively unaltered mitochondrial respiration since these metabolic pathways provide the necessary ATP.

VDAC2 plays an important role in apoptosis by interacting with BAK, a proapoptotic protein, and keeping it inactive^34^. Essentially, VDAC2–BAK interaction prevents cellular apoptosis, and *Vdac2* deletion results in free (or active) BAK thus causing excessive apoptosis^35^. We hypothesize that this increased apoptosis during late cardiac development might contribute to the increased fibrosis observed in our VDAC2-KO mice.

Calcium release into the cytoplasm is mainly mediated by the RyR2 and previous publications have established the importance of physical interaction between VDAC2 and RyR2 for normal calcium transport into the mitochondria^10^. Mitochondrial calcium uptake was significantly impaired in our VDAC2-KO mice indicating the importance of VDAC2 in mitochondrial calcium transport. A similar reduction in mitochondrial calcium uptake was observed in MCU-KO mice, however, no other phenotypical difference was observed between the MCU-WT and MCU-KO^36^, indicating differences in the role of these two transporters incellular calcium handling. One explanation derived from this work is that VDAC might act as a scaffolding protein to link the MCU to other calcium handling proteins and thus creating cellular calcium microdomains, while MCU might serve as the controller of mitochondrial calcium uptake. This is in line with observations that VDAC2 was described to be a vividly interacting channel^20^.

Overall, our findings highlight the importance of VDAC2 in influencing ECC and cardiac disease progression. Previous research has shown that increased calcium uptake through VDAC2 (using efsevin) reverses arrhythmic phenotypes. Here, we demonstrate the crucial role of VDAC2 in ECC and the pathogenesis of DCM and provide reasonable evidence for VDAC2 to serve as a therapeutic target for HF. Thus, drugs targeting VDAC2 might fulfill a double role by suppressing cardiac arrhythmia and enhancing cardiac function.

## Methods

### Animals and animal care

All animal studies were performed in accordance with the University of Iowa Animal Care and Use Committee (IACUC). All procedures involving animals were approved by the Animal Care and Use Committee of the University of Utah and complied with the American Physiological Society’s *Guiding Principles in the Care and Use of Animals* and the UK Animals (Scientific Procedures) Act 1986 guidelines. The mice were housed in 12 h dark/light cycle at 70 °F and 40% humidity.

### Generation of cardiac-specific knockout

C57BL6J mice were used for all experiments. Both male and female mice were used for all experiments. *Vdac2* flox/flox mice were obtained from Ren et al. ^35^. The founder mice were cross-bred with transgenic αMHC-Cre (Jackson Laboratory#009074)^37^ and the resulting heterozygous (*Vdac2* flox/+; αMHC-Cre) mice were crossed to obtain a cardiac ventricular myocyte-specific deletion of VDAC2.

### Echocardiographic analysis

Mice were anesthetized with 1.5% Isoflurane (Vet One, NDC13985-046-60) during echocardiography. Echocardiographic images were taken on the Vivo system. Echoes were performed serially from embryonic age 17 to 16 weeks post-natal. 2D long-axis and short-axis views were obtained and used for analysis using Vivo strain software (version 3.1.1). Two consecutive cardiac cycles were used for all the measurements. Limb leads were used to record an electrocardiogram (ECG).

### Collagen content evaluation/Masson’s trichrome stain

Mice were euthanized using sodium pentobarbital (NDC76478-501-50). Hearts were harvested and formalin-fixed for 48 h followed by paraffin embedding. 5 μm thick sections were cut and stained with Trichrome for fibrosis analysis using the Dako automated special strainer. The slides for fibrosis analysis were scanned under ×20 and analyzed using Aperio Image Scope software (version 12.3.2.8013) (using the colocalization v9 algorithm)^38^. A ratio of the total stained area to the collagen-stained area was reported.

### Cardiomyocyte isolation

Adult mice were anesthetized with sodium pentobarbital (50 mg kg^−1^) and the excised heart was attached to an aortic cannula and perfused with solutions gassed with 100% O_2_ and held at 37 °C, pH 7.3. Perfusion with a 0 mM Ca^2+^ solution for 5 min was followed by 15 min of perfusion with the same solution containing 1 mg ml^−1^ collagenase (type II, Worthington Biochemical, Freehold, NJ, USA) and 0.1 mg/ml protease (type XIV, Sigma Chemical, catalog #P5147). The heart was then perfused for 1 min with a stopping solution (the same solution containing 20% serum and 0.2 mM CaCl_2_). All perfusions were performed at a flow rate of 2 ml min^−1^. The atria were removed and the ventricles were minced and shaken for 10 min, and then filtered through a nylon mesh. Cells were stored at 37 °C in a normal HEPES buffered solution. All myocytes used in this study were rod-shaped, had well-defined striations, and did not spontaneously contract. Experiments were performed within 7 h of isolation.

### Cell super-fusion chamber

The Plexiglas cell bath had a clear glass bottom and was mounted on the stage of an inverted microscope (Diaphot, Nikon, Japan). The temperature of the solutions in the super-fusion chamber was 36 ± 0.3 °C. Bathing solutions flowed continuously through the bath at 4 ml min^−1^, and solution depth was held at ~2–3 mm. Exchange of the bath solution required ~5 s. The bottom of the bath was coated with laminin (Collaborative Research, Bedford, MA, USA) to improve cell adhesion.

### Cell bathing solutions and pipette filling solutions

The normal control bathing solution contained (mM): 126.0 NaCl, 11.0 dextrose, 4.4 KCl, 1.0 MgCl_2_, 1.08 CaCl_2_, and 24.0 HEPES titrated to pH 7.4 with 1 M NaOH. The pipette solution used for recording APs contained (mM): 110.0 KCl, 5.0 NaCl, 5.0 MgATP, 5.0 phosphocreatine, 1.0 NaGTP, 10.0 HEPES titrated to pH 7.2 with 1 M KOH.

### AP measurement

Transmembrane potential (*V*_m_) was measured with borosilicate glass suction pipettes (resistance 1–2 MΩ when filled) connected to an Axoclamp 2B amplifier system (Axon Instruments/Molecular Devices) in bridge mode^39^. The *V*_m_ signal was filtered at 5 kHz, digitized at 50 kHz with a 16-bit A/D converter (Digidata 1322A), and analyzed using PCLAMP 8 software (Molecular Devices). APs were triggered with brief (2–3 ms) square pulses of depolarizing intracellular current (~2 nA). APD_50_ and APD_90_ were measured.

### Intracellular calcium concentration measurement

Calcium transients were detected in single myocytes with an epifluorescence system using the fluorescent indicator, Fluo-4. Cells were incubated in the normal control solution containing 12.5 µM Fluo4-AM (Invitrogen, catalog#F14201) and 0.3 mM probenecid (Sigma, catalog #P8761) at 30 °C for 20 min. The cells were then continuously bathed in the same solution containing no indicator. Probenecid (0.3 mM) was included in the bathing solutions to help retard fluo-4 loss from the cells. Fluorescence emission (535 nm, bandpass filter) was collected with a photomultiplier tube via a ×40 objective during continuous excitation at 485 nm. Calcium transients were elicited with field stimulation at a cycle length of 1 s.

### TEM and analysis

LV transmural samples from 16-week-old mice were used for this study. Tissue samples were fixed overnight at 4 °C in 0.1 M sodium cacodylate buffer containing 1% paraformaldehyde and 2.5% glutaraldehyde. Samples were washed with the same buffer and post-fixed for 2 h in 2% osmium tetroxide buffered with cacodylate buffer. Samples were then rinsed in nano-pure water and stained with en-bloc stain for 1 h at room temperature in uranyl acetate. Tissues were then dehydrated through a graded series of ethanol: 10 min in 50%, 3 × 10 min in 70%, 2 × 10 min in 95%, 4 × 10 min in absolute ethanol, and 3 × 10 min with absolute acetone. Samples were incubated at room temperature in a gradually increasing concentration of epoxy resin (Electron Microscopy Science, Hatfield, PA) to facilitate infiltration. Samples were then transferred to 50% resin in acetone for 1 h and overnight in 75% resin in acetone. Samples were then transferred to 100% resin for 8 h with three fresh resin changes. Samples were then embedded and polymerized at 60 °C for 48 h. Ultrathin sections (70 nm) were obtained with a diamond knife (Diatome) using Leica UC6 (Leica Microsystems, Vienna, Austria). Sections were post-stained with saturated uranyl acetate for 10 min followed by Reinold stain for 5 min. Sections were imaged at 120 kV with JEOL 1400 Plus^40^. Mitochondrial density and number analysis were performed using Adobe Photoshop tool using ×1500 images. A 22 × 14 grid was used for density measurements. The same images were used for collagen quantification using Fiji software. The ratio of collagen area to total area was reported.

### Mitochondria isolation and calcium uptake

Mice were euthanized using carbondioxide for 3 min and the heart was excised. The atria were removed and the ventricles were used for mitochondria isolation using differential centrifugation^41^. Protein estimation was performed using Pierce Assay. 100 μg of protein was used per trial, resuspended in 100 µL of a solution containing (in mM): 125.0 KCl, 20.0 HEPES, 10.0 K_2_HPO_4_, 5.0 Glutamate, 5.0 Malate, and 1 μM Oregon Green BAPTA/6F (pH 7.2 with 1 M KOH). Imaging was performed on a Biotek Cytation 5 instrument in 96-well flat-bottomed plates. Baseline measurements were taken for 30 s and 12 μM calcium chloride was injected and the fluorescence (excitation 490 nm, emission 517 nm) was measured for 3 min to obtain the calcium uptake slope.

### Retro-orbital injections

6-week-old WT and KO mice were randomly chosen to receive either AAV9-αMHC-VDAC2-GFP vector or control AAV9-αMHC-GFP vector. *N* = 3 mice were used in each group and a total of four groups were used (WT-AAV and KO-AAV received the vector with VDAC2 and WT-GFP and KO-GFP received control vector). A concentration of 3.1 × 10e12 VG kg^−1^ was used for each injection and the mice were serially echoed for 10 weeks post-injection.

### RNA extraction, sequencing, and qRT-PCR

miRNeasy Mini kit (Qiagen) was used for RNA extraction from the LV transmural sample. The extracted RNA was used for total RNA sequencing (RNA Seq). Agilent RNA Screen Tape Assay was used for QC experiments. Illumina TruSeq Stranded RNA kit was used for library preparation and Ribo-Zero Gold was used to remove rRNA and the sequencing was performed on an Illumina HiSeq 2500 with 50 bp single-end reads. The same RNA was used for cDNA synthesis (NEB #E3010S) and qRT-PCR was performed using *Vdac2* and *Vcl* (VINCULIN) primers (Table 1).

**Table 1:** List of antibodies used.

Resource	Concentration	Identifier	Source
*Western blot-antibodies*			
Voltage-dependent anion-selective channel protein 2 (VDAC2)	1:1000	9412S	Cell Signaling Technology
Voltage-dependent anion-selective channel protein 1 (VDAC1)	1:1000	ab14734	Abcam
Voltage-dependent anion-selective channel protein 3 (VDAC3)	1:1000	ab130561	Abcam
Vinculin	1:1000	13901S	Cell Signaling Technology
Glyceraldehyde 3-phosphate dehydrogenase (GAPDH)	1:1000	5174S	Cell Signaling Technology
Transketolase (TKT)	1:500	8616S	Cell Signaling Technology
Methylenetetrahydrofolate dehydrogenase 1 (MTHFD1)	1:500	sc-271412	Santa Cruz Biotech
Aldolase B (ALDOB)	1:200	sc-365449	Santa Cruz Biotech
Serine hydroxymethyltransferase 1 (SHMT1)	1:500	80715	Cell Signaling Technology
Isocitrate dehydrogenase (IDH1/2)	1:500	sc-373816	Santa Cruz Biotech
Phosphofructokinase 1 (PFK1)	1:500	sc-166722	Santa Cruz Biotech
Pyruvate carboxylase (PC)	1:1000	NBP1-49536	Novus Bio
Pyruvate dehydrogenase (PDH)	1:500	3205S	Cell Signaling Technology
Lactate dehydrogenase (LDH)	1:1000	3582S	Cell Signaling Technology
Hexokinase 1 (HK1)	1:500	2024S	Cell Signaling Technology
Calsequestrin 2 (CASQ2)	1:500	ab3516	Abcam
Citrate synthase (CS)	1:1000	14309S	Cell Signaling Technology
Succinyl Co-A synthetase (SUCLG2)	1:1000	8071S	Cell Signaling Technology
Succinate dehydrogenase A (SDHA)	1:1000	5839S	Cell Signaling Technology
Sodium–calcium exchanger 1 (NCX1)	1:500	ab151608	Abcam
Sarcoplasmic endoplasmic reticular calcium ATPase 2 (SERCA2)	1:500	4388S	Cell Signaling Technology
Ryanodine receptor 2 (RYR2)	1:500	MA3-916	Thermofisher Scientific
Phospholamban (PLN)	1:1000	14562S	Cell Signaling Technology
Phospho-phospholamban (pPLN)	1:1000	8496S	Cell Signaling Technology
Protein kinase A (PKA)	1:500	SAB4502337	Sigma Aldrich
Phospho-protein kinase A (pPKA)	1:500	SAB4503969	Sigma Aldrich
Mitochondrial calcium uniporter (MCU)	1:500	14997S	Cell Signaling Technology
Mitochondrial sodium calcium exchanger (NCLX)	1:500	SAB2102181	Sigma Aldrich
Dynamin-related protein 1 (DRP1)	1:500	14647S	Cell Signaling Technology
Oxidative phosphorylation (OXPHOS)	1:1000	ab110413	Abcam
IRDye 680RD Donkey anti-Mouse Secondary	1:10,000	926-68072	LI-COR Biosciences
IRDye 800CW Donkey anti-Rabbit Secondary	1:10,000	926-32212	LI-COR Biosciences
*Co-immunoprecipitation-antibodies*			
Voltage-dependent anion-selective channel protein 2 (VDAC2)	5 μg	PA5-28106	Thermofisher Scientific
Immunoglobulin G (IgG)	5 μg	AC005	AbClonal
*Immunofluorescence/proximity ligation assay—antibodies/stain*			
4′,6-diamidino-2-phenylindole (DAPI)	1:1000	D3571	Thermofisher Scientific
Voltage-dependent anion-selective channel protein 2 (VDAC2)	1:1000	66388-1-IG	Thermofisher Scientific
Sodium–calcium exchanger 1 (NCX1)	1:500	ab151608	Abcam
Sarcoplasmic endoplasmic reticular calcium ATPase 2 (SERCA2)	1:500	4388S	Cell Signaling Technology
Ryanodine receptor 2 (RYR2)	1:500	MA3-916	Thermofisher Scientific
*Quantitative reverse transcription-PCR-primers*			
Voltage-dependent anion-selective channel protein 2 (VDAC2)		Mm00834279_m1	Thermofisher Scientific
Vinculin		Mm00447745_m1	Thermofisher Scientific

### RNA Seq analysis

RNA Seq analysis was performed with the High-Throughput Genomics and Bioinformatics Analysis Shared Resource at Huntsman Cancer Institute at the University of Utah. mm10, M_musculus_Dec_2011, GRCm38 genome build was used for sequence alignment. Sample outliers were checked by summarizing the output files from cutadapt, FastQC, Picard CollectRnaSeqMetrics, STAR, and feature counts by using the previously described method^42^. 5% false discovery rate (FDR) with DeSeq2 (version 1.24.0) was used to identify differentially expressed genes^43^. Genes were filtered using the following criteria: adjusted *p*-value < 0.05, absolute log2 fold change > 0.585 and normalized base mean >30^43^. Heat maps were produced using the R library p heatmap. KEGG^44^ and Ingenuity pathway analysis (IPA) software was used for gene ontology analysis.

### Protein extraction and Western blotting

WT and KO mice hearts (*n* = 6 each) were excised. 30 μg of transmural LV sample was homogenized using metal beads for 3 min in RIPA buffer with 2× protease and phosphatase inhibitor (Thermo-Scientific #1861281). The homogenate was transferred to a new tube containing 10 μl of 1× PMSF and allowed to rotate for 30 min at 4 °C and centrifuged at 11,000 × *g* for 10 min at 4 °C. The supernatant was used for protein estimation using the Pierce BCA Protein Assay kit (Thermo-Scientific #23225). An equal volume of 2× Laemmli buffer (with 10% DTT) was added to the sample and boiled for 10 min at 98 °C.

30 μg protein was used for SDS–PAGE. The gel was run at constant volts (50 V) and proteins were transferred to nitrocellulose membrane at constant current (350 mA). For RYR2, proteins were transferred at constant volt (40 V) overnight. The membranes were blocked for 1 h with 5% non-fat milk and probed with primary antibodies overnight in the cold room. Antibodies and the concentration used are listed in Table 1. Blots were washed with 1× PBST thrice and probed with a secondary antibody for 1 h. Blots were washed with 1× PBST thrice prior to scanning using LI-COR. Image Studio Lite software was used to analyze the protein blots. Each blot had its own lane loading control and the values were normalized accordingly.

### Co-immunoprecipitation

30 μg of LV transmural sample from 8-week-old WT and KO mice were used for protein extraction and quantification was done using a BCA assay kit. Protein A magnetic beads (Bio-Rad, catalog#161-4011) were washed thrice with 1× PBST. 4 mg ml^−1^ of lysate was precleared by rotating with beads for 30 min at 4 °C and 10% of the precleared lysate was saved as input. 5 μg of antibody (Table 1) was used for conjugation with the beads by rotating at room temperature for 10 min, followed by washing twice with 1× PBST and once with the lysis buffer (Cell Signaling, catalog #9806). The precleared lysate and antibody were kept for rotation at room temperature for 1 h. The magnet was then washed thrice with 1× PBST, magnetized and the elute was boiled at 70 °C for 10 min with 40 μl of 1× Laemmli buffer (10% β-mercaptoethanol) and used for western blot.

### Immunofluorescence

16-week-old mice hearts were fixed in 10% paraformaldehyde for 4–6 h followed by paraffin embedding. Samples were cut at 5 μm thickness and deparaffinized and antigen retrieval was performed. The antibodies used are listed in Table 1. Secondary anti-mouse, anti-rabbit, or anti-goat were used appropriately. All images for each protein were acquired using the same laser setting and processed using Fiji software.

### Proximity ligation assay

16-week-old mice heart samples embedded in paraffin were used for this assay. Duolink In Situ PLA probe anti-mouse, anti-rabbit, and anti-goat were used (DUO92001, DUO92005, and DUO92015). The antibodies used are listed in Table 1. Samples were processed using the manufacturer’s protocol. All images for each combination were acquired using the same laser settings and processed using Fiji software.

### Force measurements in murine organotypic slices

Hearts from adult mice were excised after cervical dislocation and flushed in ice-cold preparation buffer (in mM): 136.0 NaCl, 5.4 KCl, 1.0 MgHPO_4_, 0.9 CaCl_2_, 30.0 2,3-Butanedionemonoxim, 5.0 HEPES, pH 7.4. For sectioning, hearts were embedded in low melting agarose and cut into 300 µm-thick coronary plane slices using a LeicaVT1200S vibratome and glued (Histoacryl, B. Brain Melsungen AG, Germany) onto small plastic triangles cut from 0.1 mm-thick polyester copier clear before being mounted in an organ bath chamber (Mayflower, Hugo Sachs Elektronic; HSE, Germany). Contractility was measured under a constant flow-through (4 ml min^−1^) in prewarmed measuring buffer (in mM): 136.0 NaCl, 5.4 KCl, 1.0 MgCl_2_, 0.33 NaH_2_PO_4_, 10.0 Glucose, 1.8 CaCl_2_, 23.0 NaHCO_3_, pH 7.4, 37 °C under electrical pacing at 3 Hz and force was recorded by a force transducer connected to an amplifier (Hugo Sachs, Germany) using WinEDR (University of Strathclyde, UK). Kinetics of single twitches was analyzed using LabChart Reader (ADInstruments).

### TAC surgery

TAC^45^ was performed on RyR2^R4496C+/−^ knock-in mice^46^, which were previously described to develop a severe form of HF^47^. In brief, 8–14-week-old mice were anesthetized by medetomidine/midazolam/fentanyl (0.5, 5.0, and 0.05 mg kg^−1^ body weight intraperitoneally, respectively) and the aorta was constricted around a 27-gauge needle. Sham mice underwent the same procedure without aortic constriction. 6 weeks after surgery, mice were sacrificed by cervical dislocation, hearts were isolated and flushed (in mM): 136.0 NaCl, 5.4 KCl, 1.0 MgCl_2_, 0.33 NaH_2_PO_4_, 10.0 Glucose, 0.9 CaCl_2_, 30.0 2,3-butanedionemonooxime, 5.0 HEPES, pH 7.4, 4 °C and success of TAC surgery was confirmed by measuring the heart weight to tibia length ratio.

### Statistics and reproducibility

All data were summarized as ±SEM. GraphPad Prism (version8.2.1) was used for all statistical analyses. A two-tailed test was performed in all comparisons. Unpaired Student’s *t*-test was used for all analyses with WT and KO. Multiple *t*-test and one-way ANOVA were used for longitudinal echocardiographic data and while comparing multiple groups, respectively. *p*-value < 0.05 was considered statistically significant. All experiments were repeated independently (at least three biological replicates, and three technical replicates wherever applicable) with reproducible results. Samples used for western blot quantification were from the same gel and when unavoidable, were processed in parallel.

### Reporting summary

Further information on research design is available in the Nature Research Reporting Summary linked to this article.

## Supplementary information

Supplementary Information

Peer Review File

Reporting Summary

## Data Availability

Source data are provided within this manuscript as a source data file. RNA sequencing is uploaded to NCBI’s GEO database under accession code GSE168487. Source data are provided with this paper.

## Source data

Source Data
