# Supplementary material for: Cardiac-specific deletion of voltage dependent anion channel 2 leads to dilated cardiomyopathy by altering calcium homeostasis
Source: Nat Commun. 2021 Jul 28;12:4583. doi: 10.1038/s41467-021-24869-0 (PMC8319341; doi:10.1038/s41467-021-24869-0)
Supplement: Supplementary file 1 — Supplementary Information [file 41467_2021_24869_MOESM1_ESM.pdf]

Supplementary Methods.

Mitochondrial respiration (OXPHOS). Mice were euthanized using sodium pentobarbital and the heart excised and placed in Biopsy Preservation Solution (BIOPS; containing (in mM) 2.77 CaK_2EGTA, 7.23 K_2EGTA, 6.56 $MgCl_2$, 0.5 DTT, 50.0 K-MES, 20.0 imidazole, 20.0 taurine, 5.77 Na_2ATP, and 15.0 phosphocreatine, pH 7.1 at 4°C). Tissues were weighted to minimize variation in sample mass, and stored in ice-cold BIOPS for 30min. BIOPS-immersed tissues were separated along the long axis with fine-tip forceps and subsequently bathed and shaken in BIOPS-saponin solution (30mg/ml) for 30min followed by rinsing twice for 10min of MIR05 solution [containing (in mM) 2.77 CaK_2EGTA, 7.23 K_2EGTA, 6.56 $MgCl_2$, 0.5 DTT, 20.0 imidazole, 5.77 ATP, 15.0 phosphocreatine, 50.0 K-MES, and 20.0 taurine, pH 7.0].

High-resolution O_2 consumption measurements were conducted using the ORBOROS Oxygraph-2k (Oroboros Instruments, Innsbruck, Austria) at 37 °C. Tissues were placed in the chamber of Oroboros O2k in 2ml of MIR05 solution and continuously stirred at 37°C. After the tissues were allowed to equilibrate, mitochondrial respiratory function was assessed. To measure the function of each mitochondrial complex, O_2 consumption was assessed with the addition of a series of respiratory substrates and inhibitors in the following order and final concentrations in the chamber: (in mM) 2.0 glutamate-10.0 malate, 5.0 ADP, 10.0 succinate, 0.5µM rotenone, 10µM cytochrome *c*, and 2g/ml oligomycin. After the respiration assessments, each sample assessed for respiratory function was weighed and then frozen; these tissue weights were used for the normalization of respiration rate.

Calcium imaging. For measurement of calcium transients, ventricular cardiomyocytes of WT mice were isolated by retrograde perfusion through the aorta as described previously[1], loaded with Fluo4-AM and calcium transients were recorded on a Zeiss LSM 880 confocal microscope during field stimulation with a S48 square pulse stimulator (Grass Technologies, USA).

Supplementary Table 1.

Genotype	Gender	Age	EF(%)	EDV (ul)	ESV (ul)	FS(%)	LVAW;d (mm)	LVPW;d (mm)	LVAW;s (mm)	LVPW;s (mm)	LVEDD (mm)	LVM-C (mg)
WT	F	E19	50.20	8.98	4.47	23.60	0.54	0.03	0.69	0.23	1.74	5.98
WT	F	E19	76.00	13.10	3.18	42.00	0.29	0.22	0.48	0.48	2.03	6.69
WT	M	E19	61.00	15.60	6.20	31.00	0.40	0.76	0.77	1.03	2.17	22.40
WT	M	E19	64.20	13.10	4.70	32.80	0.43	0.50	0.69	0.69	2.02	14.80
WT	M	E19	57.10	11.70	5.10	27.90	0.58	0.39	0.60	0.43	1.94	14.70
KO	F	E19	76.00	5.00	1.20	41.20	0.42	0.38	0.45	0.39	1.38	6.50
KO	F	E19	51.00	7.80	3.80	24.00	0.36	0.34	0.48	0.43	1.64	7.10
KO	F	E19	64.10	7.20	2.60	32.10	0.28	0.36	0.37	0.45	1.60	5.97
KO	M	E19	44.00	18.30	10.30	20.40	0.51	0.25	0.77	0.44	2.30	13.90
KO	M	E19	58.00	9.30	3.89	28.20	0.50	0.33	0.58	0.43	1.77	10.10
**t-test p-value**			0.670541895	0.288503361	0.82976043	0.64226969	0.619139583	0.720591878	0.203860202	0.35444948	0.207737521	0.26081477
WT	M	1 week	69.00	16.40	5.20	36.30	0.51	0.36	0.66	0.92	2.20	15.50
WT	M	1 week	80.00	9.20	1.70	46.00	0.43	0.91	0.96	1.11	1.76	20.40
WT	F	1 week	75.00	10.40	2.60	42.00	0.31	0.70	0.62	0.93	1.85	14.36
WT	M	1 week	70.00	17.60	5.30	37.10	0.58	0.95	1.01	1.42	2.26	36.20
WT	F	1 week	61.00	17.50	6.90	30.70	0.56	0.47	0.79	0.54	2.27	20.31
KO	F	1 week	83.00	18.10	3.03	47.90	0.45	0.52	0.90	1.00	2.30	19.20
KO	F	1 week	71.00	29.30	8.40	39.00	0.56	0.59	0.87	0.99	2.79	33.60
KO	M	1 week	69.00	10.60	3.20	36.50	0.73	0.80	0.98	0.94	1.86	27.20
KO	M	1 week	74.00	6.12	1.23	41.00	0.47	0.54	0.78	0.87	2.16	18.40
KO	F	1 week	74.50	6.10	1.55	40.20	0.91	0.54	1.31	0.58	1.50	18.80
**t-test p-value**			0.432821479	0.971864454	0.60856947	0.46311517	0.186892272	0.552565042	0.224284198	0.533266897	0.829175282	0.684579556
WT	F	4 week	70.80	40.30	11.70	39.00	0.80	0.77	1.40	1.10	3.18	63.10
WT	M	4 week	73.20	29.10	7.80	40.00	1.05	1.02	1.47	1.32	2.80	78.60
WT	F	4 week	52.00	43.00	21.10	25.40	0.72	0.65	0.99	0.84	3.30	54.80
WT	M	4 week	50.00	47.60	24.10	24.30	0.65	0.75	0.97	0.93	3.40	59.90
WT	M	4 week	60.00	53.00	28.00	29.00	0.99	0.64	1.40	1.00	3.80	90.60
KO	F	4 week	47.00	50.00	26.00	23.00	0.79	0.68	1.04	0.87	3.50	66.00
KO	F	4 week	45.00	94.00	51.00	22.00	0.68	0.69	1.04	0.96	4.60	95.00
KO	M	4 week	39.00	77.40	47.20	19.00	0.84	0.63	1.13	1.07	4.18	90.30
KO	M	4 week	50.00	65.50	32.10	25.40	0.56	0.75	0.84	1.07	3.90	69.30
KO	F	4 week	52.80	70.60	33.20	26.80	0.60	0.44	1.24	0.77	4.01	54.60
**t-test p-value**			**0.034852075**	**0.011940732**	**0.01398987**	0.067053407	0.156814148	0.180474805	0.181190592	0.40404127	**0.015585112**	0.592199035
WT	F	8 week	51.00	66.60	32.80	25.50	0.67	0.89	0.97	1.23	3.90	88.00
WT	M	8 week	48.00	66.00	34.90	23.60	0.67	0.95	0.99	1.13	3.92	92.80
WT	F	8 week	47.00	73.00	40.30	22.60	0.54	0.68	0.95	1.06	4.00	68.40
WT	M	8 week	63.00	43.80	16.20	33.30	0.82	1.08	1.21	1.52	3.20	88.20
WT	M	8 week	55.00	59.20	26.90	27.70	0.78	0.79	1.16	1.17	3.70	82.10
KO	M	8 week	45.90	73.40	40.00	22.60	0.80	0.93	1.16	1.15	4.10	108.80
KO	M	8 week	37.50	86.90	54.40	17.90	0.64	0.93	0.86	1.09	4.40	106.70
KO	M	8 week	32.20	80.80	54.80	15.10	0.95	0.76	1.06	0.97	4.23	113.20
KO	F	8 week	41.00	73.90	43.50	19.80	0.89	0.71	1.15	0.84	4.10	97.60
KO	F	8 week	28.10	96.50	69.40	13.10	0.75	0.74	0.80	0.99	4.60	107.40
**t-test p-value**			**0.006065393**	**0.014635108**	**0.01055422**	**0.008455039**	0.169574585	0.472063682	0.618164153	0.060682026	**0.016956878**	**0.002852524**

Supplementary Table 1: Echocardiographic data performed on WT and KO mice at different timepoints (n=5).

p-value: unpaired two-tailed t-test performed in all comparisons.

Supplementary Figure 1

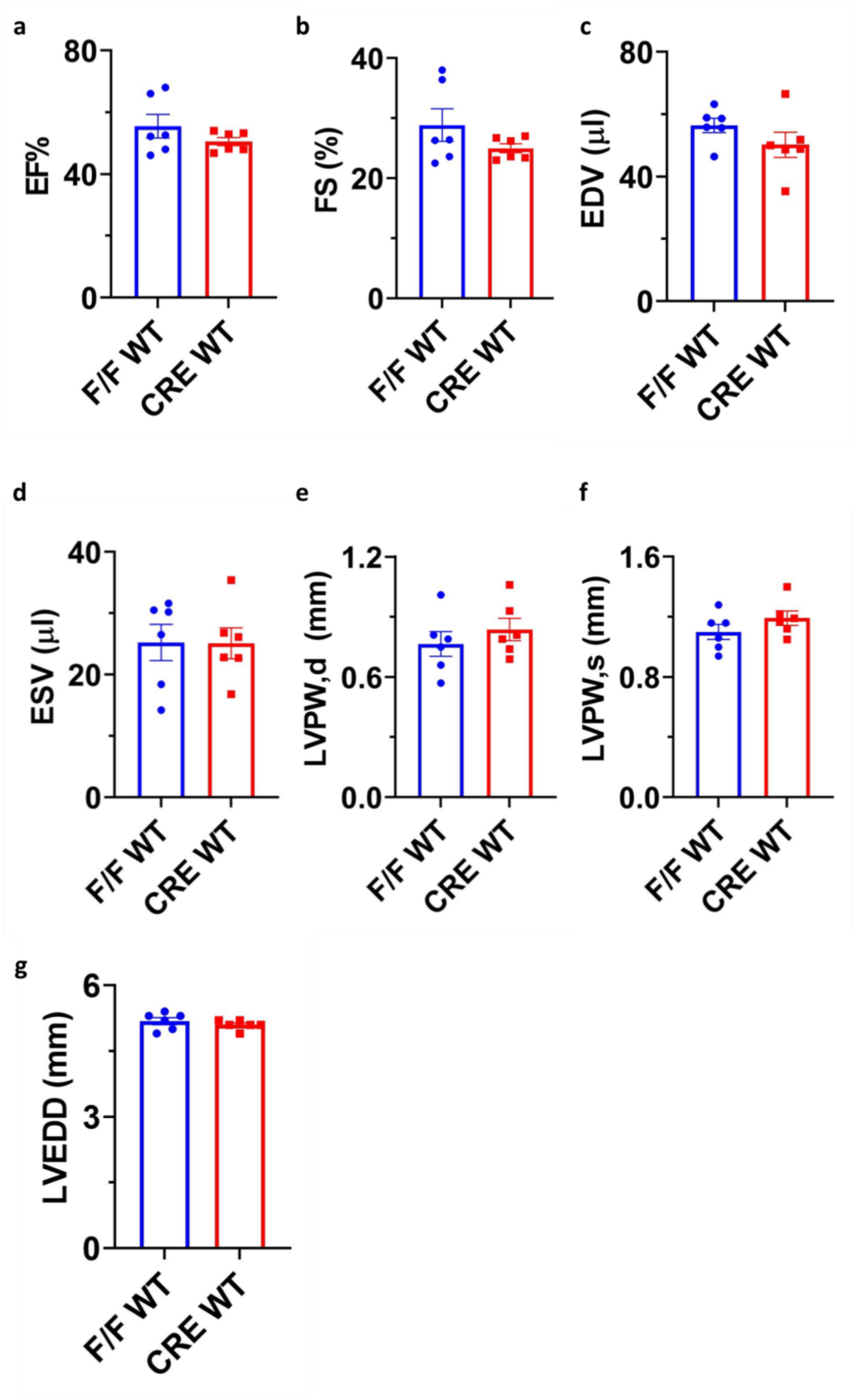

Supplementary Figure 1: No significant difference between flox-only and Cre-only mice. a-g Echocardiographic analysis of left ventricular ejection fraction (EF) (p=ns), fractional shortening (FS) (p=ns), left ventricular end-diastolic and end-systolic volumes (EDV and ESV (p=ns)), left-ventricular posterior wall thickness at diastole and systole (LVPW,d and LVPW,s (p=ns)), and left ventricular end diastolic diameter (LVEDD) (p=ns) in 16-week old WT mice respectively, (n=6).

p-value: unpaired two-tailed t-test performed in all comparisons. Data are represented as difference between mean±SEM.

Supplementary Figure 2.

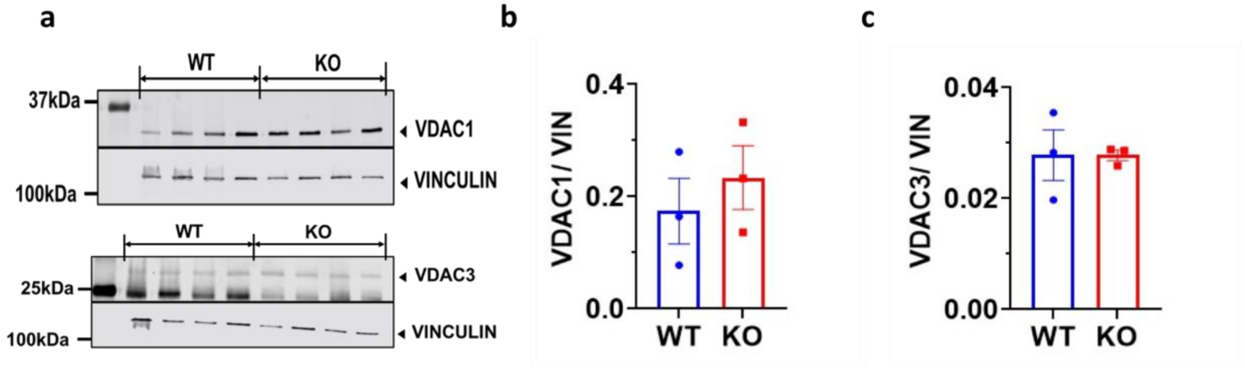

Supplementary Figure 2: VDAC2-dependent phenotype confirmation. a Western blot image of VDAC1 and VDAC3 along with respective lane-loading control VINCULIN (n=4); **b-c** Western blot quantification OF VDAC1 and VDAC3 using Image Studio Lite (version5.2.5) (p=ns) (n=4).

p-value: unpaired two-tailed t-test performed in all comparisons. Data are represented as difference between mean±SEM

Supplementary Figure 3.

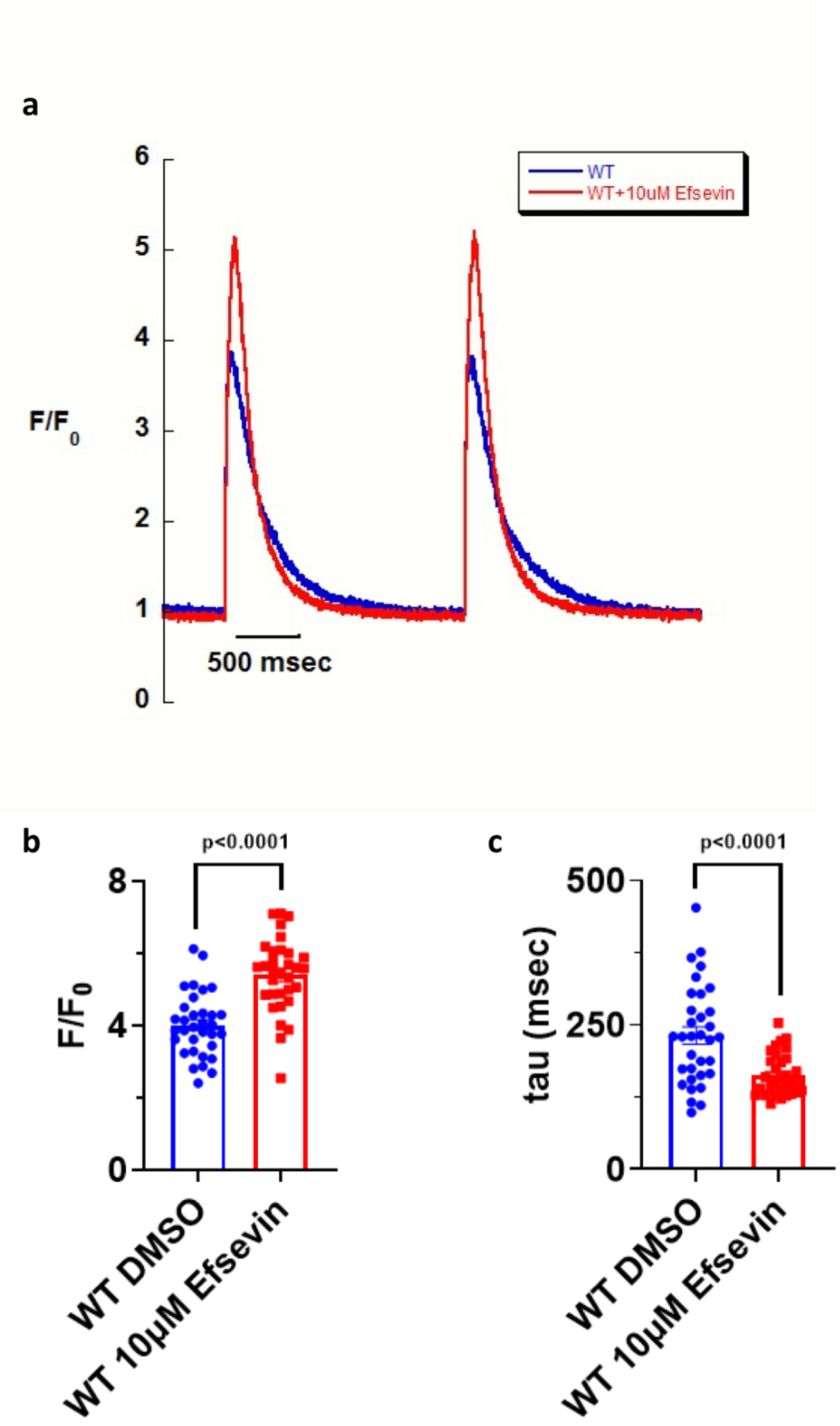

Supplementary Figure 3: Efsevin treatment shows opposite effect as observed in VDAC2-KO. a Representative calcium transient image (n=3); **b** Amplitude (F/F_0) ($p<0.0001$); **c** time constant (tau) (msec) ($p<0.0001$) (WT n=33, WT+10μM Efsevin n=32, N=3).

p-value: unpaired two-tailed t-test performed in all comparisons. Data are represented as difference between mean±SEM.

Supplementary Figure 4.

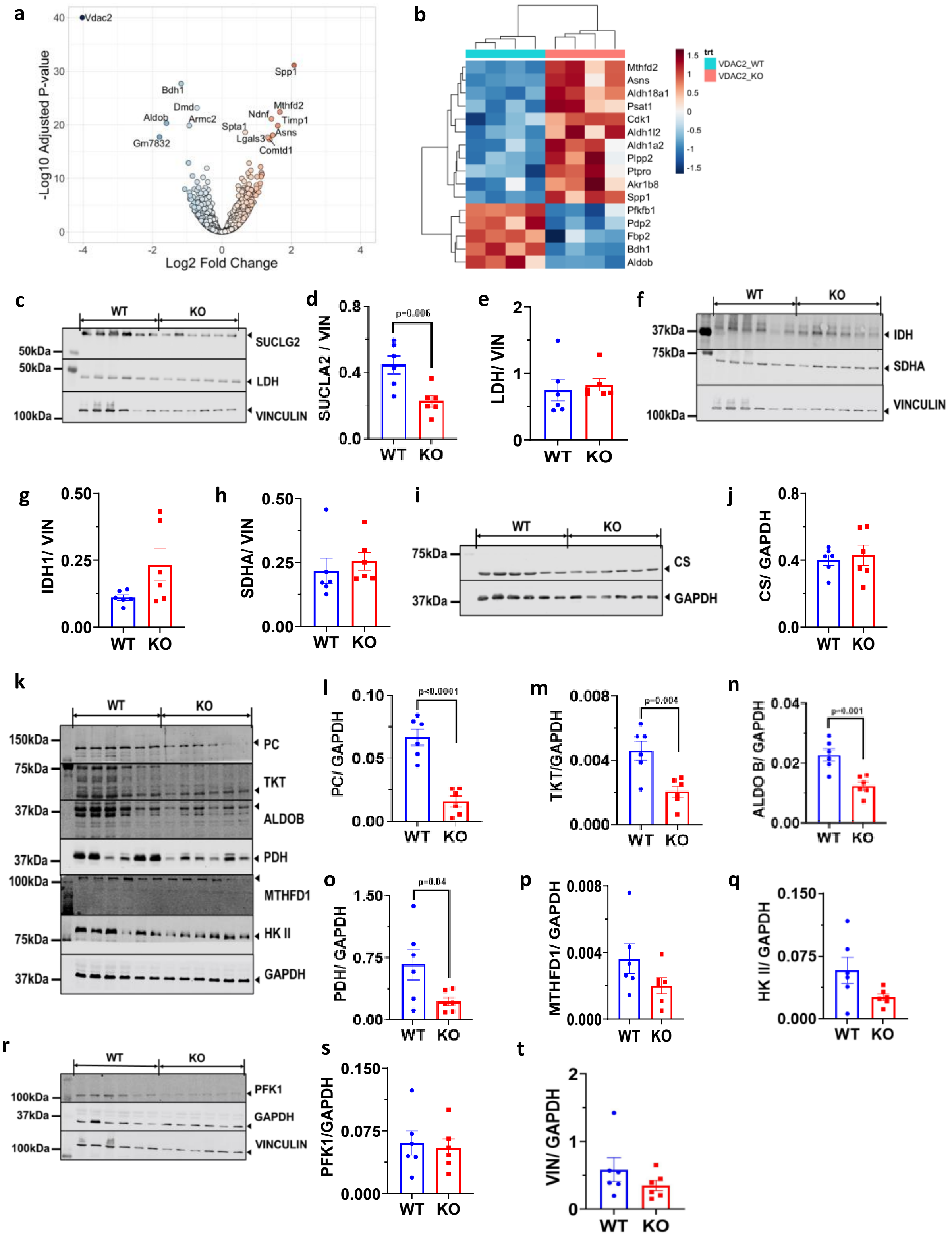

Supplementary Figure 4: Significantly reduced glycolysis in VDAC2 KO. a Volcano plot (n=4); **b** Differential gene expression profile showing altered metabolism in *Vdac2* KO mice (n=4); **c-e** Western blot image of SUCLG2 and LDH along with lane-loading control VINCULIN and quantification using Image Studio Lite (Version5.2.5) (n=6); **f-h** Western blot image of IDH1 and SDHA along with lane-loading control VINCULIN and quantification using Image Studio Lite (Version5.2.5) (n=6); **i-j** Western blot image of CS and lane-loading control GAPDH and quantification using Image Studio Lite (Version5.2.5) (n=6); **k-q** Western blot image of PC, TKT, ALDOB, PDH, MTHFD1 and HK II along with lane-loading control GAPDH and quantification using Image Studio Lite (Version5.2.5) (n=6); **r-t** Western blot image of PFK and VINCULIN along with lane-loading control GAPDH and quantification using Image Studio Lite (Version5.2.5) (n=6). Samples were derived from the same experiment and the gels were run in parallel.

p-value: unpaired two-tailed t-test performed in all comparisons. Data are represented as difference between mean±SEM.

Supplementary Figure 5.

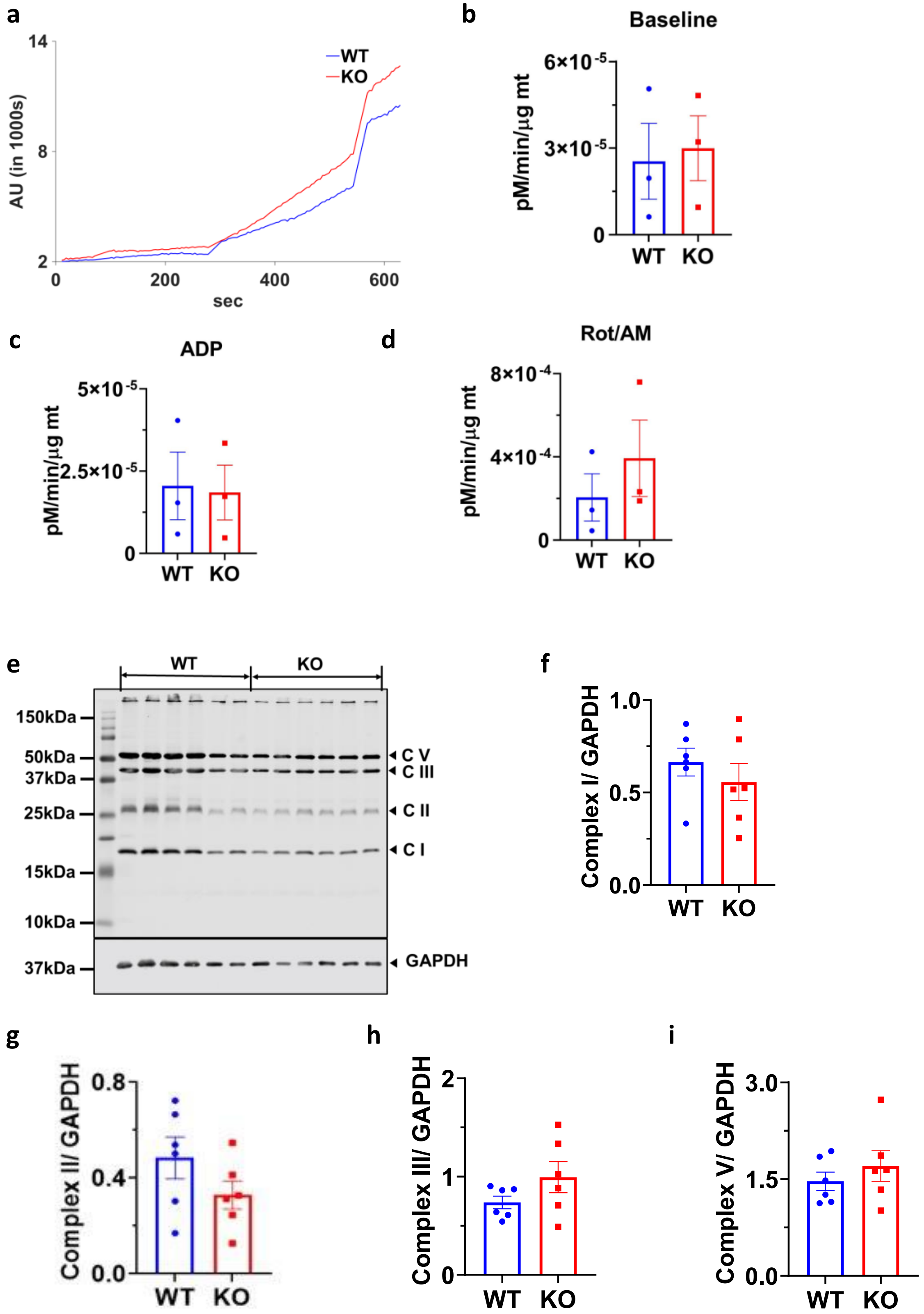

Supplementary Figure 5: Unaltered ROS production and ETC complex in VDAC2 KO. a-d Mitochondrial ROS production assay (n=3); **e** Western blot image of electron transport chain (ETC) complexes and lane-loading control GAPDH (n=6); **f-i** Western blot quantification of each complex using Image Studio Lite (version5.2.5) (n=6). Samples were derived from the same experiment and the gels were run in parallel.

p-value: unpaired two-tailed t-test performed in all comparisons. Data are represented as difference between mean±SEM

Supplementary Figure 6.

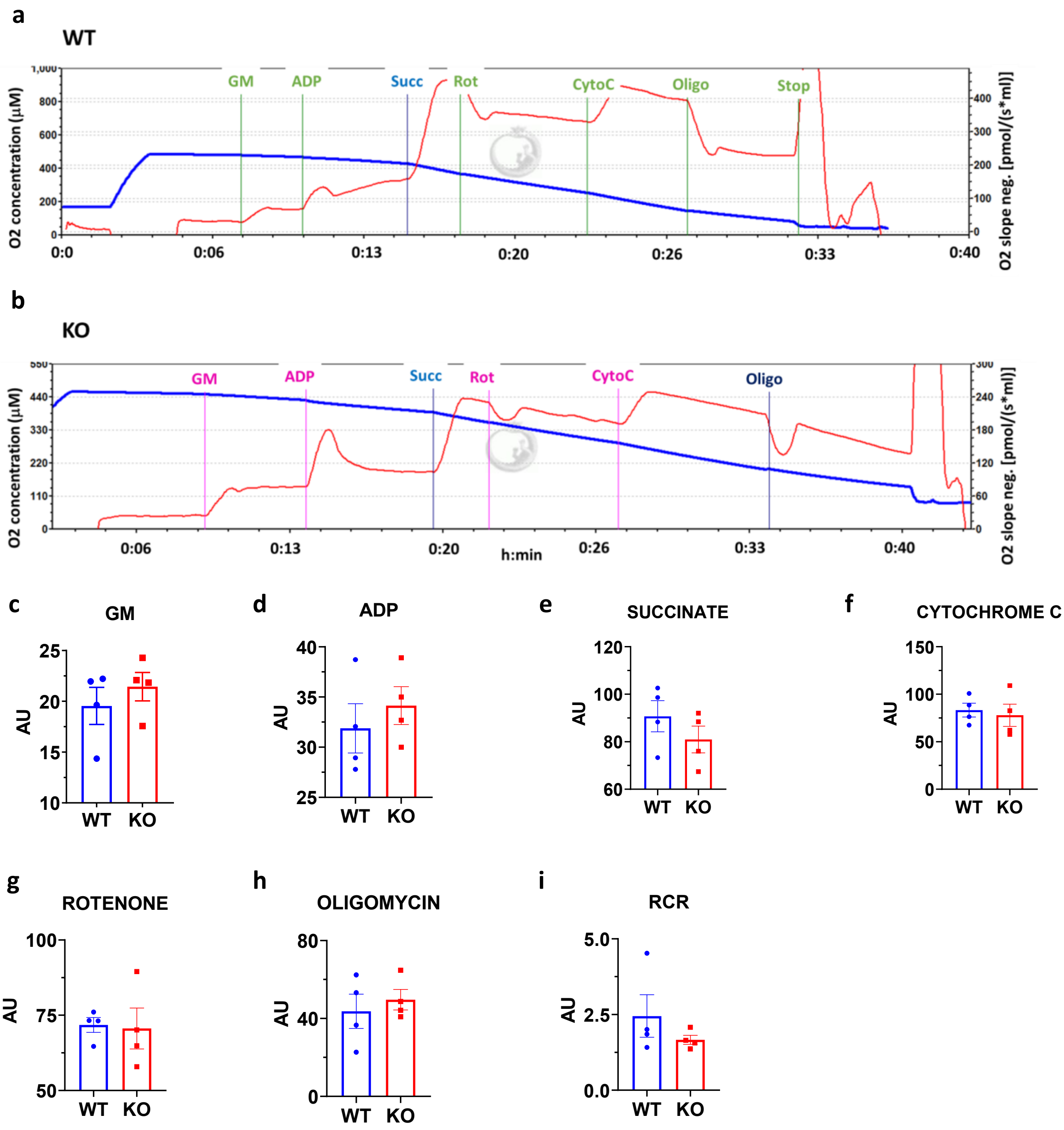

Supplementary Figure 6: No difference in mitochondrial respiration. a-b Representative mitochondrial respiration figure in WT and KO respectively (n=4); **c-i** Mitochondrial respiration quantification (n=4).

p-value: unpaired two-tailed t-test performed in all comparisons. Data are represented as difference between mean±SEM

Supplementary Figure 7.

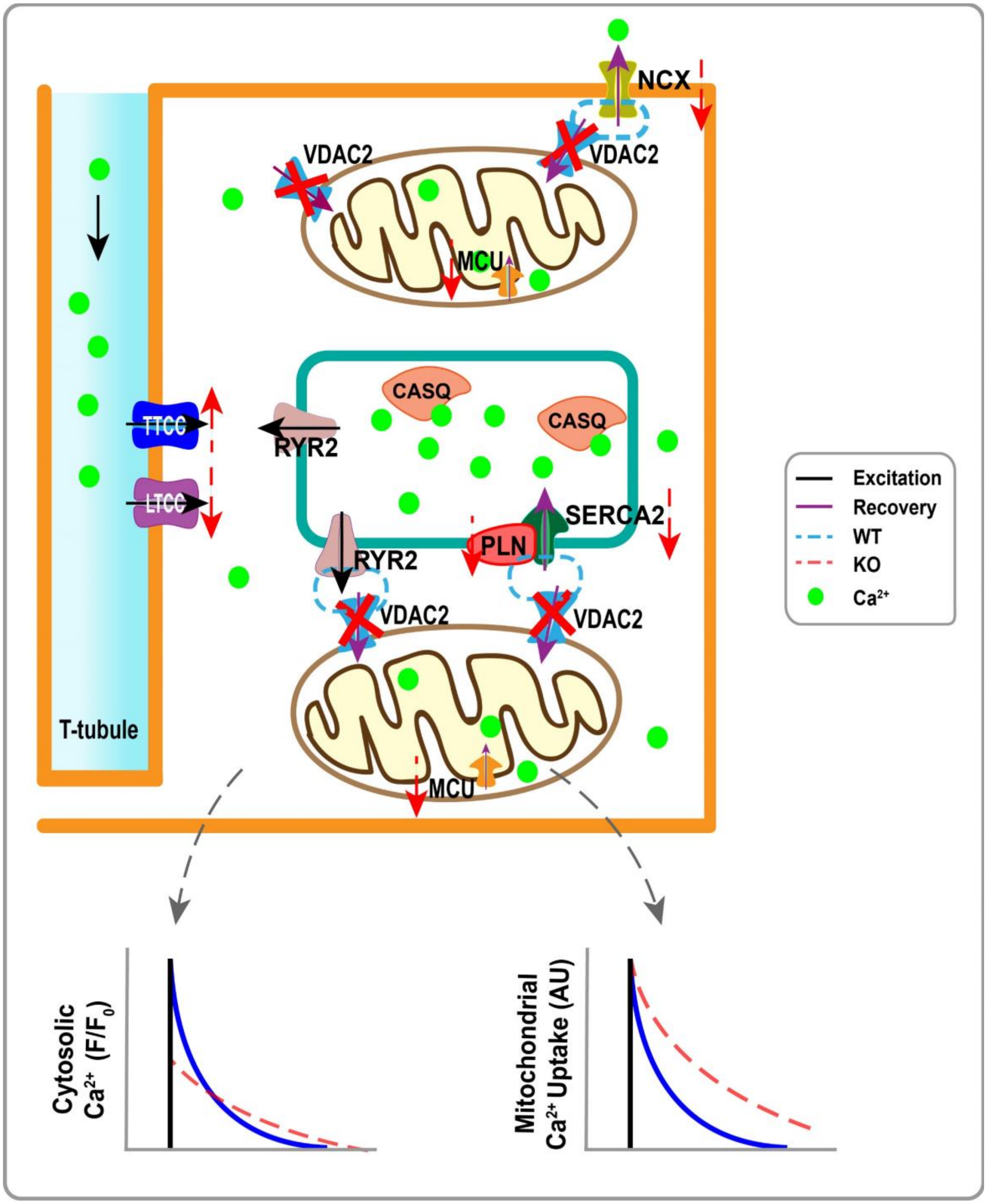

Supplementary Figure 7: **Insights into the role of VDAC2 in mitochondrial and cytosolic calcium signalling and excitation-contraction coupling.**

Abbreviations: LTCC – L-type calcium channel, TTCC – T-type calcium channel, Ca^{2+} - calcium, SR- sarcoplasmic reticulum, RyR2 – Ryanodine receptor 2, CASQ – calsequestrin, PLN – phospholamban, SERCA2A - sarcoplasmic reticulum calcium ATPase 2a, NCX – sodium calcium exchanger, MCU – mitochondrial calcium uniporter, VDACs - voltage dependent anion channels and VDAC2 – voltage dependent anion channel 2.

Supplementary Reference

1 Schweitzer, M. K. *et al.* Suppression of Arrhythmia by Enhancing Mitochondrial Ca(2+) Uptake in Catecholaminergic Ventricular Tachycardia Models. *JACC Basic Transl Sci* **2**, 737-747, doi:10.1016/j.jacbts.2017.06.008 (2017).
