# Supplementary material for: Cardiac-specific deletion of voltage dependent anion channel 2 leads to dilated cardiomyopathy by altering calcium homeostasis
Source: Nat Commun. 2021 Jul 28;12:4583. doi: 10.1038/s41467-021-24869-0 (PMC8319341; doi:10.1038/s41467-021-24869-0)
Supplement: Supplementary file 3 — Reporting Summary [file 41467_2021_24869_MOESM3_ESM.pdf]

nature research

Corresponding author(s): STAVROS G. DRAKOS

Last updated by author(s): Jun 9, 2021

Reporting Summary

Nature Research wishes to improve the reproducibility of the work that we publish. This form provides structure for consistency and transparency in reporting. For further information on Nature Research policies, see our Editorial Policies and the Editorial Policy Checklist.

Statistics

For all statistical analyses, confirm that the following items are present in the figure legend, table legend, main text, or Methods section.

n/a	Confirmed	
☐	☒	The exact sample size (*n*) for each experimental group/condition, given as a discrete number and unit of measurement
☐	☒	A statement on whether measurements were taken from distinct samples or whether the same sample was measured repeatedly
☐	☒	The statistical test(s) used AND whether they are one- or two-sided<br>*Only common tests should be described solely by name; describe more complex techniques in the Methods section.*
☐	☒	A description of all covariates tested
☐	☒	A description of any assumptions or corrections, such as tests of normality and adjustment for multiple comparisons
☐	☒	A full description of the statistical parameters including central tendency (e.g. means) or other basic estimates (e.g. regression coefficient) AND variation (e.g. standard deviation) or associated estimates of uncertainty (e.g. confidence intervals)
☐	☐	For null hypothesis testing, the test statistic (e.g. *F*, *t*, *r*) with confidence intervals, effect sizes, degrees of freedom and *P* value noted<br>*Give P values as exact values whenever suitable.*
☒	☐	For Bayesian analysis, information on the choice of priors and Markov chain Monte Carlo settings
☒	☐	For hierarchical and complex designs, identification of the appropriate level for tests and full reporting of outcomes
☒	☐	Estimates of effect sizes (e.g. Cohen's *d*, Pearson's *r*), indicating how they were calculated

Our web collection on statistics for biologists contains articles on many of the points above.

Software and code

Policy information about availability of computer code

Data collection	*LabChart Reader, Biotek Cytation 5, PCLAMP 8 software.*
Data analysis	*Zen5 Imaging software, Image Studio Lite (version5.2.5), Adobe Photoshop CC 2019, Fiji (version 2.0), GraphPad Prism (version8.2.1), Ingenuity pathway analysis software, Aperio Image Scope software (version12.3.2.8013), vivo strain software (version3.1.1).*

For manuscripts utilizing custom algorithms or software that are central to the research but not yet described in published literature, software must be made available to editors and reviewers. We strongly encourage code deposition in a community repository (e.g. GitHub). See the Nature Research guidelines for submitting code & software for further information.

Data

Policy information about availability of data

All manuscripts must include a data availability statement. This statement should provide the following information, where applicable:

- Accession codes, unique identifiers, or web links for publicly available datasets
- A list of figures that have associated raw data
- A description of any restrictions on data availability

All data are available in NCBI's GEO and accession code is GSE168487 [https://www-ncbi-nlm-nih-gov.ezproxy.u-pec.fr/geo/query/acc.cgi?acc=GSE168487].

Field-specific reporting

Please select the one below that is the best fit for your research. If you are not sure, read the appropriate sections before making your selection.

☒ Life sciences ☐ Behavioural & social sciences ☐ Ecological, evolutionary & environmental sciences

For a reference copy of the document with all sections, see nature.com/documents/nr-reporting-summary-flat.pdf

Life sciences study design

All studies must disclose on these points even when the disclosure is negative.

Sample size	n=3 mice were used for AAV injection, cellular studies fibrosis analysis and imaging studies. n=4 were used for RNA sequencing studies and n=6 mice were used for echocardiography and western blot studies given it's the standard practice.
Data exclusions	No data was excluded except 1 WT sample was removed initially in RNA sequencing analysis due to poor RNA quality.
Replication	All studies have biological and technical (n=3) replicates with follow-up validation. The results were consistent across the replicates.
Randomization	Mice were randomly chosen based on their genotype.
Blinding	Samples were blinded during AAV9 injections and during analysis of results (including echocardiography analysis, western blot quantification, calcium transients, fibrosis analysis, etc.)

Reporting for specific materials, systems and methods

We require information from authors about some types of materials, experimental systems and methods used in many studies. Here, indicate whether each material, system or method listed is relevant to your study. If you are not sure if a list item applies to your research, read the appropriate section before selecting a response.

Materials & experimental systems

n/a	Involved in the study	
☐	☒	Antibodies
☒	☐	Eukaryotic cell lines
☒	☐	Palaeontology and archaeology
☐	☒	Animals and other organisms
☒	☐	Human research participants
☒	☐	Clinical data
☒	☐	Dual use research of concern

Methods

n/a	Involved in the study	
☒	☐	ChIP-seq
☒	☐	Flow cytometry
☒	☐	MRI-based neuroimaging

Antibodies

Antibodies used

Western blot-Antibodies
Voltage-dependent anion-selective channel protein 2 (VDAC2) 9412S Cell Signaling Technology
Voltage-dependent anion-selective channel protein 1 (VDAC1) ab14734 Abcam
Voltage-dependent anion-selective channel protein 3 (VDAC3) ab130561 Abcam
Vinculin 13901S Cell Signaling Technology
Glyceraldehyde 3-phosphate dehydrogenase (GAPDH) 5174S Cell Signaling Technology
Transketolase (TKT) 8616S Cell Signaling Technology
Methylenetetrahydrofolate dehydrogenase 1 (MTHFD1) sc-271412 Santa Cruz Biotech
Aldolase B (ALDOB) sc-365449 Santa Cruz Biotech
Serine hydroxymethyltransferase 1 (SHMT1) 80715 Cell Signaling Technology
Isocitrate dehydrogenase (IDH1/2) sc-373816 Santa Cruz Biotech
Phosphofructokinase 1 (PFK1) sc-166722 Santa Cruz Biotech
Pyruvate carboxylase (PC) NBP1-49536 Novus Bio
Pyruvate dehydrogenase (PDH) 3205S Cell Signaling Technology
Lactate dehydrogenase (LDH) 3582S Cell Signaling Technology
Hexokinase 1 (HK1) 2024S Cell Signaling Technology
Calsequestrin 2 (CASQ2) ab3516 Abcam
Citrate synthase (CS) 14309S Cell Signaling Technology
Succinyl Co-A synthetase (SUCLG2) 8071S Cell Signaling Technology
Succinate dehydrogenase A (SDHA) 5839S Cell Signaling Technology
Sodium-calcium exchanger 1 (NCX1) ab151608 Abcam
Sarcoplasmic endoplasmic reticular calcium ATPase 2 (SERCA2) 4388S Cell Signaling Technology
Ryanodine Receptor 2 (RYR2) MA3-916 Thermofisher Scientific
Phospholamban (PLN) 14562S Cell Signaling Technology
Phospho-Phospholamban (pPLN) 8496S Cell Signaling Technology

Protein kinase A (PKA) SAB4502337 Sigma Aldrich
Phospho-Protein kinase A (pPKA) SAB4503969 Sigma Aldrich
Mitochondrial calcium uniporter (MCU) 14997S Cell Signaling Technology
Mitochondrial sodium calcium exchanger (NCLX) SAB2102181 Sigma Aldrich
Dynamin related protein 1 (DRP1) 14647S Cell Signaling Technology
Oxidative phosphorylation (OXPHOS) ab110413 Abcam
IRDye 680RD Donkey anti-Mouse Secondary 926-68072 LI-COR Biosciences
IRDye 800CW Donkey anti-Rabbit Secondary 926-32212 LI-COR Biosciences
Co-Immunoprecipitation-Antibodies
Voltage-dependent anion-selective channel protein 2 (VDAC2) PA5-28106 Thermofisher Scientific
Immunoglobulin G (IgG) AC005 AbClonal
Immunofluorescence/Proximity ligation assay-Antibodies/Stain
4',6-diamidino-2-phenylindole (DAPI) D3571 Thermofisher Scientific
Voltage-dependent anion-selective channel protein 2 (VDAC2) 66388-1-IG Thermofisher Scientific
Sodium-calcium exchanger 1 (NCX1) ab151608 Abcam
Sarcoplasmic endoplasmic reticular calcium ATPase 2 (SERCA2) 4388S Cell Signaling Technology
Ryanodine Receptor 2 (RYR2) MA3-916 Thermofisher Scientific
Quantitative Reverse transcription-PCR-Primers
Voltage-dependent anion-selective channel protein 2 (VDAC2) Mm00834279_m1 Thermofisher Scientific
Vinculin Mm00447745_m1 Thermofisher Scientific

Validation

All antibodies were previously tested by the company and used in other publications. A separate file title "Antibody list and validation" includes information from the website and relevant publications for each of the antibody.

Animals and other organisms

Policy information about studies involving animals; ARRIVE guidelines recommended for reporting animal research

Laboratory animals

C57BL6J mice strain and both male and female mouse were used for all experiments. Echocardiography was performed on mice starting from embryonic day 18.5 until 16-weeks post-natal. Calcium transients were performed on 16-week old mice and co-IP on 8-week old mice.

Wild animals

Study did not involve wild animals

Field-collected samples

Study did not involve samples collected from the field

Ethics oversight

Studies were performed following guidelines established by IACUC, University of Utah.

Note that full information on the approval of the study protocol must also be provided in the manuscript.
