# Supplementary material for: Cardiac-specific deletion of voltage dependent anion channel 2 leads to dilated cardiomyopathy by altering calcium homeostasis
Source: Nat Commun. 2021 Jul 28;12:4583. doi: 10.1038/s41467-021-24869-0 (PMC8319341; doi:10.1038/s41467-021-24869-0)
Supplement: Supplementary file 2 — Peer Review File [file 41467_2021_24869_MOESM2_ESM.pdf]

REVIEWER COMMENTS

Reviewer #1 (Remarks to the Author):

This paper presents results showing that cardiac-specific deletion of Vdac2 in mice resulted in dilated cardiomyopathy by altering calcium homeostasis, leading to adverse cardiac remodeling which progressed to severe cardiomyopathy and death. Myocytes lacking VDAC2 showed severe impairment in excitation-contraction coupling by altering both intracellular and mitochondrial calcium signaling. The authors conclude that VDAC2 plays a crucial role during cardiac development by influencing cellular calcium signaling.

In general, this study presents interesting information, yet it has been shown previously that VDAC2 has a distinctive role in mediating SR to mitochondria local Ca2+ transport in cardiomyocytes. VDAC2 is required to locally couple with mitochondrial Ca2+ uptake with RyR2-mediated Ca2+ release in the heart (Ref. 10).
Comments
1. The following findings should be included in the Introduction: The lethal consequences of Vdac2 knockout in mouse embryos (Cheng EH, Sheiko TV, Fisher JK, Craigen WJ, Korsmeyer SJ. VDAC2 inhibits BAK activation and mitochondrial apoptosis. Science. 2003;301:513–51).
In addition, the controversial findings about VDAC2 function in apoptosis as a promoter or inhibitor of death, as summarized in (Ref. 6) should be indicated.

2. The expression of many proteins related to Ca2+ level regulation were tested. However, RyR2, the channel responsible for the release of Ca2+ from the SR and presented as a key important protein for the functional and structural link between the SR and mitochondria (VDAC2) was not tested.
3. Blots are very small and it is hard to read the text, and for some, the quality is not very good.
4. It should be indicated in the figure that blots from 3 different mice are shown (as Fig. 1a, 2c, 4d,I, sup 2c).
5. Similarly, several panels in the figures are hard to read such as Fig. 3f–h, Fig. 5 b, Supplementary fig.2a, 2c, Supp 3e, Supp 4a,b
6. I wonder why anti-VDAC1 antibodies from Santa Cruz Bio. (sc), #390996) were used and not the known more specific anti-VDAC from Abcam.

Reviewer #2 (Remarks to the Author):

The voltage dependent anion channel 2 (VDAC2) is located in the mitochondrial outer membrane and contributes to apoptosis, steroidogenesis, metabolite flux and calcium homeostasis6.Previous study has shown that VDAC2 interacts with RYR2 and is essential for the transfer of Ca2+ from the sarcoplasmic reticulum to the mitochondria. In the present study, the authors generated a cardiomyocyte-specific developmental deletion of Vdac2 in mice to elucidate the role of VDAC2 in calcium homeostasis. Vdac2 depletion caused severe impairment in excitation-contraction coupling by altering both intracellular and mitochondrial calcium signaling. They authors combined different approaches, including molecular analysis, RNAseq, electrophysiology, measurements of Ca transient and sarcomere shortening in intact mouse cardiomyocytes, and analysis of Ca uptake, respiration and metabolism. Conclusion is that VDAC2 plays a novel role in cellular calcium dynamics and could emerge as a therapeutic target of heart failure. Overall, they did a lot, but often with a low n number. I found several data not convincing that merit further experiments.

Major comments:
1. The authors claimed that the cardiac phenotype was assessed from embryonic day 7 (i.e. performed in pregnant mice) until postpartum week 16 and therefore that they evaluated the phenotype during the entire development. However, they only show echo data at the postnatal age of 16 wks. At what age the cardiac phenotype did start? It would be of interest to provide longitudinal echo data at least for the major parameters, such as EDLVD, FS and and LVM/BW over time. Furthermore, no parameters of LV wall thickness were given. Please add all parameters of

echo, including, gender, age, wall thicknesses in end-diastole and in end-systole and LVM/BW ratio. It could be in a table.
1. Figure 2: What is the mechanism by which VDAC2 deficiency leads to a marked decrease in the level of most proteins involved in calcium handling (SERCA2, PLN and all channels)? You showed one continuous blot for GAPDH and VIN; however, you cut the blot in the middle for NCX and CASQ2. This is not possible. Please show continuous blot for both NCX and CASQ2.
2. Previous study showed that knock-down of VDAC2 did not result in the disappearance of NCX, PLN and several other proteins. The authors should discuss these points.
3. Figure 3: The IP experiment is not convincing. I see several bands in the VDAC2 input, a major band at a lower MW in the SERCA2 input, major bands at a lower MW in the NCX input. In addition, NCX has 2 MW, one about 100 kDa and another one at 160 kDa, so it does not fit. I guess what you see with your NCX antibody is not NCX. A positive control for IP is required such as RYR2. The anti-VDAC2 antibody is not fully specific. Immunofluorescence analysis of VDAC2-SERCA2 and VDAC2-NCX should be also shown. Proximity-ligation assay could also be done to support proximity of VDAC2 with the respective protein partners.
2. AP- The representative AP is not representative since APD90 looks markedly shorter in the KO, suggestive of higher heart rate. I am not convinced. More cells are required.
3. The C57/BL6 mice are on two different backgrounds (J or N) that could markedly alter mitochondrial function and metabolism. The authors should write which background has been taken and discussed the possible implication.
4. Alpha-MHC Cre per se can induced cardiac dysfunction. The authors said that they used either flox or Cre as control, which I found not appropriate. It would be important to show echo data performed on Flox, Cre and VDAC2-KO mice to see whether the controls behave similarly.
5. Most importantly, the authors should test whether efsevin in wild-type mice induced the same phenotype as in KO mice.

Minor comments:
4. Replace protein expression by protein level. Figure 1a – please give a darker blot for VDAC2 in KO mice.
5. Figure 1b: how can you do statistics with no VDAC2 in KO mice? Anything compared to 0 is not possible. Why did you get any values in KO mice?
6. Figure 1k-m: how many mice were used. Please add the N7n (number of mice / number of cells) analysed.
7. Figure 2: what is VIN as an endogenous loading control?
8. Why did you use a miRNeasy Mini kit to extract total RNA?

Reviewer #3 (Remarks to the Author):

This submission is an interesting, well-designed study whose premise is that cardiomyocyte-specific developmental deletion of Vdac2 in a mouse model contributes to a pathological phenotype described in the paper by severe impairment in excitation-contraction coupling, altered calcium signaling in the cytosol and mitochondria, and adverse cardiac remodeling (fibrosis) which increased mortality in the KO mouse model. The supporting evidence for this paper's premise, namely the importance of VDAC2 in cardiac physiology, is described in previous work by this group as well as others in the field. Overall, the methods described in this submission are robust and support the hypothesis, although some clarification and additional data presentation would increase the interpretability and impact.

Some specific issues, comments and suggestions this reviewer has for the submission are as follows:
1. The authors do not indicate which timepoint is being compared in the echocardiography data represented in Fig.1d-g. The manuscript claims that serial echo was performed from E7 until 16 weeks postpartum, but the authors need to indicate at what age the data in these panels are representing, as cardiac function does not seem to deteriorate until ~5w of age postnatally per Fig.1c.
2. Additionally, the authors claim "Increased mortality was observed in the KO mice between 5-20 weeks postpartum. Most of these events occurred during 20 weeks postpartum." It is worth noting

that these are not conditional KO mice, where Vdac2 is turned off at multiple embryonic time points (eg, formation of the FHF/SHF). Hearts represented in Fig.1i is a comparison of KO to WT at what age? 16w (If same as the echo data in Fig.1h), but the authors' interpretation of the findings insinuate that fetally and at birth, Vdac2KO hearts are phenotypically and functionally identical to WT hearts. This does not indicate a developmental role for Vdac2 in the murine heart, but rather a functional role postnatally, whose loss has a detrimental effect on cardiac myocytes over time. Reviewer would caution against using the term "developmental importance" versus "functional importance."

3. Additionally, gene expression array was performed at 16w postpartum. I am curious if the authors performed any gene or protein expression analysis at earlier time points, such as in utero or acutely post-natally. While the data are convincing that "Collectively, these results suggest that loss of VDAC2 leads to abnormalities in the calcium signaling pathways" if the authors are to suggest a developmental role for VDAC2, only checking related Ca2+ handling proteins once an observable phenotype is attained does not give a comparable baseline of these critical CICR proteins.

4. It is demonstrated that SERCA2, NCX, total and phosphorylated phospholamban (PLN), and calsequestrin (CASQ2, the SR calcium binding protein) were decreased in the KO, but did the authors check for changes in RyR expression?

5. In Fig3h, why is the band lower in the input than in the IP lane in the VDAC2-NCX coIP? Additionally, the IP lane contains a doublet whereas the input lysate for NCX is a singlet band. Could the authors explain these discrepancies between the IP pull-down and non-IPed endogenous protein?

6. Fig3.i – the WT HR measurements have no observable variance between animals. These numbers seem too good to be true, based on experience, and reviewer is curious why all the animals have identical HRs.

7. 3a through 3e are very well-done however, as is the TEM in Fig.4

8. The volcano plot from the RNAseq data are interesting, particularly the observation of an overall downregulation of enzyme levels involved in glycolysis, TCA cycle, and glycolytic pathways. However, no significant difference was observed in FAO pathways Given that FAO is the primary fuel source for adult cardiac myocytes, this may actually have more developmental consequences in utero and acutely postnatally. What are the implications in the adult heart? Are there changes in substrate utilization in genetic DCM hearts (human or murine)? Is this reduction in glycolytic pathway components observed during fetal cardiac development.

General comments:

1. This paper would be much stronger with a rescue of the KO model utilized. This is easily achievable by the senior author's group; AAV9-targeted VDAC2 expression and subsequent analysis of the phenotype – which can be performed before and after the decline in cardiac function as seen in the echo data – would have increased the impact of this paper. Likewise, the reviewer is curious whether the authors have considered an inducible VDAC2-KO model, particularly with the mention of developmental importance of this protein in calcium handling. Comparing in utero (fetal) to postnatal loss of VDAC2 would provide a wealth of information which may explain the changes in expression and activity of related proteins, particularly the ion channels. However, these are not suggestions which preclude publication for an otherwise compelling and interesting paper.

We thank the editors and reviewers for their insightful comments and careful review of our paper. Please find below our response to each comment. As recommended by the editor, we have addressed Reviewer 2 concern on the appropriate use of wild-type mice and Reviewer 3 suggestion on the overexpression model. In many cases, these recommendations have led to additional experiments and significant improvement in the manuscript. The changes made in the revised manuscript are highlighted for easier identification.

REVIEWER COMMENTS

Reviewer #1 (Remarks to the Author):

This paper presents results showing that cardiac-specific deletion of Vdac2 in mice resulted in dilated cardiomyopathy by altering calcium homeostasis, leading to adverse cardiac remodeling which progressed to severe cardiomyopathy and death. Myocytes lacking VDAC2 showed severe impairment in excitation-contraction coupling by altering both intracellular and mitochondrial calcium signaling. The authors conclude that VDAC2 plays a crucial role during cardiac development by influencing cellular calcium signaling.
In general, this study presents interesting information, yet it has been shown previously that VDAC2 has a distinctive role in mediating SR to mitochondria local Ca2+ transport in cardiomyocytes. VDAC2 is required to locally couple with mitochondrial Ca2+ uptake with RyR2-mediated Ca2+ release in the heart (Ref. 10).

Comments
1. The following findings should be included in the Introduction: The lethal consequences of Vdac2 knockout in mouse embryos (Cheng EH, Sheiko TV, Fisher JK, Craigen WJ, Korsmeyer SJ. VDAC2 inhibits BAK activation and mitochondrial apoptosis. Science. 2003;301:513–51).
In addition, the controversial findings about VDAC2 function in apoptosis as a promoter or inhibitor of death, as summarized in (Ref. 6) should be indicated.

Response 1: We appreciate this suggestion by the reviewer. We have now added a few points on apoptosis in the introduction (Pages 1-2, Lines 44-50):
"VDAC2 is known to interact with pro-apoptotic proteins such as Bcl2 family proteins. While VDAC2-BAK (Bcl2-antagonist/killer protein) interaction is reported to be highly important to control cellular apoptosis, it is still controversial if this interaction promotes or inhibits apoptosis. Studies show that VDAC2-BAK complex was absent during death stimulus and deletion of VDAC2 promotes apoptosis[1]. In contrast, other studies have shown that truncated-Bid (BH3-interacting domain death agonist) induced MOM permeabilization and cell death in wild-type (WT) but not in VDAC2 knock-out (KO) mouse embryonic fibroblasts[2]."
Furthermore, we have added information about the global knockdown. (Page 2, Lines 69-71): "However, after a global knock-down of VDAC2 was shown to be embryonic lethal[1], no further studies have investigated the effects of cardiac VDAC2-KO in animal models."

2. The expression of many proteins related to Ca2+ level regulation were tested. However, RyR2, the channel responsible for the release of Ca2+ from the SR and presented as a key important protein for the functional and structural link between the SR and mitochondria (VDAC2) was not tested.

Response 2: This is an excellent point and we have performed western blot to check the protein levels of RyR2 in both wild-type and knock-out (KO) mice and observed that the KO mice showed a significant reduction in RyR2 levels. These results are now included in the revised manuscript (Figure 2c and j). Please see Page 4, Lines 117-119 in the revised manuscript: "Proteins involved in ECC such as RYR2, SERCA2, NCX1, total and phosphorylated phospholamban (PLN), and calsequestrin (CASQ2, the SR calcium binding protein) were significantly decreased in the KO"

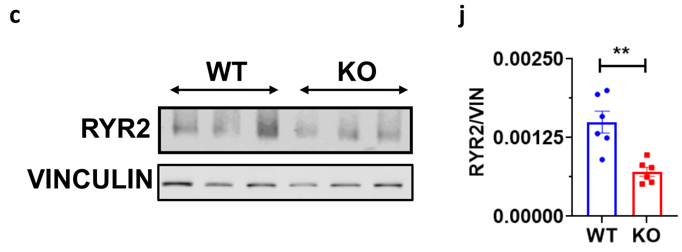

Figure 2: c Representative western blot image (n=3) and lane-loading controls; **j** Western blot quantification data using Image Studio Lite (version5.2.5) (n=6).
p-value: unpaired t-test **<0.01

3. Blots are very small and it is hard to read the text, and for some, the quality is not very good.

Response 3: We agree with the reviewer's comment. All blots (and figures) have been replaced with ones that have better quality.

4. It should be indicated in the figure that blots from 3 different mice are shown (as Fig. 1a, 2c, 4d,I, sup 2c).

Response 4: We appreciate this suggestion by the reviewer. We have indicated this information in all figures with western blots.

5. Similarly, several panels in the figures are hard to read such as Fig. 3f–h, Fig. 5 b, Supplementary fig.2a, 2c, Supp 3e, Supp 4a,b

Response 5: We appreciate this suggestion by the reviewer. All figures as indicated by the reviewer have been replaced with better quality figures.

6. I wonder why anti-VDAC1 antibodies from Santa Cruz Bio. (sc), #390996) were used and not the known more specific anti-VDAC from Abcam.

Response 6: We thank the reviewer for this question. We re-ran the samples with the Abcam antibody (Ab14734) and found no differences compared to the Santa Cruz one. Additionally, our RNA sequencing experiment (KO vs WT) does not show any significant difference in VDAC1 levels between WT and KO mice, confirming our western blot results. The blots have been replaced in the manuscript (Supplemental Figure 2a-b)

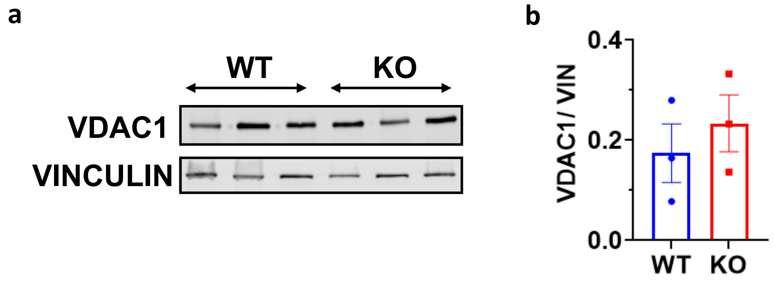

Supplemental Figure 2: **a** Representative western blot image and lane loading control (n=3); **b** Western blot quantification data using Image Studio Lite (version5.2.5) (n=3). Unpaired t-test performed.

RNA sequencing (not included in the manuscript):

Gene	p-value	FC
VDAC1	0.43310788	1.04106415

Reviewer #2 (Remarks to the Author):

The voltage dependent anion channel 2 (VDAC2) is located in the mitochondrial outer membrane and contributes to apoptosis, steroidogenesis, metabolite flux and calcium homeostasis. Previous study has shown that VDAC2 interacts with RYR2 and is essential for the transfer of Ca2+ from the sarcoplasmic reticulum to the mitochondria. In the present study, the authors generated a cardiomyocyte-specific developmental deletion of Vdac2 in mice to elucidate the role of VDAC2 in calcium homeostasis. Vdac2 depletion caused severe impairment in excitation-contraction coupling by altering both intracellular and mitochondrial calcium signaling. The authors combined different approaches, including molecular analysis, RNAseq, electrophysiology, measurements of Ca transient and sarcomere shortening in intact mouse cardiomyocytes, and analysis of Ca uptake, respiration and metabolism. Conclusion is that VDAC2 plays a novel role in cellular calcium dynamics and could emerge as a therapeutic target of heart failure. Overall, they did a lot, but often with a low n number. I found several data not convincing that merit further experiments.

Major comments:

1. The authors claimed that the cardiac phenotype was assessed from embryonic day 7 (i.e. performed in pregnant mice) until postpartum week 16 and therefore that they evaluated the phenotype during the entire development. However, they only show echo data at the postnatal age of 16 wks. At what age the cardiac phenotype did start? It would be of interest to provide longitudinal echo data at least for the major parameters, such as EDLVD, FS and and LVM/BW over time. Furthermore, no parameters of LV wall thickness were given. Please add all parameters

of echo, including, gender, age, wall thicknesses in end-diastole and in end-systole and LVM/BW ratio. It could be in a table.

Response 1: We appreciate this recommendation. As rightly pointed out by the reviewer, we performed serial echocardiography on mice until 16-weeks of age. As shown in Figure 1b (in the revised manuscript), we observed variable mortality (from 5-weeks of age up to 20-weeks of age). Since majority of the mice died during 18-20 weeks, the data shown below are from mice that survived until that time point (for statistical comparison reasons). Progressive deterioration of cardiac function was observed in these mice and a significant difference between the two groups (WT and KO) was observed at 16-weeks of age. We have now included these longitudinal data in Figure 1c-j (in the revised manuscript).

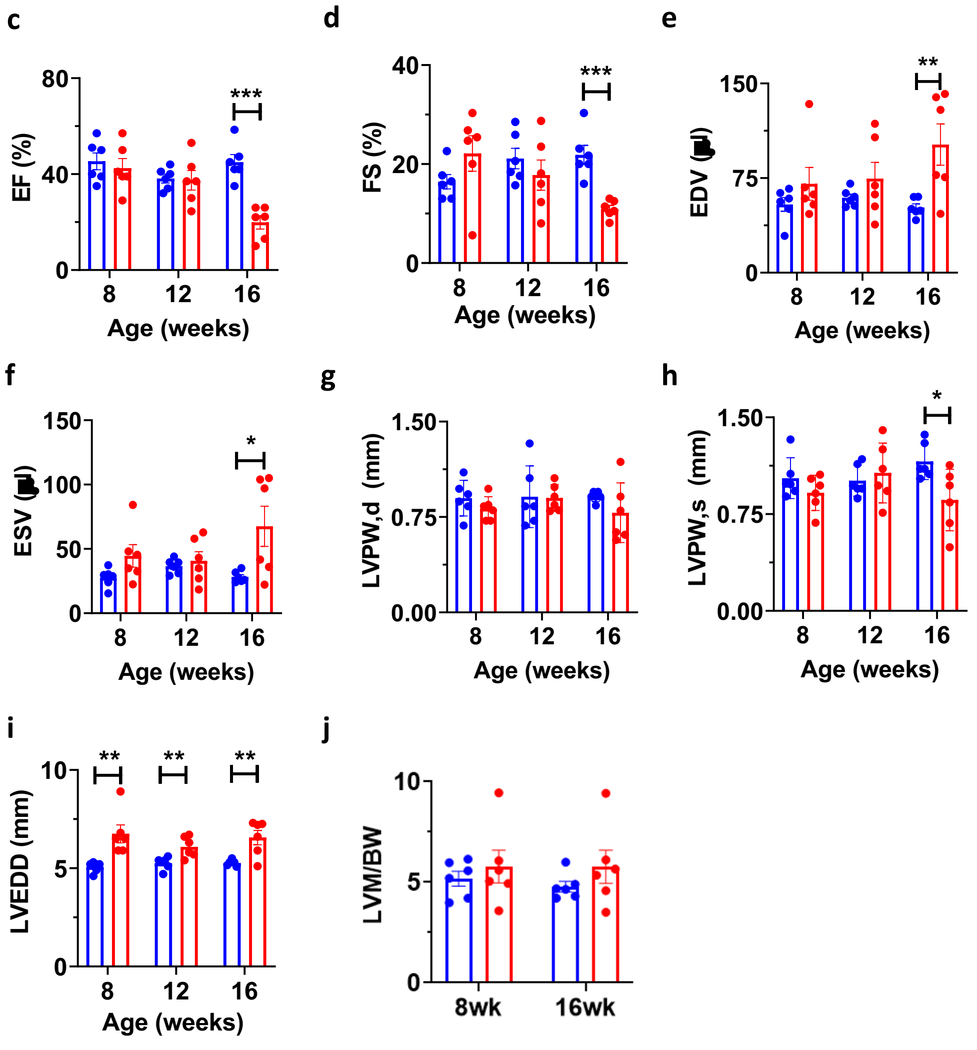

Figure1 c-i Echocardiography analysis of left ventricular ejection fraction (EF), fractional shortening (FS), left ventricular end-diastolic and end-systolic volumes (EDV and ESV), left ventricular posterior wall thickness at diastole and systole (LVPW,d and LVPW,s) and left ventricular end-diastolic diameter (LVEDD) respectively (n=6); **j** left ventricular mass to body weight ratio (LVM/BW);

p-value: two-tailed Multiple t-test performed in all comparisons. *<0.05, **<0.01 and ***<0.001.

Following the reviewer's recommendation, we are also providing a Table describing the cardiac phenotype during the entire development, including intra-utero echocardiograms performed in pregnant mice (and we also included mice that died at different time points). Our small animal echocardiographic core lab led by Dr. Kevin J. Whitehead, co-author in our revised manuscript, has extensive experience in echocardiograms performed in pregnant mice[3]. This Rebuttal Letter Ref #3 (which is Ref #16 in the revised manuscript) describes the echocardiographic methodology utilized for these analyses and was published by Dr. Navankasattusas (key member of our lab and co-author since the original submission) and Dr. Whitehead. This table has now been included in the revised manuscript as Supplemental Table 1. Please also see the main text of the revised manuscript, Page 4, Lines 100-102):

"We are also providing Supplemental Table 1 which describes the cardiac phenotype during the entire development, including intra-utero echocardiograms performed in pregnant mice[3]."

Genotype	Gender	Age	EF(%)	EDV (ul)	ESV (ul)	FS(%)	LVAW;d (mm)	LVPW;d (mm)	LVAW;s (mm)	LVPW;s (mm)	LVEDD (mm)	LVM-C (mg)
WT	F	E19	50.20	8.98	4.47	23.60	0.54	0.03	0.69	0.23	1.74	5.98
WT	F	E19	76.00	13.10	3.18	42.00	0.29	0.22	0.48	0.48	2.03	6.69
WT	M	E19	61.00	15.60	6.20	31.00	0.40	0.76	0.77	1.03	2.17	22.40
WT	M	E19	64.20	13.10	4.70	32.80	0.43	0.50	0.69	0.69	2.02	14.80
WT	M	E19	57.10	11.70	5.10	27.90	0.58	0.39	0.60	0.43	1.94	14.70
KO	F	E19	76.00	5.00	1.20	41.20	0.42	0.38	0.45	0.39	1.38	6.50
KO	F	E19	51.00	7.80	3.80	24.00	0.36	0.34	0.48	0.43	1.64	7.10
KO	F	E19	64.10	7.20	2.60	32.10	0.28	0.36	0.37	0.45	1.60	5.97
KO	M	E19	44.00	18.30	10.30	20.40	0.51	0.25	0.77	0.44	2.30	13.90
KO	M	E19	58.00	9.30	3.89	28.20	0.50	0.33	0.58	0.43	1.77	10.10
**t-test p-value**			0.670541895	0.288503361	0.82976043	0.64226969	0.619139583	0.720591878	0.203860202	0.35444948	0.207737521	0.26081477
WT	M	1 week	69.00	16.40	5.20	36.30	0.51	0.36	0.66	0.92	2.20	15.50
WT	M	1 week	80.00	9.20	1.70	46.00	0.43	0.91	0.96	1.11	1.76	20.40
WT	F	1 week	75.00	10.40	2.60	42.00	0.31	0.70	0.62	0.93	1.85	14.36
WT	M	1 week	70.00	17.60	5.30	37.10	0.58	0.95	1.01	1.42	2.26	36.20
WT	F	1 week	61.00	17.50	6.90	30.70	0.56	0.47	0.79	0.54	2.27	20.31
KO	F	1 week	83.00	18.10	3.03	47.90	0.45	0.52	0.90	1.00	2.30	19.20
KO	F	1 week	71.00	29.30	8.40	39.00	0.56	0.59	0.87	0.99	2.79	33.60
KO	M	1 week	69.00	10.60	3.20	36.50	0.73	0.80	0.98	0.94	1.86	27.20
KO	M	1 week	74.00	6.12	1.23	41.00	0.47	0.54	0.78	0.87	2.16	18.40
KO	F	1 week	74.50	6.10	1.55	40.20	0.91	0.54	1.31	0.58	1.50	18.80
**t-test p-value**			0.432821479	0.971864454	0.608569466	0.46311517	0.186892272	0.552565042	0.224284198	0.533266897	0.829175282	0.684579556
WT	F	4 week	70.80	40.30	11.70	39.00	0.80	0.77	1.40	1.10	3.18	63.10
WT	M	4 week	73.20	29.10	7.80	40.00	1.05	1.02	1.47	1.32	2.80	78.60
WT	F	4 week	52.00	43.00	21.10	25.40	0.72	0.65	0.99	0.84	3.30	54.80
WT	M	4 week	50.00	47.60	24.10	24.30	0.65	0.75	0.97	0.93	3.40	59.90
WT	M	4 week	60.00	53.00	28.00	29.00	0.99	0.64	1.40	1.00	3.80	90.60
KO	F	4 week	47.00	50.00	26.00	23.00	0.79	0.68	1.04	0.87	3.50	66.00
KO	F	4 week	45.00	94.00	51.00	22.00	0.68	0.69	1.04	0.96	4.60	95.00
KO	M	4 week	39.00	77.40	47.20	19.00	0.84	0.63	1.13	1.07	4.18	90.30
KO	M	4 week	50.00	65.50	32.10	25.40	0.56	0.75	0.84	1.07	3.90	69.30
KO	F	4 week	52.80	70.60	33.20	26.80	0.60	0.44	1.24	0.77	4.01	54.60
**t-test p-value**			**0.034852075**	**0.011940732**	**0.013989872**	0.067053407	0.156814148	0.180474805	0.181190592	0.40404127	**0.015585112**	0.592199035
WT	F	8 week	51.00	66.60	32.80	25.50	0.67	0.89	0.97	1.23	3.90	88.00
WT	M	8 week	48.00	66.00	34.90	23.60	0.67	0.95	0.99	1.13	3.92	92.80
WT	F	8 week	47.00	73.00	40.30	22.60	0.54	0.68	0.95	1.06	4.00	68.40
WT	M	8 week	63.00	43.80	16.20	33.30	0.82	1.08	1.21	1.52	3.20	88.20
WT	M	8 week	55.00	59.20	26.90	27.70	0.78	0.79	1.16	1.17	3.70	82.10
KO	M	8 week	45.90	73.40	40.00	22.60	0.80	0.93	1.16	1.15	4.10	108.80
KO	M	8 week	37.50	86.90	54.40	17.90	0.64	0.93	0.86	1.09	4.40	106.70
KO	M	8 week	32.20	80.80	54.80	15.10	0.95	0.76	1.06	0.97	4.23	113.20
KO	F	8 week	41.00	73.90	43.50	19.80	0.89	0.71	1.15	0.84	4.10	97.60
KO	F	8 week	28.10	96.50	69.40	13.10	0.75	0.74	0.80	0.99	4.60	107.40
**t-test p-value**			**0.006065393**	**0.014635108**	**0.010554216**	**0.008455039**	0.169574585	0.472063682	0.618164153	0.060682026	**0.016956878**	**0.002852524**

Supplemental Table 1: Echocardiographic data performed on WT and KO mice at different timepoints (n=5)

2. Figure 2: What is the mechanism by which VDAC2 deficiency leads to a marked decrease in the level of most proteins involved in calcium handling (SERCA2, PLN and all channels)? You

showed one continuous blot for GAPDH and VIN; however, you cut the blot in the middle for NCX and CASQ2. This is not possible. Please show continuous blot for both NCX and CASQ2.

Response 2: Thank you for this question. We have included in our revised manuscript Pages 8-9, Lines 218-234 the following:
"Mitochondria-ER/SR interactions have long been studied and it is commonly believed that this interaction is crucial for mitochondrial calcium dynamics[4,5,6]. Mitochondria are known to recognize calcium rich niches near SERCA2, RYR2 and NCX1 where major calcium transport happens[7]. We show in this study that the crosstalk of VDAC2 with both calcium release through-RYR2 and diastolic calcium removal by SERCA2 and NCX1, plays an important role in determining cellular calcium homeostasis and when this crosstalk is lost, there is a significant impairment in both mitochondrial and cytosolic calcium signaling. We also observed a significant reduction in most calcium handling proteins like RYR2, SERCA2, NCX1 and CASQ2 in VDAC2-KO mice. While these results are unexpected, they might be explained by different hypotheses: It is feasible that the lack of mitochondrial calcium uptake specifically through the loss of RYR2-VDAC2 connection results in a lack of energy demand which was shown to depend on mitochondrial calcium uptake[8]. In this scenario the basic metabolism of VDAC2-KO cells would be comparable to WT (as demonstrated in our results), while they are unable to adapt to higher workload under stress situations and might consecutively down-regulate calcium handling proteins. Alternatively, the down-regulation might be a result of compensatory mechanism to the slower rate of calcium uptake observed in the KO. Additionally, a down-regulation due to loss of structural integrity of the myocardium as observed in our experiment is feasible." Along those lines further investigation is warranted and is something that we will continue to pursue as part of our heart failure and myocardial recovery research program.

Also, following the reviewer's recommendation continuous blots for CASQ2 and NCX are shown below and also included in the manuscript (Figure 2c).

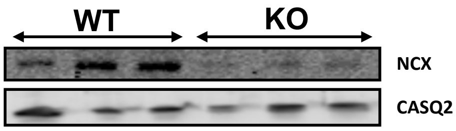

Figure 2c: Representative western blot image (n=3)

3. Previous study showed that knock-down of VDAC2 did not result in the disappearance of NCX, PLN and several other proteins. The authors should discuss these points.

Response 3: We appreciate this interesting comment. Indeed, this prior publication on VDAC2 KD in HL1 cell line did not show any significant difference in the calcium handling proteins[9]. However, several points make the comparison between the two systems difficult. i) in HL-1 cells VDAC2 was knocked down by an siRNA approach and the knock-down was incomplete (75%), ii) the authors do not indicate if selection for transduced cells was performed or a fraction of wild-type cells remain in the cultures, which could mask the results. iii) the authors indicate that "the medium was changed every day until 60h after viral infection" but do not indicate for how long after transduction cells were used for experiments and it is thus feasible that the observed down-regulation of these proteins occurs over an extended period of time, iv) though beating, HL-1 cells

do not encounter physiological triggers like adrenergic stimulation or pressure, which might lead to significantly milder effects of VDAC2 knock-down in these cells. We have also clarified this in our revised manuscript, please see Page 9, Lines 234-241.

"Also, our data stands in contrast to previous observations that knock-down of VDAC2 in HL-1cells did not alter expression levels of calcium handling proteins[9]. However, major differences between the model systems might explain these discrepancies: Partial inhibition of VDAC2 (~75%) was achieved using viral transduction and cells were cultured *in-vitro* for limited time. It is thus feasible that the down-regulation of these proteins is masked either by the remaining VDAC2 expression, the lack of triggers present *in-vivo* like pressure or adrenergic stimulation, or that these translational changes only manifest after a longer time."

4. Figure 3: The IP experiment is not convincing. I see several bands in the VDAC2 input, a major band at a lower MW in the SERCA2 input, major bands at a lower MW in the NCX input. In addition, NCX has 2 MW, one about 100 kDa and another one at 160 kDa, so it does not fit. I guess what you see with your NCX antibody is not NCX. A positive control for IP is required such as RYR2. The anti-VDAC2 antibody is not fully specific. Immunofluorescence analysis of VDAC2-SERCA2 and VDAC2-NCX should be also shown. Proximity-ligation assay could also be done to support proximity of VDAC2 with the respective protein partners.

Response 4: We appreciate this concern and suggestions by the reviewer. We repeated the co-IP experiments with WT and KO mice. The KO is used as a control to show the specificity of pull-down. The NCX antibody is designed to recognize different isoforms such as post-translational modifications or cleavage, multimers, etc. We also performed immunofluorescence and proximity ligation assay as suggested by the reviewer. All these experiments suggest interaction of VDAC2 with SERCA2 and NCX1. These results are now included in Figure 4a-c (in revised manuscript) and Page 5, Lines 132-133. "VDAC2-KO heart was used as negative control for co-IP (Figure 4a). This interaction was also confirmed by immunofluorescence and proximity ligation assays (PLA)".

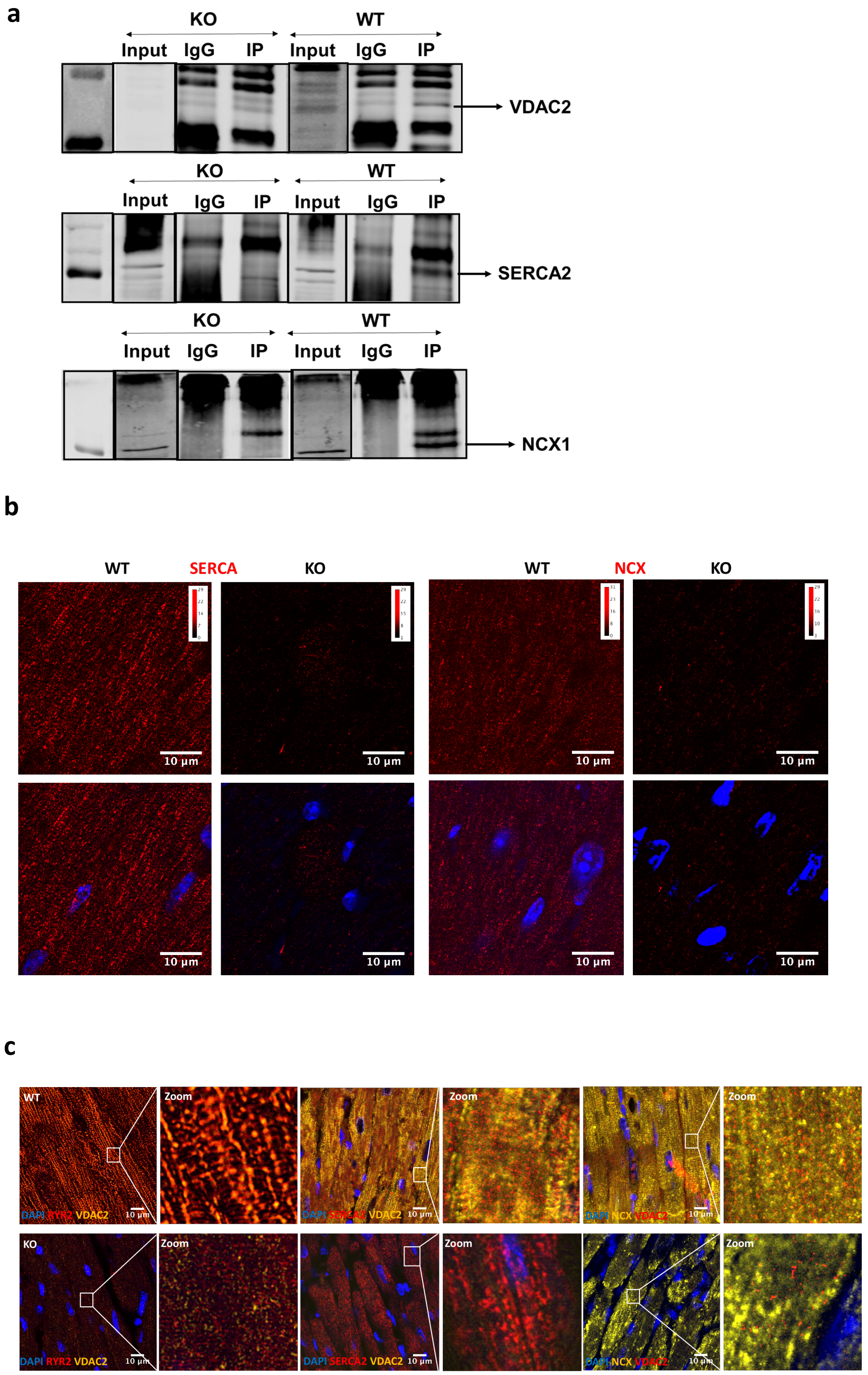

Figure 4: Cross-talk between mitochondrial and ER/SR facilitates mitochondrial calcium cycling. **a** Representative immunoprecipitation image from cardiac ventricular sample of 8-week-old mice (n=3); **b** Representative proximity ligation assay image for VDAC2-SERCA2 and VDAC2-NCX1 interaction in 8-week-old mice (Scale bar, 10μm) (n=3 mice)**;** **c** Representative immunofluorescence image from 16-week-old mice heart (Scale bar, 10μm) (n=3).

5. AP- The representative AP is not representative since APD90 looks markedly shorter in the KO, suggestive of higher heart rate. I am not convinced. More cells are required.

Response 5: We agree with the reviewer. We revisited these results with our key co-investigator and co-author in our initial submission, Dr. Kenneth W. Spitzer who is a senior leading expert in cardiac cellular electrophysiology and calcium handling. Dr. Spitzer re-assessed the consistency of our findings and helped select an AP that better reflects the mean values for APD. This has been changed in the manuscript Figure 5c.

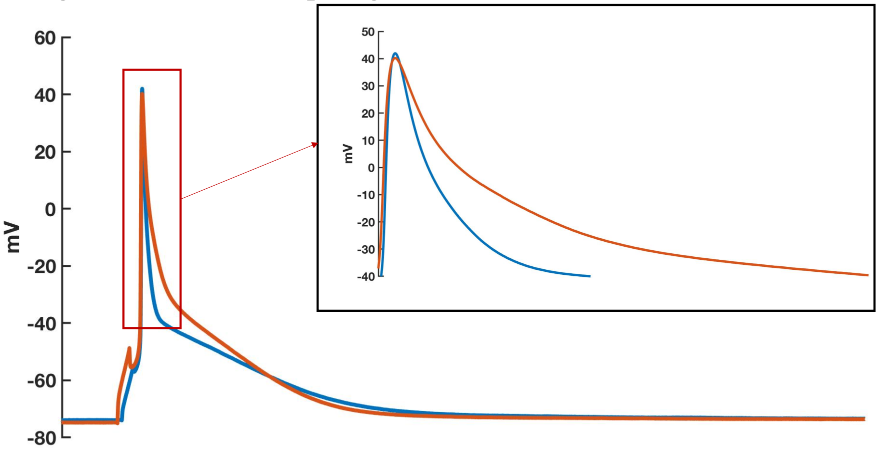

Figure 5c: Representative action potential image

6. The C57/BL6 mice are on two different backgrounds (J or N) that could markedly alter mitochondrial function and metabolism. The authors should write which background has been taken and discussed the possible implication.

Response 6: Thank you for this comment. We used a C57BL6J strain for all experiments. This information is now included in the manuscript (Page 12, Line 311). No known altered mitochondrial function have been reported in this strain. Additionally, C57Bl6J strains have been used as models for studying metabolic disorders.

7. Alpha-MHC Cre per se can induced cardiac dysfunction. The authors said that they used either flox or Cre as control, which I found not appropriate. It would be important to show echo data performed on Flox, Cre and VDAC2-KO mice to see whether the controls behave similarly.

Response 7: Thank you for this comment. Echo data shows no significant difference between the Flox and Cre mice. These results have now been included in the manuscript (Supplemental Figure 1a-g) and Page 3, Lines 89-90:
"Mice with either flox or Cre were used as WT and echocardiography data on flox or Cre only mice shows no significant differences (Supplemental Figure 1a-g)."

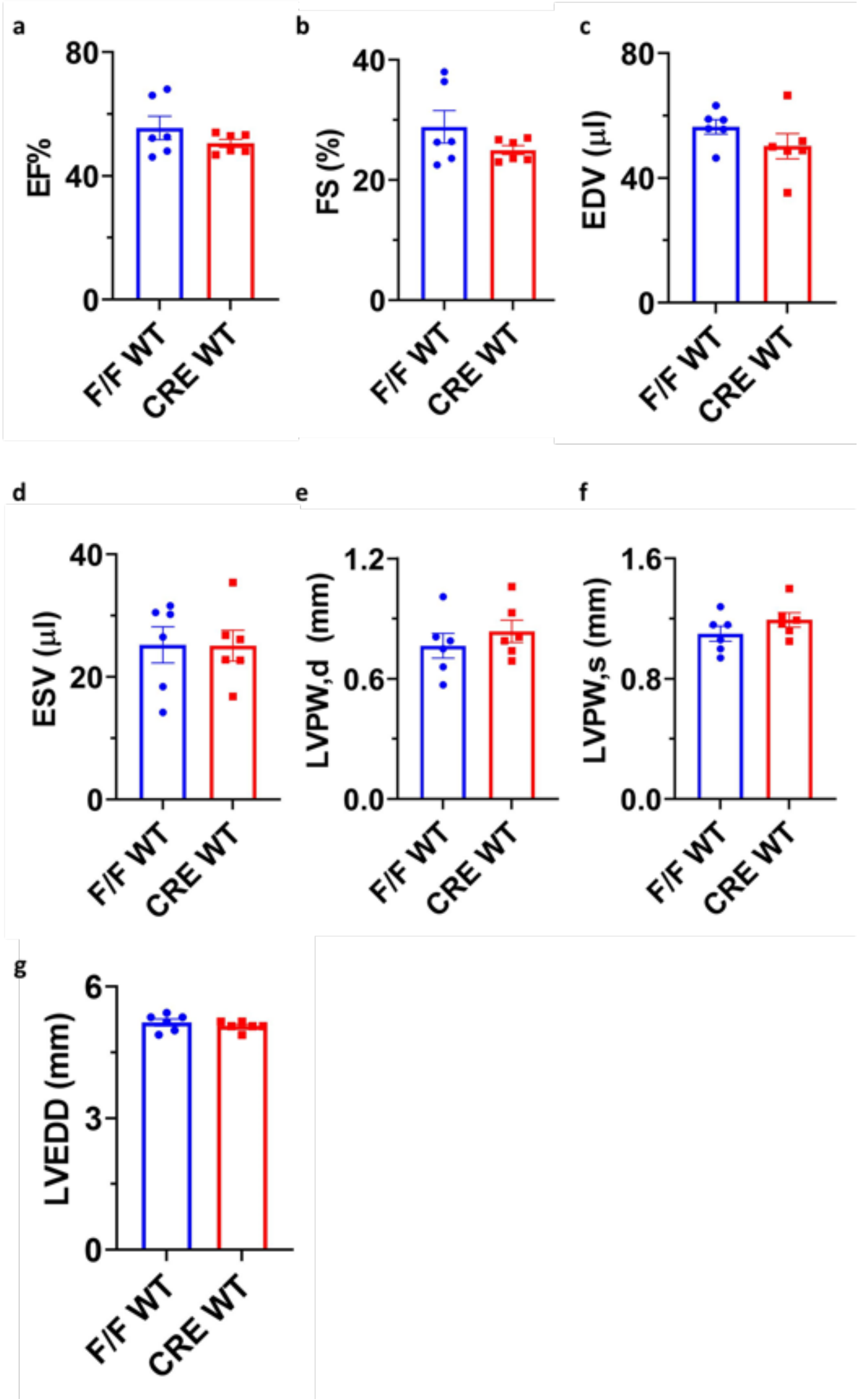

Supplemental Figure 1: No significant difference between flox-only and Cre-only mice. a-g Echocardiography analysis of left ventricular ejection fraction (EF), fractional shortening (FS), left ventricular end-diastolic and end-systolic volumes (EDV and ESV), left-ventricular posterior wall thickness at diastole and systole (LVPW,d and LVPW,s) and left ventricular end diastolic diameter (LVEDD) in 16-week-old mice respectively, (n=6). Unpaired t-test performed.

8. Most importantly, the authors should test whether efsevin in wild-type mice induced the same phenotype as in KO mice.

Response 8: We thank the reviewer for bringing our attention to efsevin. Upon this comment we have collaborated with Dr. Ohyun Kwon at UCLA and Dr. Johann Schredelseker at LMU Munich, who have extensive research experience with efsevin. Dr. Kwon (now co-author in our revised manuscript) synthesized efsevin and provided the required quantities to the lab of her long-time collaborator Dr. Schredelseker to perform the requested experiments. Dr. Schredelseker's lab went ahead and tested efsevin on wild-type cardiomyocytes and found an increase in calcium transient amplitude together with an acceleration of cytosolic calcium removal. Since efsevin is an enhancer of mitochondrial calcium uptake[10], this data is in line with the results from our KO, which shows reduced calcium transients and decelerated calcium removal upon VDAC2 knockout. These results are now included in Supplemental Figure 3a-c.
Please see revised manuscript, Page 5, Lines 124-128:
"To investigate if the decreased calcium transients are directly attributable to the lack of VDAC2 we tested effects of VDAC2 agonist efsevin[10] on calcium transients in WT cardiomyocytes and found the opposite effect, namely an increase in calcium transient amplitude and an acceleration of the decay phase (Supplemental Figure 3a-c)."

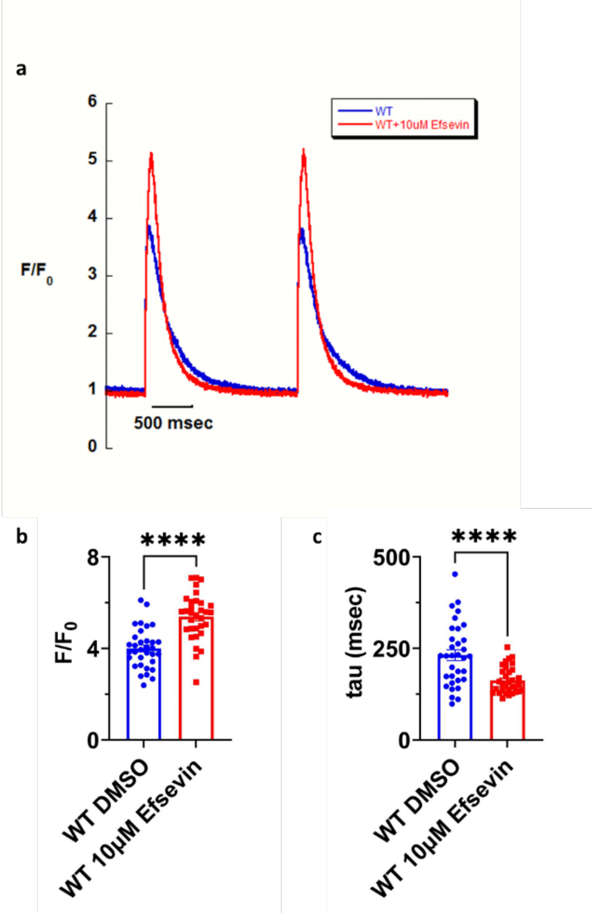

Supplemental Figure 3: **a** Representative calcium transient; **b** Amplitude (F/F_0); **c** Rate of calcium uptake (tau)(msec) (N=3, WT n=33, WT+10μM Efsevin n=32). Pacing=0.5Hz
p-value: unpaired t-test performed in all comparisons. *<0.05, **<0.01 and ***<0.001.

Dr. Schredelseker's lab then also tested effects of efsevin on the contractile force in murine myocardium. Efsevin enhanced contractile force and accelerated myocardial relaxation in these preparations. In a translational approach, we then used a murine model for pressure-overload induced heart failure (TAC) and again found enhanced contractile force upon efsevin treatment, highlighting the potential of VDAC2 as a pharmacological target structure in heart failure. This data is now included in the manuscript in Figure 9a-b and discussed in Pages 7-8, Lines 195-204: "We show that VDAC2 regulates cardiac calcium signaling without having major effects on bioenergetics and VDAC2 deletion causes DCM, making it a candidate structure for HF therapy. We therefore addressed the question if increasing VDAC2s calcium uptake activity could enhance cardiac contractile force in HF. Therefore, we used the established model of pressure overload induced HF by transverse aortic constriction (TAC). Since administration of efsevin to mice is difficult for more than a few days due to its short plasma half-life time and efsevin is not orally bioavailable, we took advantage of organotypic heart slices and investigated the effect of efsevin on myocardial contractile strength. Strikingly, efsevin enhanced contractile strength and accelerated relaxation in both control and failing hearts (Figure 9a-b)."

We once again thank the reviewer for bringing up efsevin and believe that the addition of this translational data strengthens the manuscript. For this work Drs. Schredelseker and Dendorfer as well as Kira Steinhorst and Paulina Sander are now listed as a co-authors in the revised version.

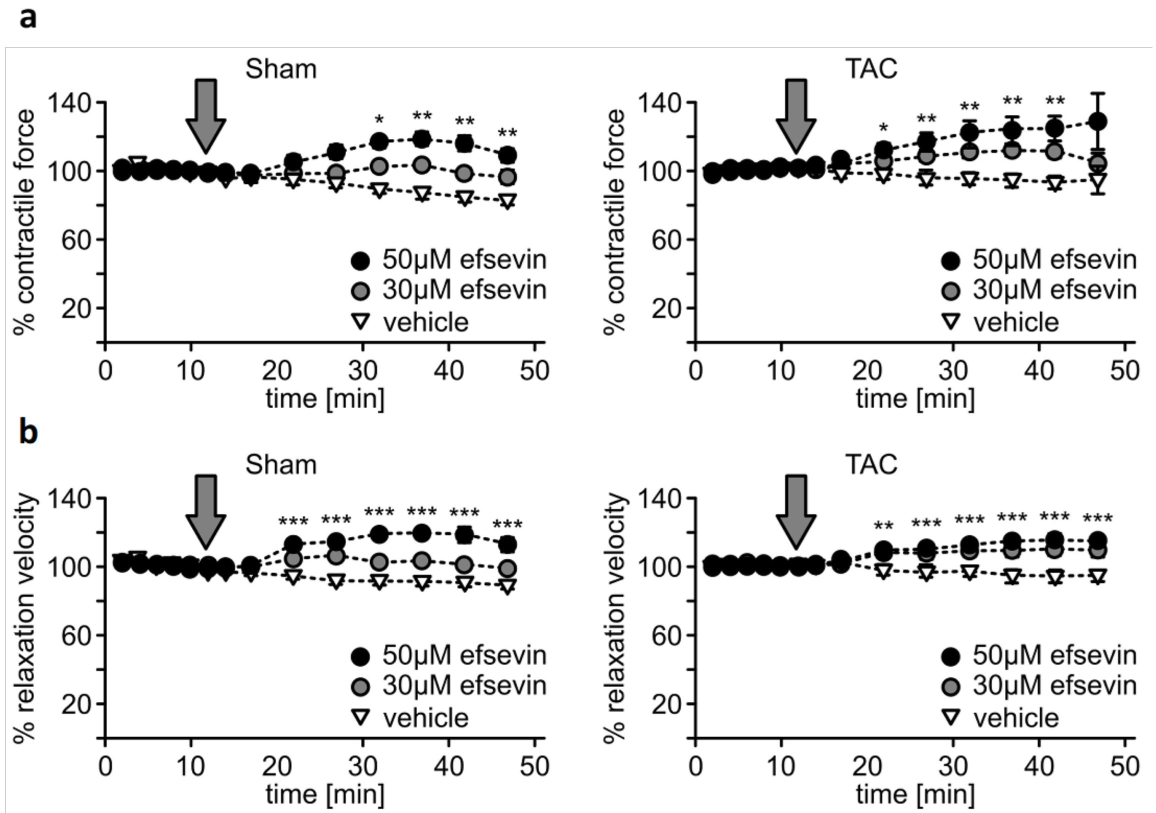

Figure 9: Enhanced contractile force and accelerated relaxation speed in myocardial tissue upon VDAC2 activation. a Measurement of relative contractile force in organotypic myocardial slices from both Sham- (left panel) and TAC-operated mice (right panel) after superfusion with either 30 or 50 μM of the VDAC2 agonist efsevin. Each dot represents quantification of twitch amplitudes over 20sec at indicated time points. The gray arrow indicates the start of efsevin

superfusion. **b** Relaxation speed of twitches of myocardial slices shown (N=8 mice, n=22 slices for TAC, N=6 mice, n=14 slices for Sham)
p-value: unpaired t-test performed in all comparisons. *<0.05, **<0.01 and ***<0.001.

Minor comments:

9. Replace protein expression by protein level. Figure 1a – please give a darker blot for VDAC2 in KO mice.

Response 9: Thank you for the suggestion. The blot has been replaced with a darker blot.

10. Figure 1b: how can you do statistics with no VDAC2 in KO mice? Anything compared to 0 is not possible. Why did you get any values in KO mice?

Response 10: We appreciate this question. The graphs show a relative difference between the two groups. However, we do observe background signal in VDAC2 KO mice (non-zero). This might be signal from fibroblasts, smooth muscle cells or other cell types since we only have a cardiomyocyte-specific deletion of VDAC2[11,12].

11. Figure 1k-m: how many mice were used. Please add the N7n (number of mice / number of cells) analyzed.

Response 11: N=3 mice and n=42 cells were analyzed. This has now been included in Figure 1 legend. This is now Figure 1n-p in the revised manuscript.

12. Figure 2: what is VIN as an endogenous loading control?

Response 12: Vinculin has been used as a loading control in a number of publications[13,14]. Additionally, we also don't observe a significant difference between GAPDH and VINCULIN between WT and KO. This has been included in the manuscript (Supplemental Figure 4p). We have indicated this in the revised manuscript Pages 6-7, Lines 173-174: "VINCULIN and GAPDH were used as lane-loading controls and we observed no difference in these protein levels between WT and KO".

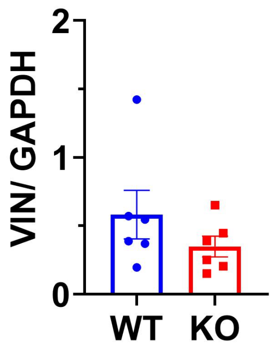

Supplemental Figure 4p: Western blot quantification data using Image Studio Lite (version5.2.5) (n=6). Unpaired t-test performed.

13. Why did you use a miRNeasy Mini kit to extract total RNA?

Response 13: We used miRNeasy Mini kit to purify both smaller and larger mRNA.

Reviewer #3 (Remarks to the Author):

This submission is an interesting, well-designed study whose premise is that cardiomyocyte-specific developmental deletion of Vdac2 in a mouse model contributes to a pathological phenotype described in the paper by severe impairment in excitation-contraction coupling, altered calcium signaling in the cytosol and mitochondria, and adverse cardiac remodeling (fibrosis) which increased mortality in the KO mouse model. The supporting evidence for this paper's premise, namely the importance of VDAC2 in cardiac physiology, is described in previous work by this group as well as others in the field. Overall, the methods described in this submission are robust and support the hypothesis, although some clarification and additional data presentation would increase the interpretability and impact.

Some specific issues, comments and suggestions this reviewer has for the submission are as follows:
1. The authors do not indicate which timepoint is being compared in the echocardiography data represented in Fig.1d-g. The manuscript claims that serial echo was performed from E17 until 16 weeks postpartum, but the authors need to indicate at what age the data in these panels are representing, as cardiac function does not seem to deteriorate until ~5w of age postnatally per Fig.1c.

Response 1: We appreciate this recommendation. As rightly pointed out by the reviewer, we performed serial echocardiography on mice until 16-weeks of age. As shown in Figure 1b (in the revised manuscript), we observed variable mortality (from 5-weeks of age up to 20-weeks of age). Since majority of the mice died during 18-20 weeks, the data shown below are from mice that survived until that time point (for statistical comparison reasons). Progressive deterioration of cardiac function was observed in these mice and a significant difference between the two groups (WT and KO) was observed at 16-weeks of age. We have now included these longitudinal data in Figure 1c-j (in the revised manuscript).

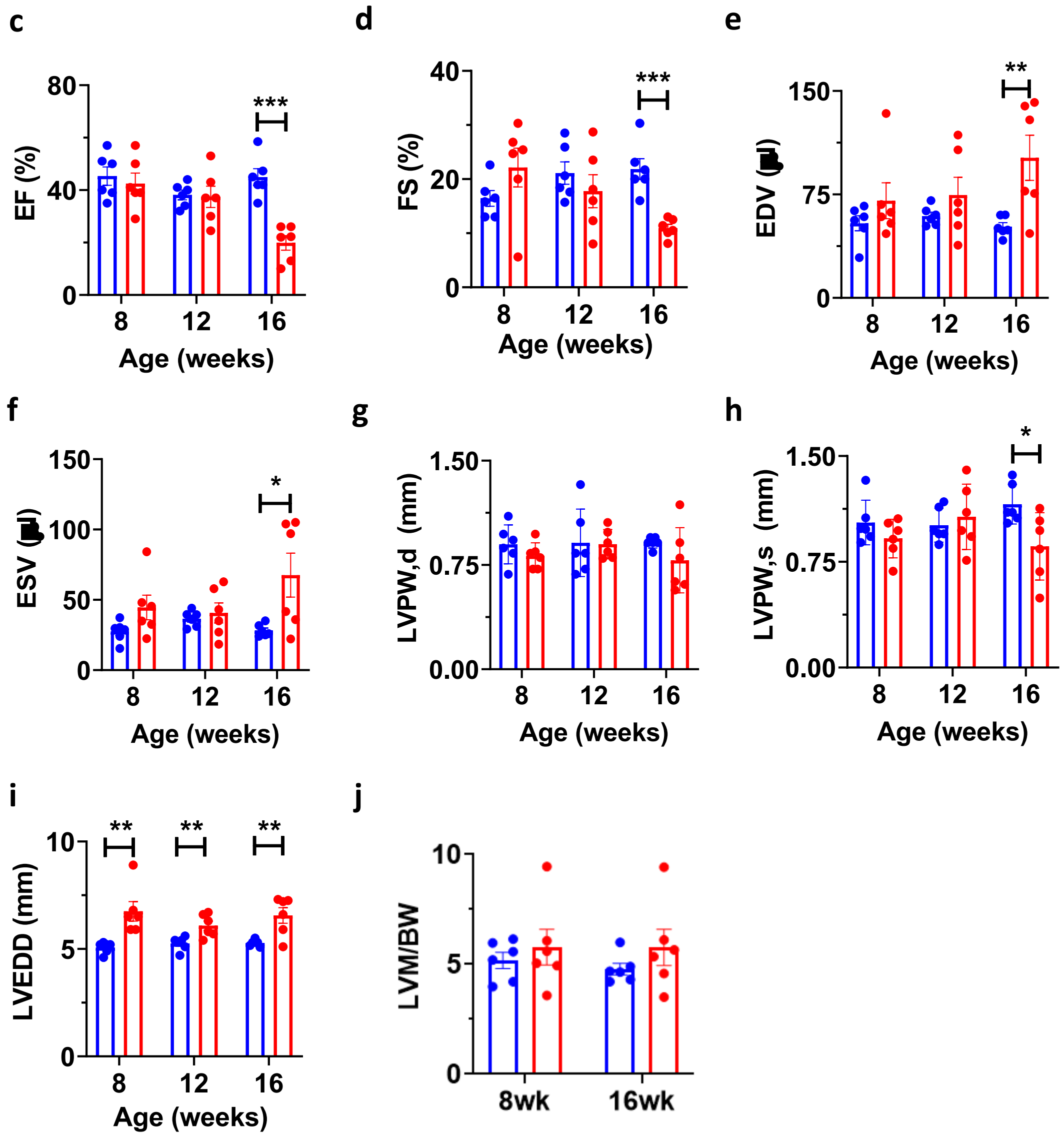

Figure1 c-i Echocardiography analysis of left ventricular ejection fraction (EF), fractional shortening (FS), left ventricular end-diastolic and end-systolic volumes (EDV and ESV), left ventricular posterior wall thickness at diastole and systole (LVPW,d and LVPW,s) and left ventricular end-diastolic diameter (LVEDD) respectively (n=6); **j** left ventricular mass to body weight ratio (LVM/BW);
p-value: two-tailed Multiple t-test performed in all comparisons. *<0.05, **<0.01 and ***<0.001.

Following the reviewer's recommendation, we are also providing a Table describing the cardiac phenotype during the entire development, including intra-utero echocardiograms performed in pregnant mice (and we also included mice that died at different time points). Our small animal echocardiographic core lab led by Dr. Kevin J. Whitehead, co-author in our revised manuscript, has extensive experience in echocardiograms performed in pregnant mice[3]. This Rebuttal Letter Ref #3 (which is Ref #16 in the revised manuscript) describes the echocardiographic methodology utilized for these analyses and was published by Dr Navankasattusas (key member of our lab and co-author since the original submission) and Dr. Whitehead. This table has now been included in

the revised manuscript as Supplemental Table 1. Please also see the main text of the revised manuscript, Page 4, Lines 100-102):

"We are also providing Supplemental Table 1 which describes the cardiac phenotype during the entire development, including intra-utero echocardiograms performed in pregnant mice."

Geno	Gender	Age	EF(%)	EDV (ul)	ESV (ul)	FS(%)	LVAW;d (mm)	LVPW;d (mm)	LVAW;s (mm)	LVPW;s (mm)	LVEDD (mm)	LVM-C (mg)
WT	F	E19	50.20	8.98	4.47	23.60	0.54	0.03	0.69	0.23	1.74	5.98
WT	F	E19	76.00	13.10	3.18	42.00	0.29	0.22	0.48	0.48	2.03	6.69
WT	M	E19	61.00	15.60	6.20	31.00	0.40	0.76	0.77	1.03	2.17	22.40
WT	M	E19	64.20	13.10	4.70	32.80	0.43	0.50	0.69	0.69	2.02	14.80
WT	M	E19	57.10	11.70	5.10	27.90	0.58	0.39	0.60	0.43	1.94	14.70
KO	F	E19	76.00	5.00	1.20	41.20	0.42	0.38	0.45	0.39	1.38	6.50
KO	F	E19	51.00	7.80	3.80	24.00	0.36	0.34	0.48	0.43	1.64	7.10
KO	F	E19	64.10	7.20	2.60	32.10	0.28	0.36	0.37	0.45	1.60	5.97
KO	M	E19	44.00	18.30	10.30	20.40	0.51	0.25	0.77	0.44	2.30	13.90
KO	M	E19	58.00	9.30	3.89	28.20	0.50	0.33	0.58	0.43	1.77	10.10
t-test p-value			0.670541895	0.288503361	0.82976043	0.64226969	0.619139583	0.720591878	0.203860202	0.35444948	0.207737521	0.26081477
WT	M	1 week	69.00	16.40	5.20	36.30	0.51	0.36	0.66	0.92	2.20	15.50
WT	M	1 week	80.00	9.20	1.70	46.00	0.43	0.91	0.96	1.11	1.76	20.40
WT	F	1 week	75.00	10.40	2.60	42.00	0.31	0.70	0.62	0.93	1.85	14.36
WT	M	1 week	70.00	17.60	5.30	37.10	0.58	0.95	1.01	1.42	2.26	36.20
WT	F	1 week	61.00	17.50	6.90	30.70	0.56	0.47	0.79	0.54	2.27	20.31
KO	F	1 week	83.00	18.10	3.03	47.90	0.45	0.52	0.90	1.00	2.30	19.20
KO	F	1 week	71.00	29.30	8.40	39.00	0.56	0.59	0.87	0.99	2.79	33.60
KO	M	1 week	69.00	10.60	3.20	36.50	0.73	0.80	0.98	0.94	1.86	27.20
KO	M	1 week	74.00	6.12	1.23	41.00	0.47	0.54	0.78	0.87	2.16	18.40
KO	F	1 week	74.50	6.10	1.55	40.20	0.91	0.54	1.31	0.58	1.50	18.80
t-test p-value			0.432821479	0.971864454	0.608569466	0.46311517	0.186892272	0.552565042	0.224284198	0.533266897	0.829175282	0.684579556
WT	F	4 week	70.80	40.30	11.70	39.00	0.80	0.77	1.40	1.10	3.18	63.10
WT	M	4 week	73.20	29.10	7.80	40.00	1.05	1.02	1.47	1.32	2.80	78.60
WT	F	4 week	52.00	43.00	21.10	25.40	0.72	0.65	0.99	0.84	3.30	54.80
WT	M	4 week	50.00	47.60	24.10	24.30	0.65	0.75	0.97	0.93	3.40	59.90
WT	M	4 week	60.00	53.00	28.00	29.00	0.99	0.64	1.40	1.00	3.80	90.60
KO	F	4 week	47.00	50.00	26.00	23.00	0.79	0.68	1.04	0.87	3.50	66.00
KO	F	4 week	45.00	94.00	51.00	22.00	0.68	0.69	1.04	0.96	4.60	95.00
KO	M	4 week	39.00	77.40	47.20	19.00	0.84	0.63	1.13	1.07	4.18	90.30
KO	M	4 week	50.00	65.50	32.10	25.40	0.56	0.75	0.84	1.07	3.90	69.30
KO	F	4 week	52.80	70.60	33.20	26.80	0.60	0.44	1.24	0.77	4.01	54.60
t-test p-value			**0.034852075**	**0.011940732**	**0.013989872**	0.067053407	0.156814148	0.180474805	0.181190592	0.40404127	**0.015585112**	0.592199035
WT	F	8 week	51.00	66.60	32.80	25.50	0.67	0.89	0.97	1.23	3.90	88.00
WT	M	8 week	48.00	66.00	34.90	23.60	0.67	0.95	0.99	1.13	3.92	92.80
WT	F	8 week	47.00	73.00	40.30	22.60	0.54	0.68	0.95	1.06	4.00	68.40
WT	M	8 week	63.00	43.80	16.20	33.30	0.82	1.08	1.21	1.52	3.20	88.20
WT	M	8 week	55.00	59.20	26.90	27.70	0.78	0.79	1.16	1.17	3.70	82.10
KO	M	8 week	45.90	73.40	40.00	22.60	0.80	0.93	1.16	1.15	4.10	108.80
KO	M	8 week	37.50	86.90	54.40	17.90	0.64	0.93	0.86	1.09	4.40	106.70
KO	M	8 week	32.20	80.80	54.80	15.10	0.95	0.76	1.06	0.97	4.23	113.20
KO	F	8 week	41.00	73.90	43.50	19.80	0.89	0.71	1.15	0.84	4.10	97.60
KO	F	8 week	28.10	96.50	69.40	13.10	0.75	0.74	0.80	0.99	4.60	107.40
t-test p-value			**0.006065393**	**0.014635108**	**0.010554216**	**0.008455039**	0.169574585	0.472063682	0.618164153	0.060682026	**0.016956878**	**0.002852524**

Supplemental Table 1: Echocardiographic data performed on WT and KO mice at different timepoints (n=5). Unpaired t-test performed.

2. Additionally, the authors claim "Increased mortality was observed in the KO mice between 5-20 weeks postpartum. Most of these events occurred during 20 weeks postpartum." It is worth noting that these are not conditional KO mice, where Vdac2 is turned off at multiple embryonic time points (eg, formation of the FHF/SHF). Hearts represented in Fig.1i is a comparison of KO to WT at what age? 16w (If same as the echo data in Fig.1h), but the authors' interpretation of the findings insinuate that fetally and at birth, Vdac2KO hearts are phenotypically and functionally identical to WT hearts. This does not indicate a developmental role for Vdac2 in the murine heart, but rather a functional role postnatally, whose loss has a detrimental effect on cardiac myocytes over time. Reviewer would caution against using the term "developmental importance" versus "functional importance."

Response 2: Thank you for the clarification. As correctly pointed out by the reviewers, these are not conditional KO mice. We used α-MHC promoter to drive Cre expression. We expect to see a complete KO around 8-days postnatal[8,15]. Hearts represented in Fig. 1i (Fig 1k in revised version) is from 16-week-old mice. We also agree with the reviewer on the term "functional importance". As mentioned by the reviewer, VDAC2 deletion in cardiomyocytes have a functional role postnatally than embryonically. This point has been revised throughout the manuscript.

3. Additionally, gene expression array was performed at 16w postpartum. I am curious if the authors performed any gene or protein expression analysis at earlier time points, such as in utero or acutely post-natally. While the data are convincing that "Collectively, these results suggest that loss of VDAC2 leads to abnormalities in the calcium signaling pathways" if the authors are to suggest a developmental role for VDAC2, only checking related Ca2+ handling proteins once an observable phenotype is attained does not give a comparable baseline of these critical CICR proteins.

Response 3: We appreciate this question. We performed RNA sequencing on embryos at age E18.5-E20 and observed no significant differences in the genes involved in calcium handling pathways (SERCA2, NCX1, RYR2, PLN, etc.) as shown below in Figure a. This suggests that the effects we see in adult mice are due to adaptation/maladaptation post-natally. We observed significant downregulation in genes involved in metabolism like Mthfd2, Pfkfb and Gpd1in the KO as shown below in Figure b-c. Additionally, a complete KO of VDAC2 is only observed during post-natal day 8 and hence in-utero experiments might not give a complete story behind VDAC2 deletion[15,8].

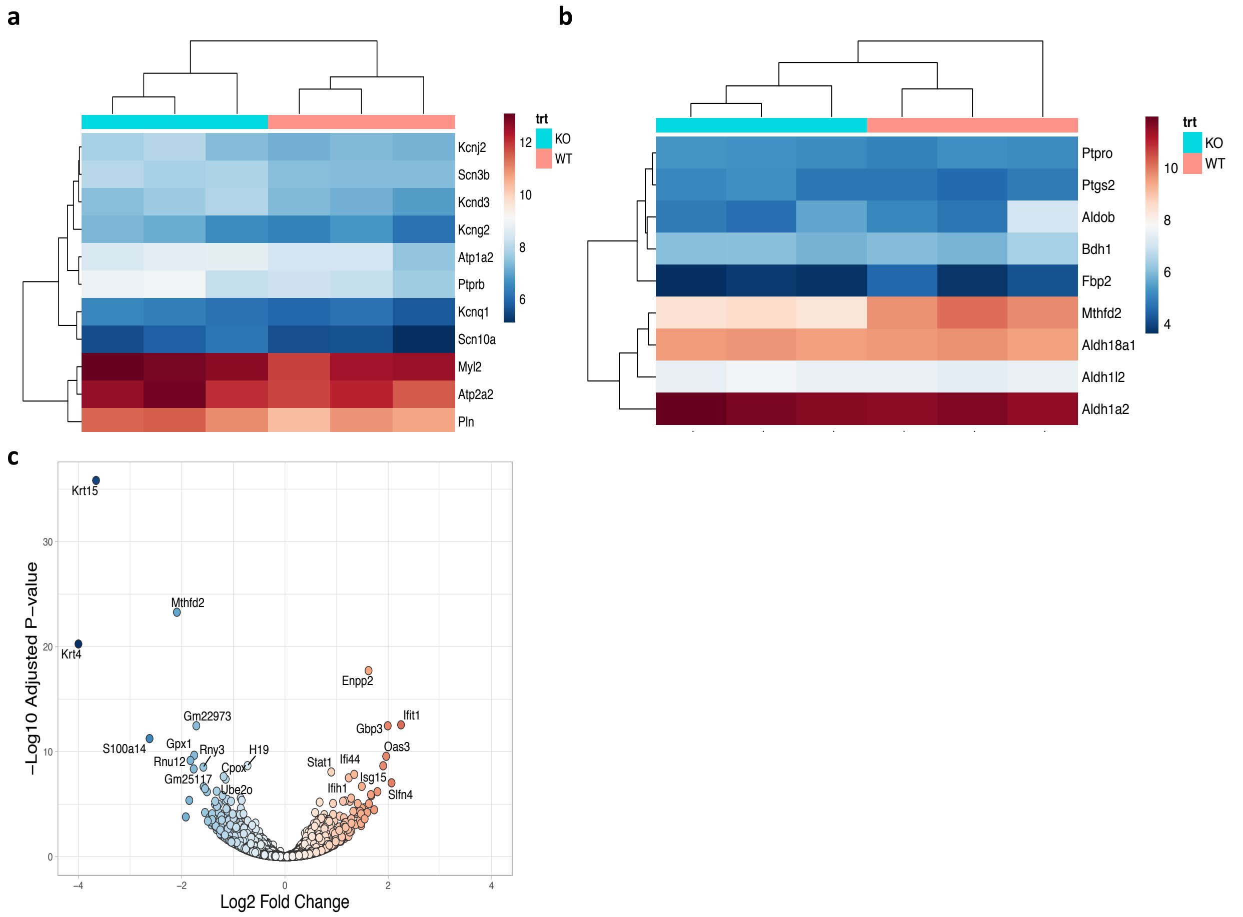

Figure a-b Heat map from RNA sequencing on E18.5-E20 embryo hearts (n=3); **c** Volcano plot (n=3)

4. It is demonstrated that SERCA2, NCX, total and phosphorylated phospholamban (PLN), and calsequestrin (CASQ2, the SR calcium binding protein) were decreased in the KO, but did the authors check for changes in RyR expression?

Response 4: Thank you for this question. We have measured RyR2 protein levels and observed a significant decrease in the KO mice. These results are now included in Figure 2c and j. Please see Page 4, Lines 117-119 in the revised manuscript: "Proteins involved in ECC such as RYR2, SERCA2, NCX1, total and phosphorylated phospholamban (PLN), and calsequestrin (CASQ2, the SR calcium binding protein) were significantly decreased in the KO".

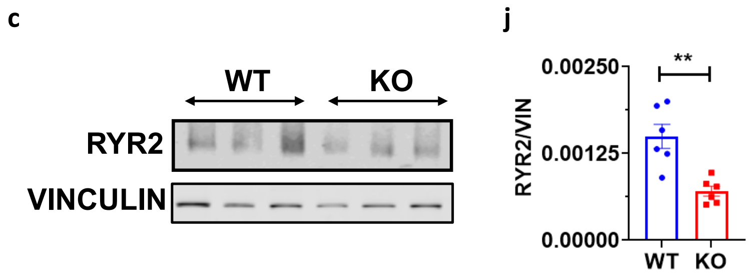

Figure 2: c Representative western blot image and lane loading control (n=3); **j** Western blot quantification data using Image Studio Lite (version 5.2.5) (n=3).
p-value: unpaired t-test performed in all comparisons. **<0.01

5. In Fig3h, why is the band lower in the input than in the IP lane in the VDAC2-NCX co-IP? Additionally, the IP lane contains a doublet whereas the input lysate for NCX is a singlet band. Could the authors explain these discrepancies between the IP pull-down and non-IPed endogenous protein?

Response 5: Thank you for the opportunity to clarify. The NCX antibody we used recognizes NCX and other isoforms of NCX (post-translation modification, multimers, etc). This explains the 2 bands. We have also observed 2 bands in some non-IPed samples before. Additionally, we have now included VDAC2-KO heart samples as control to show the specificity of interaction. This is now included in Figure 4a.

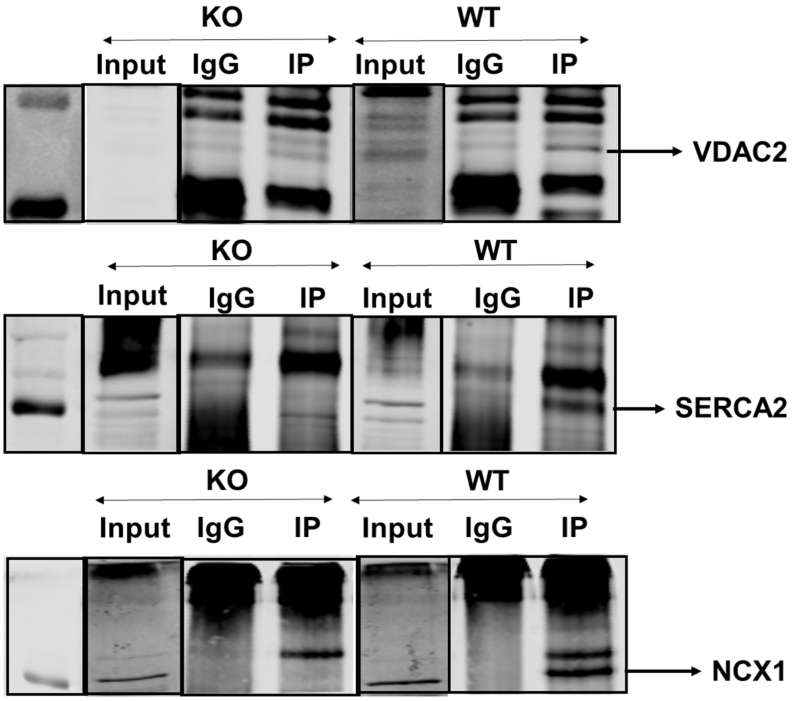

Figure 4: Cross-talk between mitochondrial and ER/SR facilitates mitochondrial calcium cycling. a Representative immunoprecipitation image from cardiac ventricular sample of 8-week-old mice (n=3);

6. Fig3.i – the WT HR measurements have no observable variance between animals. These numbers seem too good to be true, based on experience, and reviewer is curious why all the animals have identical HRs (3a through 3e are very well-done however, as is the TEM in Fig.4).

Response 6: Thank you for noticing this error. This was clearly our oversight while creating the figures. This has been corrected in Figure 5a.

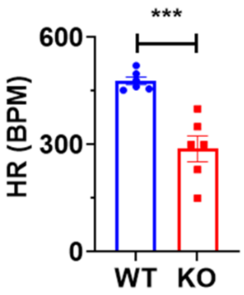

Figure 5a: Heart rate (BPM) (n=6).
p-value: unpaired t-test performed in all comparisons. ***<0.001.

7. The volcano plot from the RNAseq data are interesting, particularly the observation of an overall downregulation of enzyme levels involved in glycolysis, TCA cycle, and glycolytic pathways. However, no significant difference was observed in FAO pathways. Given that FAO is the primary fuel source for adult cardiac myocytes, this may actually have more developmental consequences in utero and acutely postnatally. What are the implications in the adult heart? Are there changes in substrate utilization in genetic DCM hearts (human or murine)? Is this reduction in glycolytic pathway components observed during fetal cardiac development?

Response 7: Thank you for this question. We performed RNA sequencing on E18.5-E20 mice and observed significant downregulation in genes involved in metabolism like Mthfd2, Pfkfb and Gpd1in the KO as shown in Figure a-b. However, a complete knock out of VDAC2 is observed only 8 days post-natally and hence we do not see striking differences as observed in the adult KO mice[13,14].

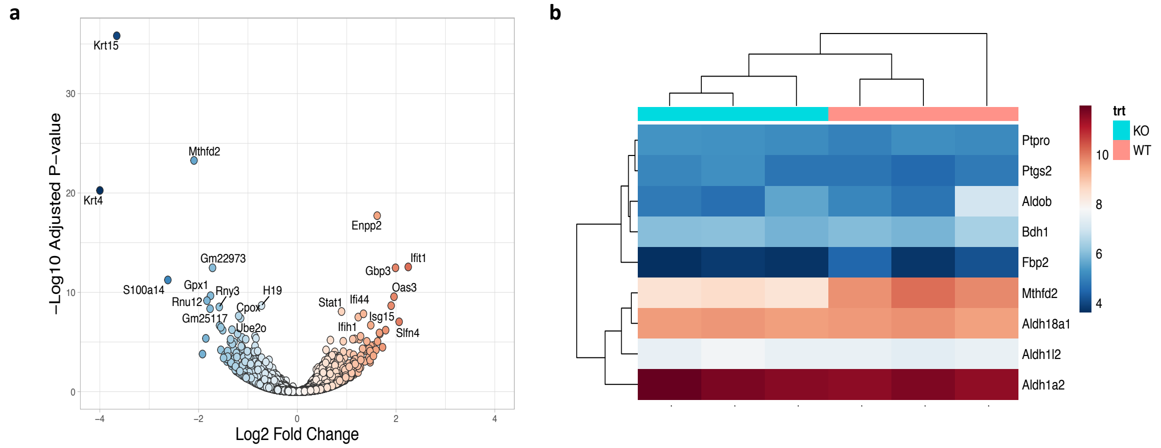

Figure a Volcano plot (n=3); **b** Heat map from RNA sequencing on E18.5-E20 embryo hearts (n=3)

Substrate utilization is known to be affected in human and animal models of DCM and HF. Increase in glucose oxidation is observed during initial stages of cardiomyopathy and progresses to a decline in glucose oxidation in end-stage HF patients[16]. It is believed that this increase in glucose oxidation during initial stages of cardiomyopathy/HF acts as a cardio-protective mechanism thus slowing down HF progression. However, the heart decompensates over a period of time and results in reduced glucose oxidation in advanced HF patients. Genetic mice models of DCM also show differences in substrate utilization. Muscle-specific deletion of mTOR has shown to cause DCM in mice model and cardiac muscle of the KO mice showed reduced FA oxidation and glycolysis related gene when compared to wild-type mice[17]. Similarly, E2F6 transgenic mice exhibiting DCM phenotype showed impaired glycolysis and increased ketone metabolism[18].

General comments:

1. This paper would be much stronger with a rescue of the KO model utilized. This is easily achievable by the senior author's group; AAV9-targeted VDAC2 expression and subsequent analysis of the phenotype – which can be performed before and after the decline in cardiac function as seen in the echo data – would have increased the impact of this paper. Likewise, the reviewer is curious whether the authors have considered an inducible VDAC2-KO model, particularly with the mention of developmental importance of this protein in calcium handling. Comparing in utero (fetal) to postnatal loss of VDAC2 would provide a wealth of information which may explain the changes in expression and activity of related proteins, particularly the ion channels. However, these are not suggestions which preclude publication for an otherwise compelling and interesting paper.

Response 1: We thank the reviewer for these suggestions. We were able to perform retro-orbital injections on WT and KO mice using AAV9-αMHC-VDAC2-GFP vector and control AAV9-αMHC-GFP vector. We injected the mice at 6-weeks of age and performed serial echocardiography until 16-weeks of age to assess any difference between control and AAV9-αMHC-VDAC2-GFP injected groups. We assessed the expression of VDAC2 using western blot and observed a significant increase in VDAC2 levels between KO GFP and KO VDAC2-GFP. Serial echocardiography showed a partial rescue of the phenotype in KO mice injected with VDAC2 vector. Significant difference was observed in left ventricular ejection fraction, end-diastolic diameter ($p=0.07$) and volumes between KO-GFP and KO receiving VDAC2-GFP. However, the reintroduction of VDAC2 in the KO mice did not fully recover the DCM phenotype. This suggests a possible role for VDAC2 as a therapeutic target. These results are now included in the manuscript (Figure 8).
Please see Page 3, Line 78-80; Page 7, Line 186-194 and Page 8, Line 215-217.

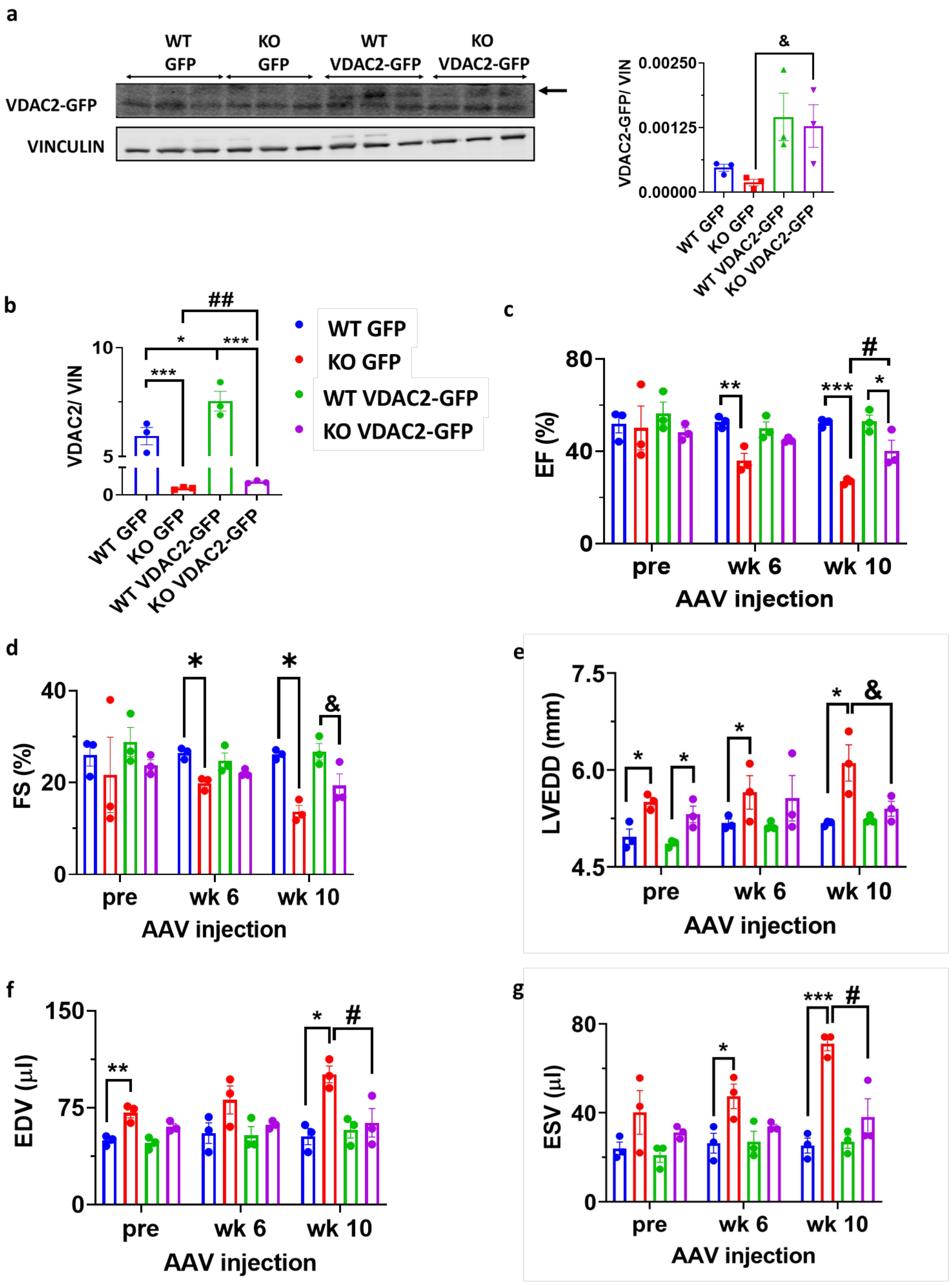

Figure 8: Partial rescue of HF phenotype upon VDAC2 reintroduction. a Representative western blot image and quantification of VDAC2-GFP fusion protein using Image Studio Lite (Version5.2.5) (n=3); **b** qRT PCR (n=3); **c-g** Serial echo cardiography parameter including ejection fraction (EF), fractional shortening (FS), left ventricular end diastolic diameter (LVEDD), end diastolic and end systolic volumes (EDV and ESV) respectively.

p-value: unpaired t-test performed in all comparisons between WT and KO. Multiple t-test performed for echocardiographic data. *,# <0.05, **,## <0.01 and ***<0.001. * = comparison between WT and KO, # = comparison between KO GFP and KO AAV. &= 0.05<p<0.08

References

1 Cheng, E. H., Sheiko, T. V., Fisher, J. K., Craigen, W. J. & Korsmeyer, S. J. VDAC2 inhibits BAK activation and mitochondrial apoptosis. *Science* **301**, 513-517, doi:10.1126/science.1083995 (2003).

2 Roy, S. S., Ehrlich, A. M., Craigen, W. J. & Hajnoczky, G. VDAC2 is required for truncated BID-induced mitochondrial apoptosis by recruiting BAK to the mitochondria. *EMBO Rep* **10**, 1341-1347, doi:10.1038/embor.2009.219 (2009).

3 Navankasattusas, S. *et al.* The netrin receptor UNC5B promotes angiogenesis in specific vascular beds. *Development* **135**, 659-667, doi:10.1242/dev.013623 (2008).

4 Eisner, V., Csordas, G. & Hajnoczky, G. Interactions between sarco-endoplasmic reticulum and mitochondria in cardiac and skeletal muscle - pivotal roles in Ca(2)(+) and reactive oxygen species signaling. *J Cell Sci* **126**, 2965-2978, doi:10.1242/jcs.093609 (2013).

5 Sharma, V. K., Ramesh, V., Franzini-Armstrong, C. & Sheu, S. S. Transport of Ca2+ from sarcoplasmic reticulum to mitochondria in rat ventricular myocytes. *J Bioenerg Biomembr* **32**, 97-104, doi:10.1023/a:1005520714221 (2000).

6 Sander, P., Gudermann, T. & Schredelseker, J. A Calcium Guard in the Outer Membrane: Is VDAC a Regulated Gatekeeper of Mitochondrial Calcium Uptake? *Int J Mol Sci* **22**, doi:10.3390/ijms22020946 (2021).

7 Dewenter, M., von der Lieth, A., Katus, H. A. & Backs, J. Calcium Signaling and Transcriptional Regulation in Cardiomyocytes. *Circ Res* **121**, 1000-1020, doi:10.1161/CIRCRESAHA.117.310355 (2017).

8 Morkin, E. Control of cardiac myosin heavy chain gene expression. *Microsc Res Tech* **50**, 522-531, doi:10.1002/1097-0029(20000915)50:6<522::AID-JEMT9>3.0.CO;2-U (2000).

9 Min, C. K. *et al.* Coupling of ryanodine receptor 2 and voltage-dependent anion channel 2 is essential for Ca(2)+ transfer from the sarcoplasmic reticulum to the mitochondria in the heart. *Biochem J* **447**, 371-379, doi:10.1042/BJ20120705 (2012).

10 Schweitzer, M. K. *et al.* Suppression of Arrhythmia by Enhancing Mitochondrial Ca(2+) Uptake in Catecholaminergic Ventricular Tachycardia Models. *JACC Basic Transl Sci* **2**, 737-747, doi:10.1016/j.jacbts.2017.06.008 (2017).

11 Zhou, P. & Pu, W. T. Recounting Cardiac Cellular Composition. *Circ Res* **118**, 368-370, doi:10.1161/CIRCRESAHA.116.308139 (2016).

12 Chiong, M. *et al.* Mitochondrial metabolism and the control of vascular smooth muscle cell proliferation. *Front Cell Dev Biol* **2**, 72, doi:10.3389/fcell.2014.00072 (2014).

13 Shin, J., Song, I. S., Pak, J. H. & Jang, S. W. Upregulation of annexin A1 expression by butyrate in human melanoma cells induces invasion by inhibiting E-cadherin expression. *Tumour Biol* **37**, 14577-14584, doi:10.1007/s13277-016-5306-5 (2016).

14 Shi, Y., Felley-Bosco, E., Marti, T. M. & Stahel, R. A. Differential effects of lovastatin on cisplatin responses in normal human mesothelial cells versus cancer cells: implication for therapy. *PLoS One* **7**, e45354, doi:10.1371/journal.pone.0045354 (2012).

15 Weiss, A. *et al.* Organization of human and mouse skeletal myosin heavy chain gene clusters is highly conserved. *Proc Natl Acad Sci U S A* **96**, 2958-2963, doi:10.1073/pnas.96.6.2958 (1999).

16 Stanley, W. C., Lopaschuk, G. D., Hall, J. L. & McCormack, J. G. Regulation of myocardial carbohydrate metabolism under normal and ischaemic conditions. Potential for pharmacological interventions. *Cardiovasc Res* **33**, 243-257, doi:10.1016/s0008-6363(96)00245-3 (1997).

17 Zhang, P., Shan, T., Liang, X., Deng, C. & Kuang, S. Mammalian target of rapamycin is essential for cardiomyocyte survival and heart development in mice. *Biochem Biophys Res Commun* **452**, 53-59, doi:10.1016/j.bbrc.2014.08.046 (2014).

18 Major, J. L., Dewan, A., Salih, M., Leddy, J. J. & Tuana, B. S. E2F6 Impairs Glycolysis and Activates BDH1 Expression Prior to Dilated Cardiomyopathy. *PLoS One* **12**, e0170066, doi:10.1371/journal.pone.0170066 (2017).

REVIEWER COMMENTS

Reviewer #1 (Remarks to the Author):

The authors have addressed my comments. Yet, I am not happy with the quality of the western blotting. For example, in Figure 2c and Supplemental Figure 2a, the differences within the WT samples are the same as between the WT and the KO.

The findings that VDAC2 plays a crucial role during cardiac development by influencing cellular calcium signaling, is important and should be published.

Reviewer #2 (Remarks to the Author):

The authors addressed all my points and the revised version improved.
I have a new remark related to the AAV9 experiments. The authors should write the dose injected (vg/kg) and not the GC/ml.

Reviewer #3 (Remarks to the Author):

This manuscript is a resubmission regarding the role of VDAC2 in the heart, by linking cardiac-specific deletion of Vdac2 in mice resulted to a DCM phenotype with aberrant calcium homeostasis and ECC. The authors provide a persuasive argument that VDAC2 plays a crucial role during cardiac development and function. The resubmission is greatly improved over the original manuscript. The reviewer appreciates the attention to detail in answering questions with appropriate experiments and statistical analyses. In particular, the assessment of functional rescue with AAV9-aMHC-VDAC2 increases support for the authors' argument.

We thank the reviewers and editors for their careful review of our manuscript and for their suggestions. We have put more attention to the western blot figures and now replaced all main figures and supplemental figures to better represent the source data.

REVIEWER COMMENTS

Reviewer #1 (Remarks to the Author):

The authors have addressed my comments. Yet, I am not happy with the quality of the western blotting. For example, in Figure 2c and Supplemental Figure 2a, the differences within the WT samples are the same as between the WT and the KO.
The findings that VDAC2 plays a crucial role during cardiac development by influencing cellular calcium signaling, is important and should be published.

Response 1: We really appreciate the reviewer's concern regarding the variability observed within the WT samples and between the WT and KO samples. We would like to clarify that even though this is true, in most cases, the down regulation of proteins observed in our KO (shown in the western blot) is supported by our RNA sequencing data as well. Furthermore, we now present the western blot figures from all 6 samples throughout the manuscript and in supplemental figures.

Reviewer #2 (Remarks to the Author):

The authors addressed all my points and the revised version improved.
I have a new remark related to the AAV9 experiments. The authors should write the dose injected (vg/kg) and not the GC/ml.

Response 1: We really appreciate this suggestion by the reviewer. We injected 100µl of 5x10e10 GC/ml and the average weight of mice injected was 16.1g which gives us 3.1x10e12 vg/kg. This has now been clarified in the manuscript in Page 15, Line 383.

Reviewer #3 (Remarks to the Author):

This manuscript is a resubmission regarding the role of VDAC2 in the heart, by linking cardiac-specific deletion of Vdac2 in mice resulted to a DCM phenotype with aberrant calcium homeostasis and ECC. The authors provide a persuasive argument that VDAC2 plays a crucial role during cardiac development and function. The resubmission is greatly improved over the original manuscript. The reviewer appreciates the attention to detail in answering questions with appropriate experiments and statistical analyses. In particular, the assessment of functional rescue with AAV9-aMHC-VDAC2 increases support for the authors' argument.

Response 1: We really appreciate this comment and thank the reviewer for helping us improve the manuscript.
